# Single Cell and Spatially Resolved Transcriptome and Immune Repertoire of Mouse Thymus During Aging Reveal Immunological Heterogeneity and Direction of Thymic Selection Pressure

**DOI:** 10.1111/acel.70631

**Published:** 2026-07-25

**Authors:** Jialing Fang, Jun Lei, Peng Chen, Zaiqiao Sun, Boxiao He, Yankang Wu, Chonil Paek, Ning Wu, Yafei Huang, ZhaoKui Dan, Hui Zhang, Zijing Xu, Pengcheng Wei, Yongshun Chen, Lei Yin

**Affiliations:** ^1^ Department of Pediatric Research Institute Children's Hospital of Chongqing Medical University, National Clinical Research Center for Children and Adolescents' Health and Diseases, Ministry of Education Key Laboratory of Child Development and Disorders, Chongqing Key Laboratory of Child Rare Diseases in Infection and Immunity Intelligent Application of Big Data in Pediatrics Engineering Research Center of Chongqing Education Commission of China Chongqing China; ^2^ Liangzhu Laboratory Zhejiang University Hangzhou Zhejiang China; ^3^ Bone Marrow Transplantation Center of the First Affiliated Hospital, and Center for Stem Cell and Regenerative Medicine Zhejiang University School of Medicine Hangzhou Zhejiang China; ^4^ Hubei Province Key Laboratory of Allergy and Immunology Wuhan University Wuhan China; ^5^ Department of Laboratory Medicine Hangzhou Xixi Hospital, Hangzhou Sixth People's Hospital, Hangzhou Medical College Hangzhou China; ^6^ Cancer Center, the Eighth Affiliated Hospital Sun Yat‐Sen University Shenzhen China; ^7^ Hubei Key Laboratory of Cell Homeostasis, State Key Laboratory of Virology, College of Life Sciences Wuhan University Wuhan China; ^8^ Department of Immunology, School of Basic Medicine, Tongji Medical College Huazhong University of Science and Technology Wuhan China; ^9^ Clinical Medicine School of Hubei University of Science and Technology Xianning China; ^10^ Kindstar Global Precision Medicine Institute Wuhan China; ^11^ Wuhan Kindstar Biotech Technology Co., Ltd Wuhan China; ^12^ Guangxi Key Laboratory of Special Biomedicine, School of Medicine Guangxi University Nanning China

**Keywords:** immune repertoire, scRNA‐seq, scTCR‐seq, spatial‐TCR‐seq, ST‐seq, thymic selection pressure, thymus, thymus aging

## Abstract

The thymus is the central organ where T cells differentiate and proliferate. T cells experience the TCR recombination to generate diversity, self‐antigen tolerance, and immune response. However, the spatial heterogeneity of transcriptomics and immune repertoire of the thymus during aging haven't been studied. We constructed the thymus's spatial transcriptomics and spatial immune repertoire atlas by TCR‐specific amplification. The integrated multi‐omics analysis of the scRNA‐seq, ST‐seq, spatial‐TCR‐seq, scTCR‐seq, and TCR‐seq was developed to investigate the thymus during aging. ST‐seq demonstrated that the heterogeneity of the cortex and a novel cortex subset associated with progenitor cell differentiation was identified. Proliferating CD69negDN T cells decreased and regulatory CD4SP T cells increased during aging. Using deconvolution analyses, the spatial distribution of T cell subsets was revealed. The spatial distribution of TCR recombination and distinct thymic selection processes were illustrated. Pseudo‐time analyses demonstrated differentiation order and spatial migration route of T cells. The novel spatial‐TCR‐seq demonstrated the overall spatial distribution of TCR, the TCR pairing process, and their dynamic features under thymic selection pressure which revealed that TRB clonotypes with hydrophilic and low‐pI CDR3s tended to pass the negative selection opposite to the positive selection. The positive selection pressure of the thymus diminishes during the aging process. A TCR maturation scoring system using TCR‐seq was developed to identify TCR clonotypes that tended to mature. All boost the thymus study into the era of spatial immune repertoire and transcriptome and widen our knowledge about T cell development.

## Graphical abstract

A systemic single‐cell and spatially‐resolved transcriptomics and TCR sequencing analysis across the aging thymus dissects the molecular underpinnings of T cell subset dynamics. The study highlights the shifting proportions and transcriptomic profiles of T cell subsets during aging. It elucidates the connection between spatial localization and functional heterogeneity in the thymus, revealing two distinct spatial differentiation pathways. Additionally, using a novel spatial‐TCR‐seq approach, the study constructs a thymus spatial TCR atlas and demonstrates the opposing selection pressures of thymic positive and negative selections as well as their dynamics during aging.

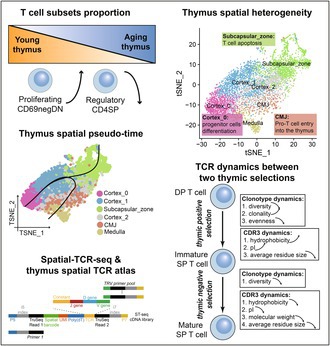

**Abbreviations**
AA: amino acid
CDR3: complementarity‐determining region 3
CDR3βmr: CDR3β middle region
GO: Gene Ontology
MDS: multi‐dimensional scaling
scRNA‐seq: single‐cell RNA sequencing
scTCR‐seq: single‐cell T‐cell receptor sequencing
spatial‐TCR‐seq: spatial TCR sequencing
ST‐seq: spatial transcriptomics sequencing
TCR: T cell receptor
TCR‐seq: T cell receptor sequencing
TEC: thymic epithelial cell
TF: transcription factor
TSC: thymic stromal cell
UMI: unique molecular identifier

## 1 Background

The thymus is the central organ where T cells differentiate and proliferate. During the development of the thymus, T cell progenitors arrive from the peripheral blood which is regulated by the opening of the thymus ‘gate’ (Ladi et al. 2006; Rossi et al. 2005; Petrie 2003) and experience a series of migration, proliferation, and differentiation in the thymus and return to the circulation environment as mature naïve T cells (Ladi et al. 2006; Cyster 2005). Each mouse individual has about 1–2 × 10^8^ T cells displaying about 2 × 10^6^ different TCRs (Goronzy and Weyand 2005; Casrouge et al. 2000). Most thymocytes get apoptotic during thymic selection (Nikolich‐Zugich et al. 2004) and only a few thymocytes survive (Stritesky et al. 2013). The hypoplasia or absence of the thymus results in T cells and cellular immune function loss. During development in the thymus, T cells experience TCR recombination and thymic selection. T cell progenitors experience rearrangement of both α and β chains of TCR in the thymus (James et al. 2018; Boudil et al. 2015) which are regulated by multiple signatures (Seo and Taniuchi 2016; Deftos et al. 1998; Gifford and Meissner 2012). TCRαβ heterodimers (Bhakta et al. 2005) are expressed on the cell surface and exposed to potential ligands in their environment for selection. Immature double‐positive (DP) T cells interact with the self‐antigen‐MHC complex expressed on the surface of thymic stromal cells (TSCs) such as thymic epithelial cells (Palmer 2003; van Meerwijk et al. 1997; Cosway et al. 2017) and immune‐related cells such as dendritic cells and macrophages (Hogquist et al. 2005) to become single‐positive (SP) T cells (Kishimoto and Sprent 1997). Only T cells with proper affinity survive (Au‐Yeung et al. 2014; McNeil et al. 2005) while those with affinity too strong or too weak get apoptotic (Au‐Yeung et al. 2014; Dzhagalov et al. 2013). After thymic positive and negative selection, T cells become naïve T cells and reside in peripheral immune organs. The differentiation of T cells in the thymus is precisely regulated by complex signaling pathways (Weber et al. 2011; Tomita et al. 1999; Maillard et al. 2006; Germar et al. 2011; Willert and Jones 2006; Jarriault et al. 1995; Wendorff et al. 2010). Thymus repertoire is highly linked to body immunity. T cells with self‐reactive TCR escaping thymic selection would cause autoimmune diseases while tumor's antigen‐associated T cell clonotypes coming from the thymus would help to fight cancers. However, the spatial heterogeneity of the thymus from the transcriptional perspective and TCR repertoire during aging are short of study in the field.

Single‐cell RNA sequencing (scRNA‐seq) has facilitated the mapping of organ development at unprecedented resolution and revealed the heterogeneity of diverse tissues, identifying cell populations and investigating the transcriptional features of distinct populations (Zheng et al. 2017). scTCR‐seq is utilized to illustrate the immune repertoire dynamics of thymocyte subpopulations. Spatial transcriptomics sequencing (ST‐seq) can map transcriptional signatures to distinct geographical regions with specific histological significance (Ståhl et al. 2016; Marx 2021).

The spatial transcriptomics of the thymus and spatial distribution of immune repertoire during aging have not been determined yet. This research investigated the integrated scRNA‐seq, ST‐seq, spatial‐TCR‐seq, scTCR‐seq, and TCR‐seq of thymuses during aging. We demonstrated the heterogeneity of cell types and their dynamics during aging which revealed that CD4SP T cells' proportion increased and CD69negDN T cells' proportion decreased. γδ T cell activation, CD4 T cell differentiation, T cell adaptive immune response, and thymic negative selection were promoted while TCR recombination and thymic positive selection were suppressed in aging thymuses. In ST‐seq datasets of distinct ages, 6 sub‐structures were identified with specific spatial distributions and transcriptional features. The heterogeneity of the cortex was illustrated and the cortex_0 was identified as a novel region associated with the progenitor T cell differentiation. T‐cell apoptosis mainly occurred in the subcapsular zone. The spatial distribution of T cell subsets was illustrated using deconvolution. The pseudo‐time analyses of scRNA‐seq and ST‐seq datasets revealed the differentiation of T cell subsets as well as their spatial migration. CD4SP T cells appeared earlier than CD8SP T cells. It was shown that T cells that didn't successfully rearrange TCR get apoptotic in the subcapsular zone. Spatial immune repertoire analysis revealed the spatial distribution of clonotypes as well as their dynamics among distinct sub‐structures and during TCR‐involved processes such as TCR recombination, TCR chain pairing, and thymic selection. TCR beta chain CDR3s displayed an opposite trend in hydrophobicity and pI between positive and negative selection. Thymuses and PBMCs were compared to demonstrate how thymic selection generally influenced the immune repertoire. Furthermore, a TCR maturation scoring system based on a single‐level logistic regression model was also developed to identify TCR clonotypes that could mature and export from the thymus.

## 2 Results

### 2.1 Single‐Cell Transcriptomics Reveals the Heterogeneity of the Mouse Thymus During Aging

**FIGURE 1:**
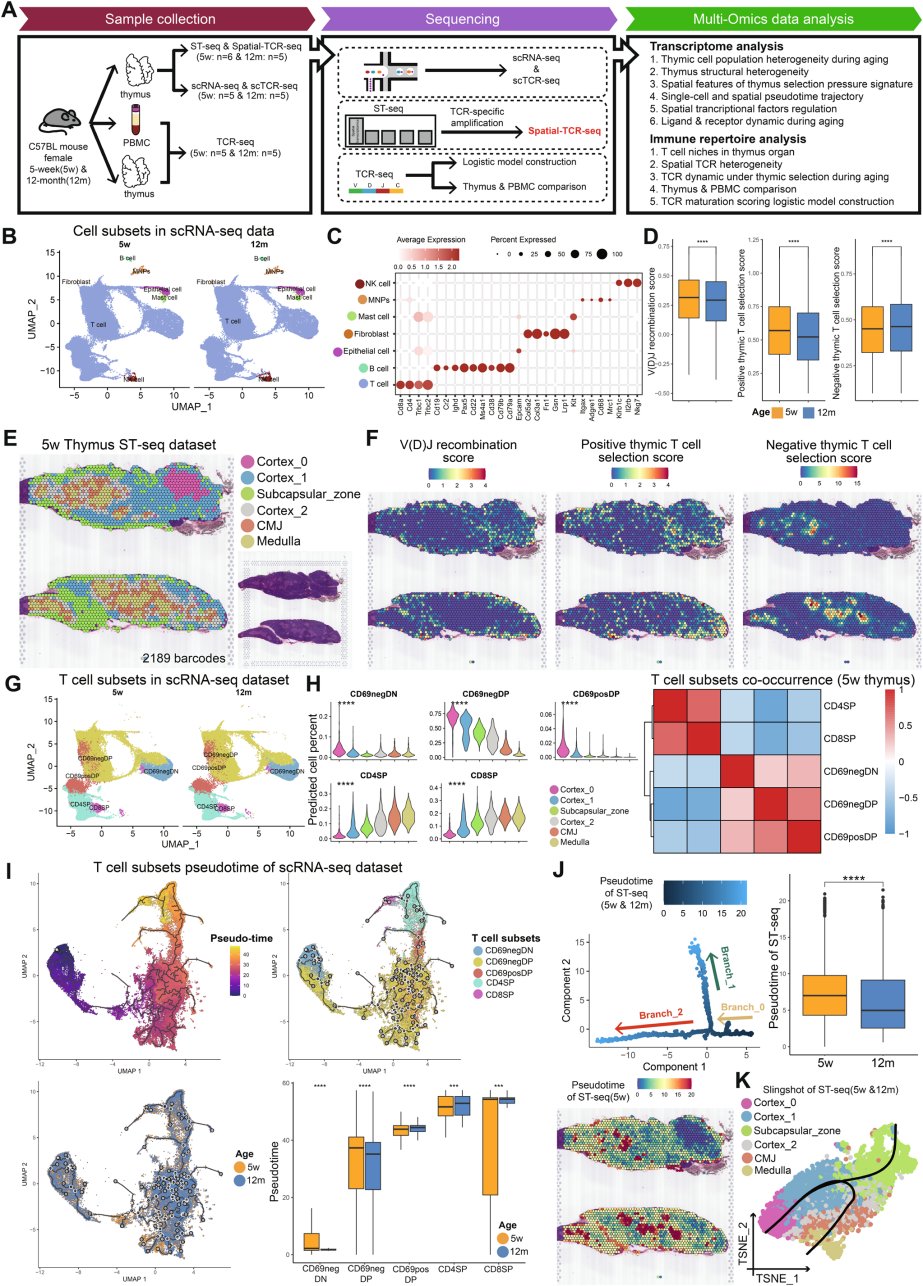
ST‐seq and scRNA‐seq of the thymus demonstrated the heterogeneity and the development route of T cells. (A) Workflow of the mouse thymus samples and PBMC samples processing for multi‐omics integrative analyses including ST‐seq, spatial‐TCR‐seq, scRNA‐seq, scTCR‐seq, and TCR‐seq to illustrate the spatial heterogeneity of the transcriptome and immune repertoire as well as the immune repertoire dynamic during thymic selection pressure. (B) The uniform manifold approximation and projection (UMAP) of scRNA‐seq barcodes recovered from the thymus sample of distinct ages labeled by cell types. (C) The dot plot for expression of marker genes in distinct cell populations. Color represents the maximum‐normalized mean expression of marker genes in each cell type, and size indicates the proportion of cells expressing marker genes. (D) Box plots indicating the expression of several signatures in distinct ages of mouse thymuses. A *t*‐test was utilized to calculate the statistical significance (ns: *p* > 0.05; *: *p* < 0.05; **: *p* < 0.01; ***: *p* < 0.001; ****: *p* < 0.0001). (E) Clustering of ST spots based on the tissue regions and annotated with distinct histological sub‐structures (left and right top) and the histology stain of the 5‐week thymus (right bottom). (F) Spatial feature plots of several vital signature pathways including V(D)J recombination, positive thymic T cell selection, and negative thymic T cell selection of the 5‐week thymus. (G) The uniform manifold approximation and projection (UMAP) of T cell subsets of scRNA‐seq barcodes recovered from thymuses of distinct ages labeled by cell types. (H) Left, violin plots of predicted percent of distinct T cell subsets in sub‐structures of the 5‐week thymus. Right, the heatmap indicating the same‐spot co‐occurrence of distinct T cell subsets of the 5‐week thymus. The ANOVA was used to calculate the significance (ns: *p* > 0.05; *: *p* < 0.05; **: *p* < 0.01; ***: *p* < 0.001; ****: *p* < 0.0001). (I) The pseudo‐time trajectory order of T cell subsets in the scRNA‐seq datasets (top left). The UMAP of T cell subsets which corresponded to the trajectory (top right). The trajectory order of samples of distinct ages and the box plot of the trajectory of samples of distinct ages (bottom left). The box plot of the trajectory of distinct T cell subsets split by age (bottom right). A *t*‐test was utilized to calculate the statistical significance (ns: *p* > 0.05; *: *p* < 0.05; **: *p* < 0.01; ***: *p* < 0.001; ****: *p* < 0.0001). (J) Top left, the pseudo‐time trajectory order of ST‐seq thymus samples which was annotated with three branches. Top right, the box plot of the trajectory of distinct thymus samples. Bottom left, the spatial distribution of the pseudo‐time trajectory of the 5‐week thymus using Monocle2. A *t*‐test was utilized to calculate the statistical significance (ns: *p* > 0.05; *: *p* < 0.05; **: *p* < 0.01; ***: *p* < 0.001; ****: *p* < 0.0001). (K) The predicted differentiation route of T cells in the thymus of ST‐seq datasets using Slingshot.

Mouse thymuses of distinct ages were processed to investigate the single‐cell transcriptional landscape using the Mozhuo platform and the overall workflow was presented (Figure 1A). After preprocessing including quality control (Figure S1A) and cell cycle regression (Figure S1B), the cell populations of the scRNA‐seq dataset were annotated (Figures 1B and S2A) based on several marker genes from reference datasets (Xu et al. 2021) (Figure 1C). The thymus is mainly composed of T cells (Figure S2B) and NK cells and B cells were significantly enriched in aging samples, which was opposite to mast cells and T cells (Figure S2C). Differential genes and signatures of aging samples versus young samples were calculated (Figure S2D,E). Aging samples were enriched in T cell apoptosis, T cell immune response including inflammatory response and MHC complex binding, and gamma‐delta T cell activation (Figure S2E). Progenitor cell differentiation, TCR recombination, and thymic T cell positive selection were suppressed while CD4 regulatory T cell differentiation and thymic T cell negative selection were more activated in aging samples (Figures 1D and S2F). We further tried to find out transcriptional features of each cell subset during the aging which showed similar results in Figure S2E (Figure S2G–I). NK cells displayed the highest number of up‐regulated DEGs, and B cells displayed the highest number of down‐regulated DEGs (Figure S2H).

### 2.2 Spatially Resolved Transcriptomics Reveals the Architecture of the Mouse Thymus During Aging

To further investigate the spatial heterogeneity of the mouse thymus during aging which had not been demonstrated before, the ST‐seq of the thymus using the 10× Genomics Visium platform (Salmén et al. 2018; Wang et al. 2021) was performed (Figure 1A). Quality control (Figure S1C,D) and cell cycle regression (Figure S1E) were utilized. ST‐seq datasets can be differentially labeled with six sub‐structures by unsupervised reduction (Figure S3A) which properly matched the interface, the cortex, and the medulla acquired from histological staining (Figure 1E). Sub‐structures were annotated based on differential genes enriched in specific signaling pathways (Figure S3B). The heterogeneity of the cortex was demonstrated and a novel cortex subset named Cortex_0 associated with the differentiation of progenitor cells was identified (Figure S3B). The proportion of Corticomedullary junction (CMJ) and medulla decreased while the proportion of Cortex_0 and subcapsular zone increased in the aging sample (Figure S3C). The cortex was heterogeneously divided into three subsets around the subcapsular zone which was consistent with former researches that T cells migrate through the cortex towards the subcapsular zone for the apoptosis process of cells failing TCR pairing and then move back through the cortex towards the medulla to go through thymic positive selection (Heimli et al. 2022). Differential genes of each sub‐structure during aging were illustrated (Figure S3D). The medulla displayed the highest number of up‐regulated DEGs and the cortex_0 displayed the highest number of down‐regulated DEGs (Figure S3D right). Several signatures including antigen presentation, response to type II interferon, and inflammatory response were enriched in aging samples compared to young samples (Figure S3E).

The spatial distributions of sub‐structures (Figures 1E and S3F) and several vital signatures (Figures 1F and S3G) were demonstrated. Overall, the aging thymus seemed to show less clear sub‐structure compartmentalization than the young thymus, which was consistent with previous studies (Li and Zúñiga‐Pflücker 2023). The correlations of these signatures were calculated in each ST‐seq sample, which showed that the positive thymic selection was positively correlated with the negative thymic selection in both samples, while the V(D)J recombination was negatively correlated with the negative thymic selection only in the 5‐week sample (Figure S3H). It suggested that the spatial distribution of distinct biological functions was disrupted in aging samples.

The tissue depth of distinct sub‐structures of the 5‐week sample was calculated to illustrate the spatial distribution (Xu et al. 2021; Fawkner‐Corbett et al. 2021) (Figure S3I top left), which demonstrated the deep location of the medulla where the last stage of T cell development happened, and the subcapsular zone was mainly located in the shallow tissue. The differences in tissue depth of distinct cortexes suggested its heterogeneity (Figure S3I top right). We also calculated the proportion of the nearest 6 barcodes surrounding distinct barcodes to illustrate the spatial distribution and interactions of sub‐structures (Figure S3I bottom) that the Cortex_0 was only near Cortex_1 while Cortex_2 was near several sub‐structures. Similar analyses were performed in the 12‐month sample which illustrated the disrupted distribution of sub‐structures and suggested damaged biological functions (Figure S3J).

Since the ST‐seq of the 10× Genomics Visium platform was not single‐cell resolution, we further performed another spatial transcriptomics sequencing of young and aging thymus using the SeekSpace single‐cell spatial transcriptomics technology (young, 5w, *n* = 4; aged, 12 m, *n* = 3). Utilizing this method, similar cell populations (Figure S4A,B) and histological structures (Figure S4C,D) were identified. A similar Cortex_0 was identified which expressed high levels of several genes related to thymic progenitor cell migration (including Ccl25 and Ccr9) and CD69negDN marker (including Cxcr4) as well as several related signatures (Figure S4E). Differential gene analyses revealed that the Cortex_0 was enriched in the differentiation of progenitor cells and the TCR V(D)J recombination (Figure S4F). For each histological compartment we computed the minimal inter‐barcode distance among spatially distinct barcodes; this metric serves as an inverse proxy for structural cohesion. Consequently, smaller minimal distances indicate preserved architectural integrity, whereas progressively larger values reflect age‐dependent dispersion and fragmentation of thymic histological architecture. Dispersion and fragmentation of thymic histological architecture were observed in aging samples (Figure S4G), which suggested that the organized histological structure was disrupted in the aging sample. Utilizing the SeekSpace spatial dataset, it was also revealed that the Cortex_0 was spatially distributed near the CMJ (Figure S4H). To validate the existence of the cortex_0 in the human thymus, we downloaded human‐derived thymus spatial transcriptomics datasets (Li et al. 2023) and the similar cortex_0 sub‐structure was observed with specific spatial distribution (Figure S5).

The spatial distribution of cell subsets in the 5‐week thymus ST‐seq dataset was revealed utilizing the CARD deconvolution pipeline (Ma and Zhou 2022) (Figure S6A–C). B cells, MNPs (mononuclear phagocytes), and NK cells were more enriched in the CMJ and the medulla. It suggested their activated interactions with T cells at the terminal differentiation state (Figure S6A). The same‐spot co‐occurrence analysis illustrated the co‐existence of different cell subsets in a specific barcode (Stuart et al. 2019) (Figure S6B). The distributions of T cells and B cells were negatively correlated, consistent with the previous report that the increase of B cells resulted in the reduction of T cells (Xiao et al. 2018). The proportion of cell subsets was approved by the expression of several marker genes in the ST‐seq datasets (Figure S6C). Similar analyses were performed on the 12‐month thymus ST‐seq dataset (Figure S6D–F). The distribution of stromal cells including mast cells and epithelial cells as well as NK cells changed in the aging thymus compared to the young thymus (Figure S6D).

### 2.3 Analysis of Thymic T Cell Lineage Differentiation and Its Spatial Distribution During Aging

We further demonstrated the heterogeneity of T cell subsets during thymus aging (Figures 1G and S7A,B). There were no significant differences in the proportion of major T cell subsets during aging (Figure S7B,C). We then demonstrated transcriptional dynamics in T cell subsets (Figure S7E–G) which showed that CD4 regulatory T cell differentiation and thymic T cell negative selection were more enriched in aging samples, while progenitor cell differentiation, TCR recombination, and thymic positive selection were more enriched in young samples (Figure S7F). Furthermore, it was revealed that CD69negDP T cells in aging samples get apoptotic, which might be associated with endoplasmic reticulum stress and response to unfolded proteins (Figure S7G).

We tried to identify minor T cell Seurat clusters that ranged during aging and it was demonstrated that CD69negDN_14 cells significantly decreased and CD4SP_18 cells significantly increased during aging (Figure S7H). This result was consistent with the published research findings (Kozlowska et al. 2007; Li et al. 2024). CD8SP_26 cells exhibited a significant reduction, but their absolute abundance remained below 1% of total T cells (Figure S7I). Differential genes and enriched signatures of these two clusters were calculated (Figure S7J). Cell cycle‐related signatures were enriched in CD69negDN_14 cells and regulatory CD4 T cell differentiation was enriched in CD4SP_18 cells (Figure S7K). It was further validated that the T cell proliferation marker Mki67 was enriched in CD69negDN_14 cells and the regulatory T cell marker Foxp3 was enriched in CD4SP_18 cells (Figure S7L). We further calculated differentiation genes of these two clusters during aging (Figure S7M). During aging, Apoe (Lü et al. 2020) which was associated with reduced MHC‐dependent antigen presentation, and Zfp36l2 (Cook et al. 2022) which was associated with autoreactive T cell response were increased in CD69negDN_14 cells while Nkg7 (Li et al. 2022) and Il7r (Oliveira et al. 2022) which were associated with adaptive immune responses were increased in regulatory CD4SP_18 cells (Figure S7M left). This result suggested that the progenitor cell‐like cell state has weakened, while the self‐reactivity of CD69negDN T cells has increased, and thus they were eliminated during the thymus selection process. Il7r was absent in Tregs of young samples but activated in aging samples (Figure S7M right). It was shown that CD69negDN_14 cells were specifically located at the Cortex_0 while CD4SP_18 cells were mainly located at the CMJ and the medulla (Figure S7N) using the CARD prediction (Ma and Zhou 2022).

The spatial distribution of major T cell subsets in the 5‐week thymus was also demonstrated (Figures 1H left and S8A). CD69negDN and CD69negDP subsets were located across the outer tissue (Figure 1H left), which corresponded to the distribution of TCR recombination (Figure 1F). CD69posDP cells were mainly located in the Cortex_1 and the Cortex_0, where increased expression of Cd69 occurred (Figure S6C). The co‐existence of CD8SP and CD4SP T cells was also illustrated (Figure 1H right). The distribution of T cell subsets in the 12‐month sample was demonstrated without specific distribution features (Figure S8C–E). Several marker genes were highly expressed in the subcapsular zone (Figure S8E).

The current understanding is that the hematopoietic stem cells enter the thymus from capillaries around the CMJ. To ensure the T cell subsets distribution accuracy, we calculated the expression level of related signatures (Ashburner et al. 2000; Aleksander et al. 2023) which were highly expressed in the Cortex_0 which was associated with the differentiation of progenitor cells in the thymus (Figure S9A). Several genes including Cxcr4, Ccr9, and Ccl25 which were related to the migration of progenitor T cells into the thymus (Takahama 2006) were also highly expressed in the Cortex_0 (Figure S9B). CD69negDN T cells were enriched in the Cortex_0 (Figure S9C) utilizing several marker genes of CD69negDN T cells acquired from the previous study (Park et al. 2020).

The heterogeneity of CD69negDN T cells was shown (Figure S10A), revealing that CD69negDN_4 exhibited high expression of the early phase marker, Cd44, while other CD69negDN subsets expressed the late phase marker, Il2ra (Figure S10B). It was revealed that the CMJ showed a relatively high distribution of the CD69negDN_4 subset, whereas other subsets were predominantly located in the Cortex_0 (Figure S10C). Cortex_2 and medulla were spatially closed to the CMJ and thus showed a similar CD69negDN_4 signature. Co‐occurrence analyses illustrated the co‐existence of distinct CD69negDN subsets (Figure S10D). The differentiation of distinct CD69negDN subsets was demonstrated utilizing pseudo‐time analyses (Figure S10E,F). Above all, it could be assumed that several progenitor cells entered the thymus from capillaries around the CMJ but resided in the Cortex_0 for further differentiation processes.

To ensure the accuracy of the distribution of CD69posDP cells which has not been investigated, we calculated the enrichment score of CD69posDP T cells using several marker genes acquired from previous research (Domínguez Conde et al. 2022) (Figure S9D left). The CD69posDP cell score composed of several marker genes of CD69posDP T cells from the Cell Marker database (Hu et al. 2023) was shown in the thymus ST‐seq dataset (Figure S9D right). Besides, it was shown that CD69posDP T cell enrichment scores acquired from previous research and the database were significantly positively correlated with the CD69posDP T cells predicted percent (Figure S9E). Above all, CD69posDP T cells were enriched in the Cortex_0.

### 2.4 Pseudo‐Time Trajectory Analysis and TFs Identification Associated With Gene Expression Features During Thymocyte Migration and Differentiation

To demonstrate the differentiation as well as the spatial migration of T cells within the mouse thymus during aging, we performed the single‐cell pseudo‐time trajectory analyses of the T cell subset utilizing the Monocle3 (Cao et al. 2019) (Figure 1I top left). T cell subsets were linearly distributed in the trajectory (Figures 1I top right and S11A), which suggested that CD4SP T cells appeared earlier than CD8SP T cells. T cell subsets showed different pseudo‐time trajectory dynamics during aging, which showed that the differentiation of CD69neg T cell subsets was slightly arrested while SP T cell subsets were not severely influenced (Figure 1I bottom and S11B).

The spatial trajectories of the ST‐seq dataset of the thymus demonstrated the T cell developmental order among sub‐structures. The pseudo‐time trajectory consisted of three branches (Figures 1J top left and S12A). The T cell differentiation was arrested in the aging sample (Figures 1J top right and S12B). Barcodes of early differentiation events which were located closely to each other in the embedding graph (Figure S12C) should have similar gene expression patterns and changes. Barcodes of later maturation stages which were located distantly to each other should have diverse gene expression patterns and changes resulting from the terminal CD4/CD8 lineage differentiation. The location of sub‐structures in the trajectory indicated the migration pathway in the thymus (Figure S12C left). Thymocytes with distinct differential fates migrated to the medulla or the subcapsular zone (Figure S12C right). It was shown that the CMJ also partially located in the early phase of the trajectory which also corresponded to the entry of T cells through the CMJ (Figure S12C left). The medulla was exclusively located in the late trajectory order while the cortex was located in the early and middle trajectory order, which was consistent with the fact that thymocytes migrated from the cortex to the medulla during differentiation. The trajectory of sub‐structures between samples was demonstrated (Figure S12D). In the aging sample, the subcapsular zone pseudo‐time was promoted while the medulla pseudo‐time was arrested. The Cortex_1 was detected along all three branches, possibly indicating that the Cortex_1 retained a higher degree of T cell developmental plasticity or was the important route during T cell migration. The subcapsular zone was predominately located in the branch_2 while the medulla was enriched in the branch_1 (Figure S12E). The pseudo‐time trajectory of each spatial barcode was mapped into the histological (Figures 1J bottom and S12F). The trajectory analysis using Slingshot (Street et al. 2018) also found a node located in the Cortex_1 and two routes leading to the subcapsular zone or the medulla within the trajectory structure (Figure 1K). This analysis suggested that T cells in the Cortex_1 underwent migration to either the subcapsular zone or the medulla during differentiation. CD69negDN and CD69negDP T cells decreased while CD8SP and CD4SP T cells increased during the pseudo‐time trajectory (Figure S12G left). CD69posDP T cells were restrictedly located in the early pseudo‐time trajectory which suggested that the activation of T cells occurred in the early trajectory (Figure S12G right).

Each trajectory branch had transcriptional features (Figure S12H–J). Branch_0 at the early order was enriched in genes for cell proliferation and TCR recombination. Branch_1 was enriched in genes for T cell thymic selection, T cell immune effector, and the differentiation of NKT cell as well as alpha‐beta T cell. Branch_2 was enriched in genes for the apoptosis process and protein ubiquitination. The above results suggested that the subcapsular zone was mainly enriched in branch_2. The correlation between the trajectory order and several critical signaling pathways also supported this (Figure S12J).

Combined with the key motifs of sub‐structures identified by the SCENIC package (Aibar et al. 2017) (Figure S13A–C), we demonstrated the activity and openness of motifs during the pseudo‐time trajectory (Figure S13D) and identified that TF (transcription factor) Maz was specifically activated in the subcapsular zone (Figure S13E) and positively correlated with T cell apoptosis (Figure S13F), which might be mediated by protein ubiquitination (Figure S13G). This result was also supported by the published thymocytes scRNA‐seq dataset (Karimi et al. 2021) (Figure S13H).

### 2.5 Increased Interaction Strength Among B Cells, NK Cells, and CD69negDP T Cells Mediated by MHC Signature During Aging

During the differentiation of T cells in the thymus, T cells interacted with thymic stromal cells to gain self‐antigen tolerance and a functional immune response. The interaction strength between receptors and ligands was supposed to mediate the differentiation fate of T cells. Thus, we tried to demonstrate the differences in the ligand & receptor interaction strength between T cells and stromal cells during aging using CellChat (Jin et al. 2021) with the scRNA‐seq dataset. It was shown that the interaction strength increased in aging thymuses (Figure S14A). We focused on the interaction between T cells and stromal cells and demonstrated that interaction strength between CD69negDP T cells and B cells as well as NK cells increased significantly (Figure S14B). It was identified that the outgoing strength of MHC‐I increased in NK cells, the outgoing strength of MHC‐II increased in B cells, and the incoming strength of both MHC‐I and MHC‐II increased in the CD69negDP T cells (Figure S14C), which was validated by the ligand & receptor interactions of each signature (Figure S14D). Increased ligand and receptor interaction patterns were identified and displayed distinct interactions between CD69negDP T cells and B cells or NK cells (Figure S14E). T cells that didn't get mature became apoptotic during the differentiation. Notably, The Fasl–Fas interaction associated with T cell apoptosis was enriched between NK cells and CD69negDP T cells and increased during the aging (Figure S14F). At last, it was identified that NK cells became the main sender of the MHC‐I signature while B cells became the main sender of the MHC‐II signature in aging thymuses (Figure S14G).

### 2.6 The Spatial Distribution of TCR Clonotypes Determined by the Novel Spatial‐TCR‐Seq

**FIGURE 2:**
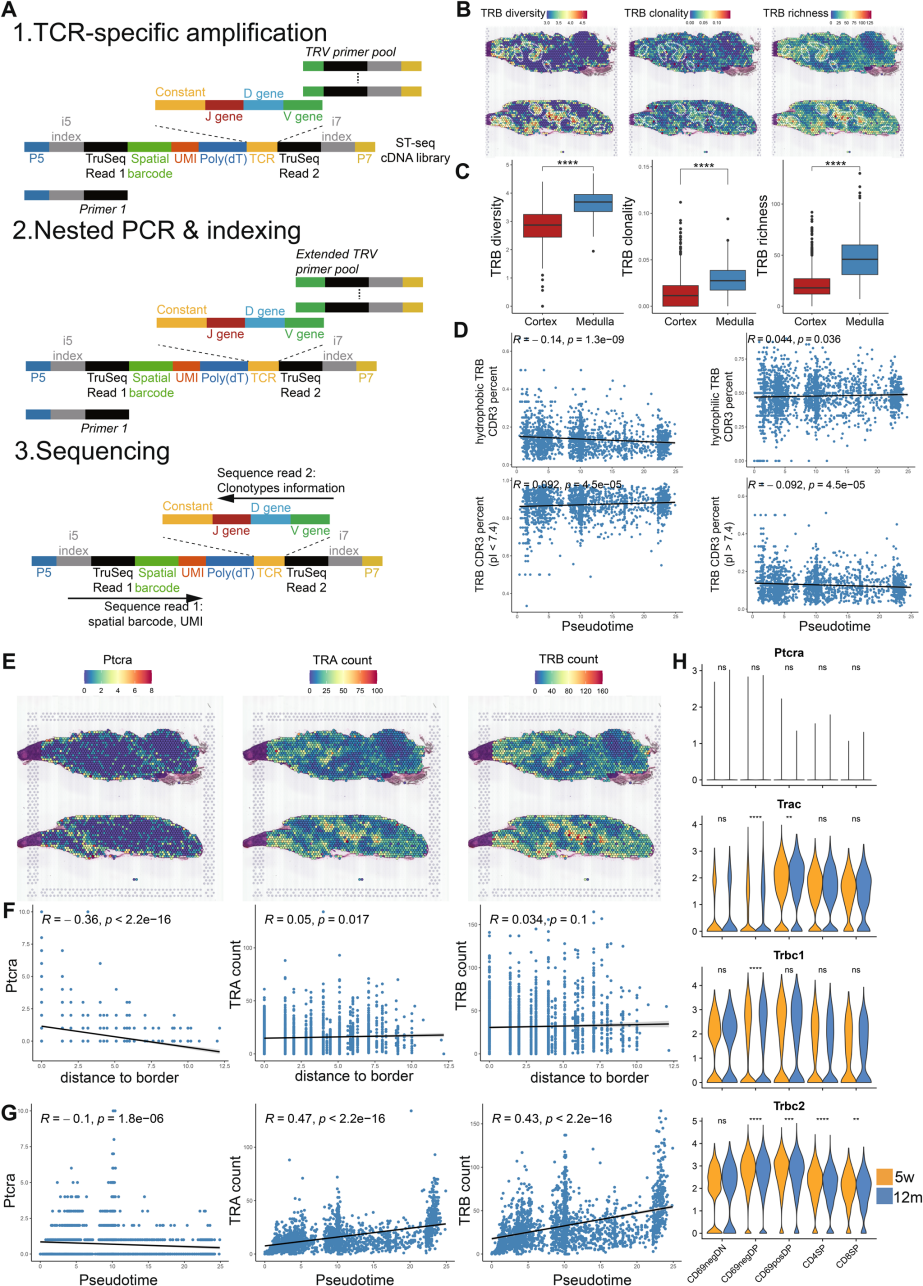
The spatial‐TCR‐seq demonstrated the spatial distribution of TCR and its pairing pattern. (A) Workflow of the spatial‐TCR‐seq including generation and the sequencing of the cDNA library containing the TCR information as well as the spatial information. (B) Spatial feature plots of calculated diversity, clonality, and richness of the TRB immune repertoire. All parameters were calculated using the VDJTools pipeline. (C) Box plots of diversity, clonality, and richness of individual spots derived from both spatial‐TCR‐seq and ST‐seq data by the medulla and the cortex. The cortex means the combination of all three cortex subsets. A *t*‐test was utilized to calculate the statistical significance (ns: *p* > 0.05; *: *p* < 0.05; **: *p* < 0.01; ***: *p* < 0.001; ****: *p* < 0.0001). (D) The scatterplot and correlation of the TRB CDR3aa frequency of distinct physicochemical properties including hydropathicity and electrostatic charge derived from both spatial‐TCR‐seq and ST‐seq data with the pseudo‐time trajectory. Hydrophobicity index < −0.25 means hydrophilic CDR3s. Hydrophobicity index > 0.25 means hydrophobic CDR3s. pI index > 7.4 means alkaline CDR3s which are positively charged. pI index < 7.4 means acidic CDR3s which are negatively charged. Significances were calculated with the *t*‐test method and correlations were calculated with the spearman method. (E) Spatial feature plots of the pre‐TCRα locus, TCRα locus, and TCRβ locus in the tissue section. (F) The scatterplot and correlation of three distinct TCR locus types and the min distance to the border of the tissue section which indicates the tissue depth. Significances were calculated with the *t*‐test method and correlations were calculated with the spearman method. (G) The scatterplot and correlation of three distinct TCR locus types and the pseudo‐time trajectory indicate the differentiation of thymocytes. Significances were calculated with the *t*‐test method and correlations were calculated with the spearman method. (H) Violin plots indicating the expression of the pre‐TCRα locus, TCRα locus, and TCRβ locus among T cell subsets in the scTCR‐seq data. A *t*‐test was utilized to calculate the statistical significance (ns: *p* > 0.05; *: *p* < 0.05; **: *p* < 0.01; ***: *p* < 0.001; ****: *p* < 0.0001).

The immune repertoire is the key element for an immune organ such as the thymus. However, almost no studies have revealed spatial immune repertoire and even have them connected with spatial transcriptomics. Thus, a method to directly determine the location of the whole immune repertoire was developed based on the connection between spatial barcodes and immune repertoire information. Through the spatial TCR sequencing (spatial‐TCR‐seq) performed from the cDNA library of the ST‐seq, the spatial‐TCR repertoire was connected with spatial transcriptomics properly. The TCR loci in the cDNA library were specifically amplified by the primer pool based on the TRV (TCR variable) genes and the Illumina TruSeq Read 1 sequence on sequence 3′. Every TRV gene was amplified separately and sequenced in a mixture (Figure 2A). The UMI (unique molecular identifier) was utilized to eliminate the PCR reaction bias. There were 38543 TRA (TCR chainα) reads and 76344 TRB (TCR chainβ) reads recovered corresponding to the spatial barcodes on the visium assay in which 38212 (99.1%) TRA reads and 75935 (99.5%) TRB reads were under the tissue.

The TCR information was extracted utilizing the TRUST4 algorithm, rather than a direct measurement. Thus, we further performed the comparison of the TCR repertoire between the spatial‐TCR‐seq and bulk‐TCR‐seq of the same sample to validate the accuracy of the TRUST4 pipeline. It was revealed that the TCR repertoire in the spatial‐TCR‐seq was highly overlapped with the bulk‐TCR‐seq (Figure S15A left and middle). Most expanded clonotypes with large clone count were also detected in the bulk‐TCR‐seq dataset (Figure S15A right). We also compared the TCR repertoire extracted by TRUST4 and MiXCR in the spatial‐TCR‐seq. It was revealed that TRUST4 could detect several clonotypes that were not detected utilizing the MiXCR (Figure S15B left and middle). However, most expanded clonotypes with large clone count can be detected in both methods (Figure S15B right). Barcodes detecting recovered TCR variable genes also detected TCR constant region gene transcripts (Figure S15C) and TCR recovered positively correlated both with counts of TCR constant region genes transcripts (Figure S15D,E) and T cell marker genes (Figure S15F) in the ST‐seq dataset. Most UMI reads can only detect and recover one TCR read (Figure S15G). We also performed the quality control analysis of the TCR‐specific amplification (Figure S15H) and the spatial‐TCR‐seq datasets sequencing saturation (Figure S15I). The spatial distribution of TCR constant region gene transcripts and recovered TCR were similar (Figure S16A,B). The scRNA‐seq and scTCR‐seq were performed to validate the accuracy of the following conclusions. Nearly half of barcodes detected both TRA and TRB chains (single pair) while partial barcodes only detected one TRB chain (orphan VDJ) which was due to the pairing process between TRB chains and pre‐TRA chains during the TCR recombination (Figure S16J).

The TCR sequences were identified and assigned to a spatial location in the thymus. The PCA reduction was utilized to show the dynamics of the diversity and dispersion of clonotypes (Figure S17). The size of all circles indicating diversity and dispersion demonstrated that all parameters persistently contracted during the development while the diversity of TRB CDR3s increased in the subcapsular zone and medulla (Figure S17B). The shallow tissue and the medulla region detected more unique CDR3s than the cortex region, suggesting that the V(D)J recombination and the pressure resulting from the thymic selection influence the spatial distribution of clonotypes. The Shannon entropy‐based diversity, clonality, and richness of TRB and TRA of each barcode were calculated (Figures 2B and S18A). The medulla was higher in diversity, clonality, and richness (these indexes were further described in the Methods section) compared to the cortex (including Cortex_0, Cortex_1, and Cortex_2) (Figures 2C and S18B,C), which demonstrated that the immune repertoire was more even in the cortex resulting from the random TCR recombination and had increased clonality due to the selection pressure and proliferation in the medulla. The higher diversity in the medulla was due to the increase in the total number of clones (which was richness) and the expansion of clones (which was clonality). The correlation between immune repertoire and the pseudo‐time trajectory also supported this inference (Figure S18D).

Physicochemical features of CDR3s that influenced the interaction affinity between TCR and MHC molecules were analyzed. TCRα were more enriched in hydrophobic CDR3s while TCRβ were apparently in the opposite direction (Figure S18E top). It is also interesting that the pI of TCRα was higher compared to TCRβ (Figure S18E bottom). The correlation between the pseudo‐time and physicochemical features was also analyzed to indicate the physicochemical dynamic during the T cell development. In the early trajectory, the opposite physicochemical feature of TCRβ CDR3s and TCRα CDR3s already exists. In the late trajectory, hydrophilic and low pI CDR3s of TCRβ and hydrophobic and high pI CDR3s of TCRα were further enriched (Figures 2D and S18F).

### 2.7 Comparison of TCR Clonotypes Between T Cell Subsets of the Thymus Through scTCR‐Seq

There were no significant differences in the TRB repertoire during aging (Figure S19). Therefore, we compared the immune repertoire among T cell subsets. CD4SP and CD8SP T cells were enriched in specific TRB VJ gene patterns (Figure S20A,B) and shorter CDR3aa length (Figure S20C). Clonotypes were expanded in the CD69negDP which resulted from the proliferation after TCR recombination and pairing. Clonotypes were contracted in SP T cells which resulted from thymic selection pressure (Figure S20D). The PCA reduction showed that the diversity and dispersion of TRB VJ genes and CDR3aa length decreased during the T cell differentiation (Figure S20E). It was further demonstrated that overall hydrophobicity and pI of the TRB CDR3aa significantly decreased in SP T cells (Figure S20F).

### 2.8 TCR Pairing Associated With Specific Spatial Distribution and Transcriptomic Features

TCR recombination is the first vital biological process that occurs when T cells are at the progenitor cell stage and results in the diversity of the immune repertoire (Macedo et al. 1999; Jackson and Krangel 2006). The spatial distribution of TCR (Figure 2E) visually demonstrated that the pre‐TCRα transcripts were located more in the cortex as well as the subcapsular zone while the TCRα transcripts were more detected in the medulla experiencing thymic selection (Figures S21A and S16A bottom), which illustrated the appearance order between pre‐TCRα and TCRα. The tissue depth combined with the spatial distribution of pre‐TCRα, TCRα, and TCRβ (Figures 2F and S21B) suggested the TCRβ paired with both pre‐TCRα and TCRα in shallow tissue while mainly paired with TCRα in deeper tissue. The correlation between the trajectory order and TCR count also supported the conclusion (Figure 2G). The pre‐TCR composed of pre‐TCRα and TCRβ appeared at the early trajectory while the mature TCR structure composed of TCRα and TCRβ was at the late trajectory.

Barcodes were annotated based on the expression of the pre‐TCRα (Ptcra) (Figure S21C). Barcodes expressing pre‐TCRα were annotated as the Ptcra (+) region while the other barcodes were annotated as the Ptcra (−) region. Ptcra (+) region was mainly located in the cortex and subcapsular zone while Ptcra (−) region was located in both the cortex and the medulla (Figure S21D–F). TCRα and TCRβ were more detected in the Ptcra (+) region compared to the Ptcra (−) region (Figure S21G left), which resulted from the decreased intensity of T cells in the Ptcra (−) region (Figure S21G right). Distinct regions had transcriptional features and enriched signatures. Ptcra (+) region was related to pre‐TCR structure stability, TCR recombination, and T cell division. While the Ptcra (−) region was related to T cell thymic selection, inflammatory cytokine production, and innate immune response (Figure S21H,I). Similar conclusions were demonstrated from the scTCR‐seq dataset. Ptcra was highly expressed in CD69neg T cells compared to other T cell subsets (Figures 2H and S21K). Distinct TCR pairing patterns were annotated, which demonstrated that TCR pairing mainly occurred in CD69neg T cells (Figure S21L).

### 2.9 The Comparison of the Immune Repertoire Between the Thymus and PBMC Indicated the Overall Thymic Selection Pressure on TCR Clonotypes

**FIGURE 3:**
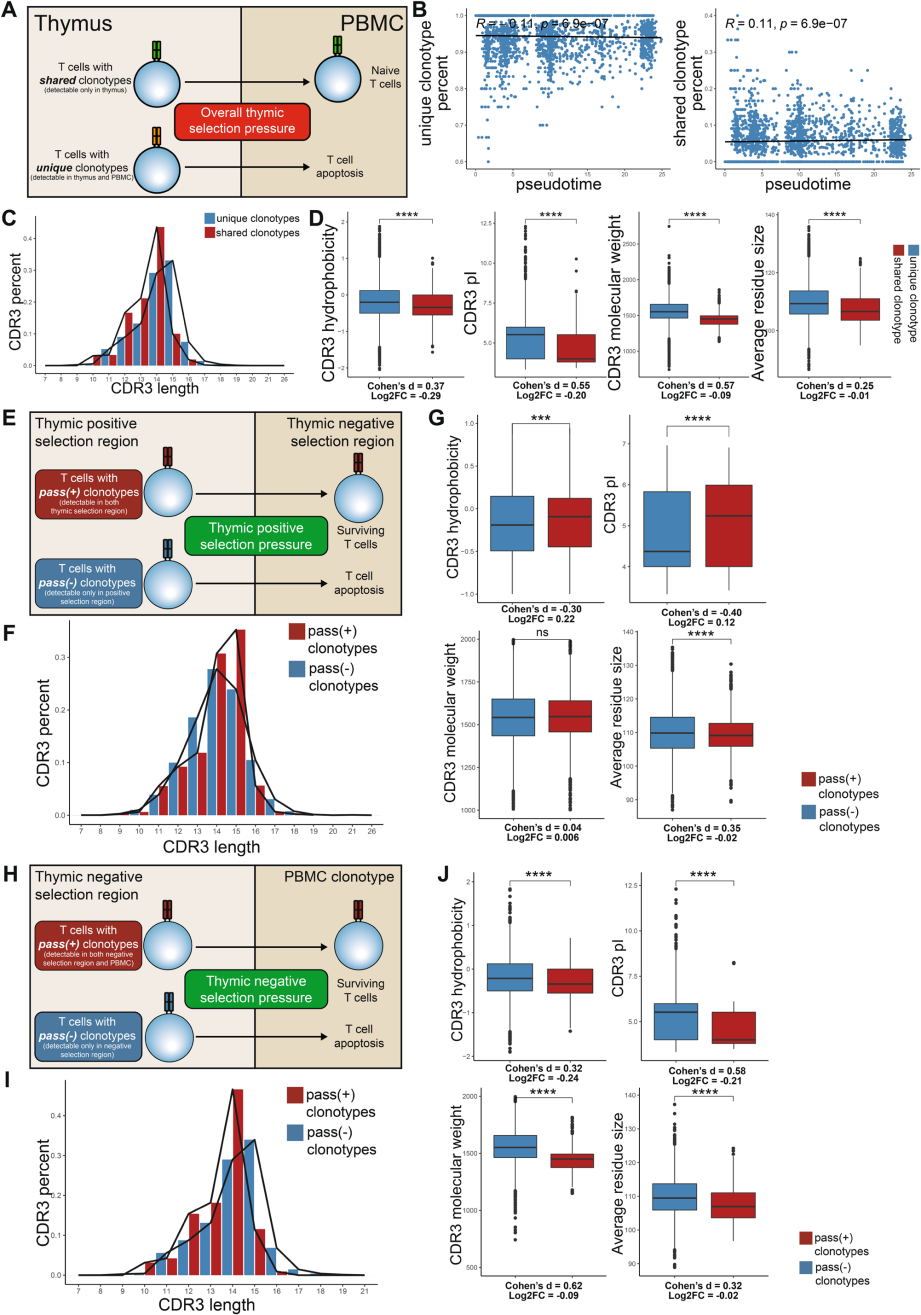
Analyses of the influence of thymic selection on the immune repertoire in the spatial‐TCR‐seq data of 5‐week thymus and PBMC. (A) The schematic diagram of the definition of clonotypes of the TCRβ locus that were unique in the thymus or shared between the thymus and PBMC upon thymic selection by comparison of the immune repertoire from both the thymus sample and the PBMC sample. (B) Scatterplots and correlation of distinct CDR3 types and the pseudo time trajectory. Significances were calculated with the *t*‐test method and correlations were calculated with the spearman method. (C) The bar plot of the proportion of CDR3aa of distinct length grouped by unique clonotypes and shared clonotypes. (D) Box plots of the TRB CDR3aa frequency of distinct physicochemical properties including hydropathicity, pI, molecular weight, and average residue size which were calculated by molecular weight minus CDR3aa length derived from both spatial‐TCR‐seq and ST‐seq data by unique clonotypes and shared clonotypes. A *t*‐test was utilized to calculate the statistical significance (ns: *p* > 0.05; *: *p* < 0.05; **: *p* < 0.01; ***: *p* < 0.001; ****: *p* < 0.0001). Cohen's d and Log2FC were calculated to determine the biological significance. (E) The schematic diagram of the definition of the clonotypes that were detected only in the positive selection region or both in two thymic selection regions by comparison of the immune repertoire from the thymic positive selection region and the negative selection region which were acquired by the K‐means algorithm. (F) The bar plot of the proportion of CDR3aa of distinct length grouped by passed clonotypes and unpassed clonotypes. A *t*‐test was utilized to calculate the statistical significance. (G) Box plots of the TRB CDR3aa frequency of distinct physicochemical properties mentioned above derived from both spatial‐TCR‐seq and ST‐seq data by passed clonotypes and unpassed clonotypes. A *t*‐test was utilized to calculate the statistical significance (ns: *p* > 0.05; *: *p* < 0.05; **: *p* < 0.01; ***: *p* < 0.001; ****: *p* < 0.0001). Cohen's d and Log2FC were calculated to determine the biological significance. (H) The schematic diagram of the definition of the clonotypes that were detected only in the negative selection region or both in the negative selection region and the PBMC sample by comparison of the immune repertoire from the thymic negative selection region and the PBMC sample. (I) The bar plot of the proportion of CDR3aa of distinct length grouped by passed clonotypes and unpassed clonotypes. (J) Box plots of the TRB CDR3aa frequency of distinct physicochemical properties derived from both spatial‐TCR‐seq and ST‐seq data of passed clonotypes and unpassed clonotypes. A *t*‐test was utilized to calculate the statistical significance (ns: *p* > 0.05; *: *p* < 0.05; **: *p* < 0.01; ***: *p* < 0.001; ****: *p* < 0.0001). Cohen's d and Log2FC were calculated to determine the biological significance.

T cells experienced the thymic selection to get the self‐antigen tolerance and MHC restriction. Previous research showed that only a tiny proportion of T cells with proper CDRs (mainly CDR3βs) were mature while others were apoptotic (Nikolich‐Zugich et al. 2004). Thus, the immune repertoire of CDR3β between the thymus and PBMC was compared to identify the clonotypes with a higher likelihood of maturation. About 6% of the immune repertoire of the thymus appeared in the PBMC which was annotated as shared clonotypes and could be mature while the rest were annotated as unique clonotypes and might not be mature (Figure 3A). The frequency correlation of clonotypes, CDR3β amino acid, and VJ gene pairing illustrated that the thymus differed significantly from the PBMC (Figure S22A). The spatial distribution analysis demonstrated that shared clonotypes were enriched in the medulla while the unique clonotypes were enriched in the cortex (Figure S22B). Shared clonotypes were slightly enriched along with the pseudo‐time trajectory (Figure 3B).

Barcodes containing shared clonotypes were annotated as the shared clonotype (+) region. Barcodes only containing unique clonotypes were annotated as the shared clonotype (−) region. The spatial distribution of two sub‐regions (Figure S22C) demonstrated that the shared clonotype (−) region was enriched in the cortex while the shared clonotype (+) region was located both in the cortex and the medulla (Figure S22D). The shared clonotypes (−) region was related to RNA splicing, non‐coding RNA synthesis, TCR recombination, and T cell positive selection while the shared clonotype (+) region was related to T cell negative selection, adaptive immune response, and cytokine‐mediated immunity (Figure S22E–G). The shared clonotype (+) region was at a late trajectory compared to the shared clonotype (−) region (Figure S22H).

**FIGURE 4:**
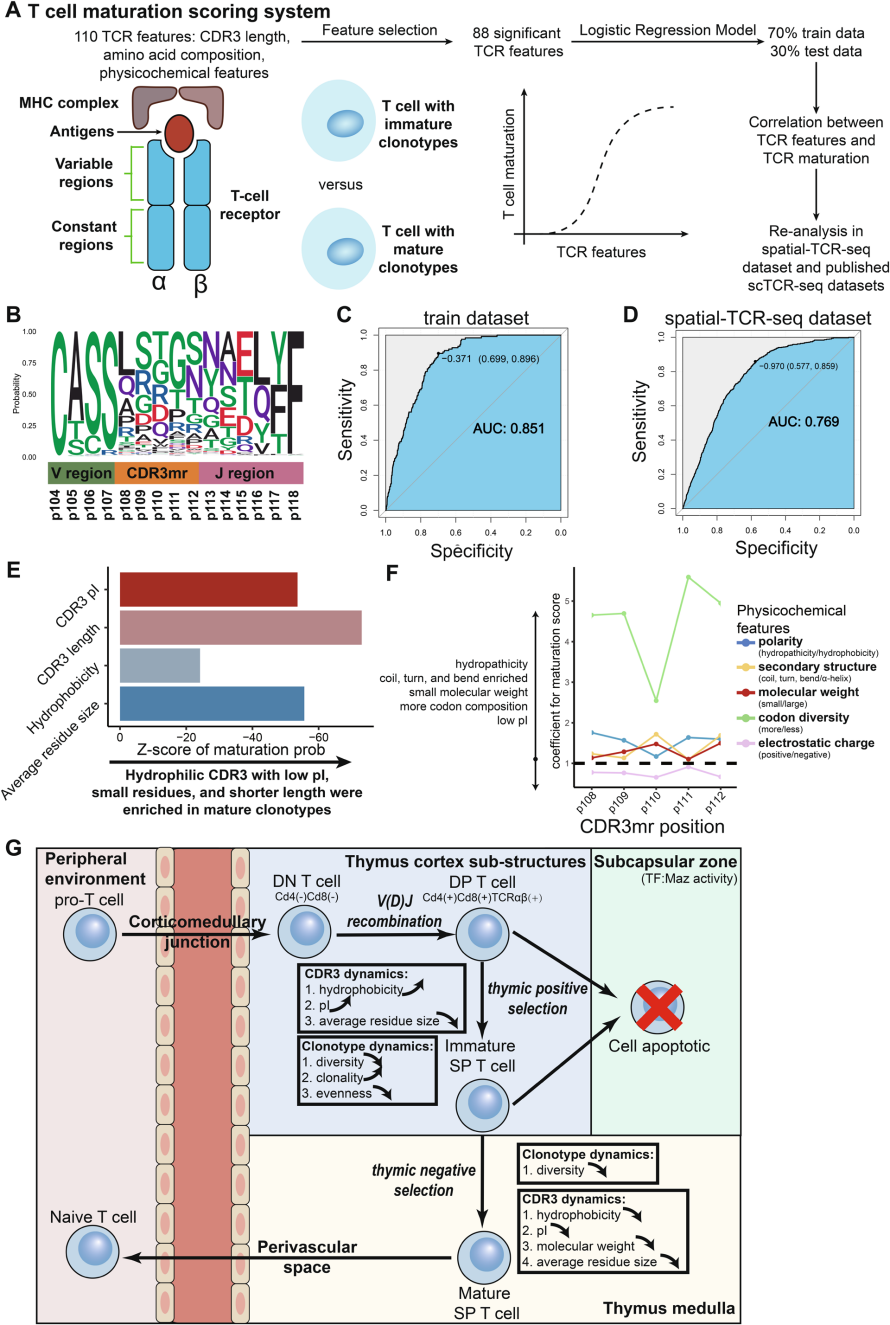
The TCR maturation scoring system showed predicted accuracy of TCR maturation from the thymus in both TCR‐seq and spatial‐TCR‐seq data. (A) The design of the TCR maturation scoring system. (B) Probability of each amino acid in each position depicted by a sequence logo for the most frequent CDR3 length, 15 amino acids. (C) The ROC curve of the training dataset estimates the specificity and sensitivity of the logistic regression model. (D) The ROC curve of the spatial‐TCR‐seq dataset estimates the specificity and sensitivity of the logistic regression model. (E) Bar plots of the scaled correlation between the polarity (pI), CDR3aa length, hydrophobicity, and average residue size with the maturation score, *z*‐score < 0 means hydrophilic and short CDR3aa with negatively charged and small‐size residues were enriched in mature clonotypes. (F) The line plot of the correlation between the maturation score and distinct physicochemical features including polarity, secondary structure, molecular volume, codon diversity, and electrostatic charge of each CDR3mr position. (G) Schematics that illustrate the dynamics of thymic clonotypes during both the thymic positive selection and negative selection.

The CDR3β structure and physicochemical features of distinct clonotypes were investigated. The VJ gene usage and the VJ gene pairing pattern were distinct between shared clonotypes and unique clonotypes (Figure S23A,B). Shared clonotypes were more enriched in several specific VJ genes and pairing patterns, and the whole diversity was significantly decreased (Figure S23C). The diminished proportion of non‐expanded or minimally expanded clones emerged as a pivotal factor driving the reduced clonal diversity within the shared clone populations (Figure S23D). The average length of shared clonotypes was 14 compared to 15 for unique clonotypes (Figure 3C). Shared clonotypes were more enriched in hydrophilic, low pI, small molecular weight, and small average residue size CDR3β (Figure 3D). Two indexes including Cohen's D and Log2FC were calculated to avoid the significance resulted from the large sample size. The amino acid composition analysis also revealed that CDR3βmr (CDR3β middle region, which was shown in Figure 4B) was enriched with glycine and the shared clonotypes showed the tendency to have even more glycine in CDR3βmr (Figure S23E). Besides, shared clonotypes also tended to be enriched in polar amino acids, which could easily form hydrogen bonds (Figure S23F). Shared clonotypes from the scTCR‐seq dataset of both young and aging thymus samples (Figure S24) and published human TCR dataset (Figure S24J) also showed similar results of the CDR3β.

It was already shown that the subcapsular zone was associated with T cell apoptosis while T cells got mature, went through all thymus structures, and were exported to the PBMC. Thus, the comparison of the immune repertoire among sub‐structures was performed to validate intrinsic features of mature clonotypes (Figure S25A). It was revealed that clonotypes only detected in the subcapsular zone showed higher T cell apoptosis (Figure S25B) and their enrichment was positively correlated with T cell apoptosis (Figure S25C). It was also revealed that clonotypes detected in all sub‐structures showed shorter TRB CDR3aa length, lower pI, and lower hydrophobicity (Figure S25D,E). Clonotypes detected in all sub‐structures were expanded in the subcapsular zone and the medulla compared to other regions (Figure S25F).

### 2.10 Thymic Positive and Negative Selection Showed Opposite Selection Pressure on the TCR Immune Repertoire

According to the spatial feature of specific signal pathways expression intensity, barcodes that were enriched in thymic selection processes were sorted out, and the similarities and differences between TCRs detected in distinct regions that tend to pass or not pass the thymic selection were analyzed (Figures 3E,H and S26A).

Clonotypes that were detectable in both selection regions could represent clonotypes capable of passing the positive selection while clonotypes that were only detectable in the positive selection region were supposed to contain clonotypes not capable of passing the positive selection. After comparing the two clonotype groups, the result suggested that certain VJ genes were enriched after the positive selection (Figure S26B). The VJ gene pairing patterns were also less even and dominated by several major pairing patterns after the positive selection (Figure S26C). The distribution analysis of distinct clone sizes suggested that clonal clonotypes were enriched while non‐clonal clonotypes were decreased (Figure S26D). It indicated that certain clonotypes recognized antigens well and proliferated after the positive selection, causing the arising of clonal clonotypes, the decrease in diversity and evenness, and the increase in clonality (Figure S26E).

The CDR3β length was normally distributed before the positive selection but tended to be enriched in several specifically medium lengths after that (Figure 3F). The hydrophobicity of TCRβ CDR3 increased after the positive selection (Figure 3G top left). Interestingly, hydrophobic CDR3 residues have previously been shown to play an important role in the response of autoreactive T cells to their antigens or in the cross‐reaction of multiple antigens (Stadinski et al. 2016; Yin et al. 2011). From the scTCR‐seq data (Figure S27), clonal TCR clonotypes (UMI count > 1) increased while non‐clonal clonotypes (UMI count = 1) decreased in both young and aging samples after the positive selection (Figure S27D). However, clonotypes that passed the positive selection showed higher hydrophobicity in young samples (Figure S27A–E) but showed no significant conclusions in aging samples (Figure S27F–J) which showed the weakened positive selection pressure during aging (Figure 1D).

In previous studies (Takaba and Takayanagi 2017; Lucas et al. 2016), it was demonstrated that T cells migrated from the cortex to the medulla after the positive selection, and it could be assumed that T cell migration could be influenced by a similar pressure like the thymic positive selection. Thus, we compared the TCR clonotypes between these two sub‐structures using the spatial‐TCR‐seq dataset to check the correctness of the conclusion from the above section from distinct perspectives. It was revealed that the migration pressure from the cortex to the medulla was similar to the positive selection pressure (Figure S28).

The immune repertoire between the negative selection region and the PBMC sample was compared to investigate how negative selection influenced immune repertoire (Figure 3H). The analyses of the VJ gene usage and VJ gene pairing pattern showed that the percentage of high‐proportioned VJ genes decreased (Figure S29A,B). The proportion of clonal clonotypes (UMI count > 1) increased after the thymic negative selection (Figure S29C). As a consequence, the diversity decreased while the clonality and evenness did not change significantly (Figure S29D). The average length of clonotypes was 14 after the negative selection compared to 15 before the negative selection (Figure 3I). CDR3βs with low hydrophobicity, low pI, small molecular weight, and small average residue size were preferred after the negative selection (Figure 3J). Similar conclusions were validated in the scTCR‐seq dataset of young and aging thymuses (Figure S30).

### 2.11 The Comparison Between the Thymus and PBMC Using TCR‐Seq and the TCR Maturation Potential Scoring System for the Prediction of TCR Clonotype Maturation

The bulk‐TCR‐seq of the thymus and paired PBMC sample was performed to validate above conclusions. The analyses of the dynamics of the clone sizes demonstrated that the thymuses were more diverse and even but less clonal compared to PBMC samples (Figure S31A). The clonotype clustering analyses utilizing the multi‐dimensional scaling (MDS) method of multiple samples demonstrated that thymus samples were close to each other and distinct from their PBMC samples from the same mouse (Figure S31B). Thymus samples showed similar VJ gene usage and the dominant kinds of VJ genes were persistent in the PBMC samples (Figure S31C). Clonotypes in PBMC were more clonal expanded (Figure S31D). The distribution of the length of TRB CDR3s of PBMC and the thymus was illustrated (Figure S31E). Mature clonotypes which were detected in both samples and immature clonotypes which were detected only in the thymus were acquired by the immune repertoire comparison. The CDR3mr of mature clonotypes was enriched in low pI, small molecular weight, and small average residue size CDR3s (Figure S31F) with hydrophilic residues (Figure S31G), which was consistent with above conclusions.

A scoring system to quantify the TCR‐maturation potential was developed to determine intrinsic features of mature clonotypes undergoing the overall thymic selection pressure (Figure 4A). One hundred and ten TCR features including amino acid composition at each specific position (Figure 4B), physicochemical features, and CDR3 length were collected based on the previous research (Lagattuta et al. 2022). Using a single‐level logistic regression model, the effect of each TCR feature on TCR maturation was quantified and the result was aggregated into a TCR‐maturation score that can be applied to other datasets. The bulk‐TCR‐seq biological replication dataset was utilized to construct the model. 70% of the whole dataset was labeled as the training dataset while the rest of the data and the spatial‐TCR sequencing dataset were utilized as test datasets. The candidate features as fixed‐effects variables including CDR3aa length, global physicochemical features (hydrophobicity, pI, molecular weight, and average residue size), and amino acid composition were utilized to calculate their influence on the dependent variable and those with significance were selected to build the improved model to increase the accuracy of the scoring system. Eighty‐eight significant fixed‐effects variables were utilized to construct an improved scoring system. The sensitivity and specificity of the model were tested with the ROC curve (Figure 4C). The spatial‐TCR‐seq dataset was also used as the independent dataset to test this model and it showed that the model worked well too (Figure 4D). The model was validated using our scTCR‐seq dataset and the published scTCR‐seq data (Domínguez Conde et al. 2022) of both the thymus and the PBMC. The ROC curve and the predicated probability demonstrated that our logistic regression model was also applicable (Figure S31H). We performed more in‐depth interpretation of the logistic model to demonstrate the influence of distinct CDR3aa features on the TCR thymic maturation. According to the influence of each variable on the model, it was suggested that the pI, length, hydrophobicity, and residue size were negatively correlated with the maturation score (*z*‐score < 0; Figure 4E) which corresponded to the result in Figure 3D that hydrophobicity and pI decreased in mature clonotypes. Based on previously published research (Atchley et al. 2005), we further demonstrated the correlation between several factors of individual residues in CDR3mr and TCR thymic maturation. In this analysis, it was also revealed that higher hydropathicity, low pI, and small molecular weight were associated with TCR maturation (Figure 4F). In addition, other factors including the secondary structure and codon diversity of residues were suggested to be associated with the maturation condition (Figure 4F).

## 3 Discussion

Recent ST‐seq studies were able to identify the heterogeneity of tissues in a spatial dimension. The thymus is the central organ where T cells differentiate and mature. The TCR recombination and thymic selection resulted in the diversity of the immune repertoire. The transcriptomic and immunological dynamics of T cells' differentiation during thymic aging haven't been illustrated in the single‐cell resolution. Our research developed spatial‐TCR‐seq and set up the novel combined process for multi‐omics integrating analysis including scRNA‐seq, scTCR‐seq, ST‐seq, spatial‐TCR‐seq, and TCR‐seq to construct the single‐cell and spatially resolved transcriptome and immune repertoire atlas of the mouse thymus during aging.

Using scRNA‐seq and ST‐seq, we constructed the single‐cell and spatially resolved transcriptomics atlas of the thymus which illustrated the cell heterogeneity, the spatially histological structure, and transcriptional feature from both single‐cell and spatial perspective which improved our understanding of the structure and biological functions of the thymus. The scRNA‐seq data demonstrated the cell composition dynamic and the transcriptomic features of T cells and stromal cells during aging. It was demonstrated that proliferating CD69negDN T cells decreased while regulatory CD4SP T cells increased during aging. Our aging‐related findings aligned with established literature while providing spatial resolution that transforms prior observations. We confirmed the age‐dependent increase in regulatory CD4SP T cells reported by Kozlowska et al. (2007), but spatially localize this population to the CMJ and medulla. Similarly, we corroborate the decrease of proliferative CD69negDN T cells described by Li et al. (2024) and identified that the proliferative CD69negDN T cells were located in the cortex_0 niche, where disintegration happened during aging. We proposed that these cellular changes reflect underlying tissue‐level remodeling. Recent evidence demonstrated thymic fibrosis in aging (Li et al. 2024; Rezzani et al. 2014), with fibroblast accumulation. We hypothesized that the cortex_0 vulnerability reflects this microenvironmental deterioration: fibrotic expansion physically compromises the niche architecture required for progenitor maintenance, disrupting chemokine gradients and mechanical support necessary for CD69^−^ DN proliferation. TCR recombination and thymic positive selection were suppressed while T cell apoptosis and thymic negative selection were strengthened during aging.

Utilizing the ST‐seq dataset, the heterogeneity of the cortex was demonstrated and a cortex_0 representing a transcriptionally distinct zone with progenitor‐like signature was identified. This cortex_0 showed distinct transcriptomic features and spatial distribution and was validated in aging sample, distinct sequencing platforms, and human‐derived samples. It was acknowledged that further validations such as genetic perturbation, lineage tracing, and protein level validation were needed to demonstrate the function mechanisms of the cortex_0. Our study was subject to inherent limitations of spatial transcriptomics. Analyses were based on 2D tissue sections, which do not fully capture three‐dimensional thymic architecture; interpretation of spatial relationships should consider this constraint. The use of 12‐month‐old mice represented moderate thymic involution rather than advanced senescence (18–24 months), limiting conclusions regarding terminal aging mechanisms; however, this stage offered specific value for identifying early, potentially reversible changes. Mechanistic exploration of architectural dispersion (e.g., fibrosis‐driven niche destruction) was beyond the scope of this discovery‐phase study and represented important future direction.

Previous research (Heimli et al. 2022) has shown that early thymocytes like DN T cells migrate outwards through the cortex towards the subcapsular zone (Lind et al. 2001) after entering the thymus (Petrie and Zúñiga‐Pflücker 2007) for the expression of Cd4 and Cd8a. DP T cells move back through the cortex towards the medulla and go through the positive selection (Halkias et al. 2013). The spatial migration and differentiation of T cells in the thymus were strictly regulated and thus it could be assumed that the heterogeneity might exist within the cortex. The ST‐seq demonstrated the heterogeneity of the thymus cortex. Three distinct cortexes showed differences in gene expression and spatial distribution.

Using deconvolution, the spatial distribution of T cells and stromal cells was demonstrated. It was demonstrated that CD69negDN T cells were enriched in the Cortex_0 which was associated with the differentiation of progenitor cells. This result was validated by the spatial expression of several signatures including hematopoietic stem cell migration, and differentiation (Ashburner et al. 2000; Aleksander et al. 2023) and marker genes like Cxcr4, Ccr9, and Ccl25 which were associated with the migration of progenitor cells into the thymus (Takahama 2006). Marker genes acquired from previous studies (Park et al. 2020) were utilized to predict the distribution of CD69negDN T cells. The heterogeneity of CD69negDN T cells demonstrated that CD69negDN T cells of early phases were located near the CMJ, while CD69negDN T cells of late phases were enriched in the Cortex_0. Thus, it could be presumed that progenitor T cells instantly enter the thymus from the CMJ but resided in the Cortex_0 for further differentiation. The CMJ was also partially located in the very early pseudo‐time trajectory. It was demonstrated that CD69posDP T cells were enriched in the Cortex_0. Previous studies (Domínguez Conde et al. 2022) and maker genes acquired from the database (Hu et al. 2023) were also utilized to validate the distribution of CD69posDP T cells. The spatial distribution of several vital biological signatures including V(D)J recombination and thymic selection processes illustrated that TCR recombination was enriched in the subcapsular zone, thymic positive selection was enriched in the cortex, and thymic negative selection was enriched in the medulla. The histological distribution of sub‐structures was disrupted in aging thymuses.

The pseudo‐time analyses of scRNA‐seq and ST‐seq datasets systematically revealed the differentiation pathway of T cells and their spatial migration route. In aging samples, the differentiation of CD69neg T cells associated with TCR recombination was weakened while the differentiation of SP T cells associated with thymic negative selection was persistent. It was also suggested that CD69neg T cells that didn't express paired TCR got apoptotic in the subcapsular zone. Furthermore, the transcription factor Maz was identified to be enriched in the subcapsular zone and associated with T cell apoptosis using SCENIC. The ligand & receptor analyses revealed that stromal cells mainly interacted with CD69negDP T cells and NK cells‐mediated MHC‐I signature and B cells‐mediated MHC‐II signatures were significantly promoted in aging samples.

Spatial‐TCR‐seq that combines the TCR with spatial information by specifically amplifying TCR based on the cDNA library has been developed. The location coordinates of each specific clonotype can be determined with this method. Combined with ST‐seq, it helped us to better understand the spatial features of the immune repertoire of several biological processes such as TCR recombination and thymic selection. The differences between immature clonotypes and mature clonotypes helped us to understand how clonotypes and CDR3 are influenced by the thymic selection pressure (Figure 4G). More in detail, the respective features of clonotypes influenced by the positive or the negative selection were compared to illustrate the different dynamics of the immune repertoire during the development of T cells. It was also suggested that mature clonotypes had a V and J gene preference, preferred VJ gene pairing patterns, and a preference for physicochemical features. scTCR‐seq datasets of the thymus during aging were utilized to validate results acquired in the above analyses in the single‐cell resolution and compare differences in the immune repertoire under thymic selection during aging.

Utilizing bulk‐TCR‐seq datasets, a maturation scoring system for T cells in the thymus was constructed. Consistent with the findings in spatial‐TCR‐seq and scTCR‐seq, physicochemical features including pI, hydrophobicity, average residue size, and CDR3 molecular weight are related to the maturation of T cells in the thymus. This scoring system was utilized to predict the auto‐reaction and maturation of TCR clonotypes.

Our study relies on transcriptomic and TCR repertoire analysis, with inherent limitations regarding post‐transcriptional regulation and protein‐level confirmation. We mitigated this through multi‐omics cross‐validation: scRNA‐seq and ST‐seq provide transcriptional context; scTCR‐seq and spatial‐TCR‐seq provide functional readouts (productive TCR rearrangements, clonal migration) validating biological activity beyond mRNA expression; and bulk TCR‐seq provides independent replication. Direct protein validation would strengthen conclusions, but technical constraints limit feasibility: inconsistent antibody specificity for mouse thymus, insufficient IF resolution for cortex_0 boundaries, and lower gene throughput of spatial proteomics. The current study establishes the spatial framework and hypotheses motivating these efforts.

In our research, we performed the spatial‐TCR‐seq of the mouse thymus (the spatial sample). The cDNA library of the same thymus sample was utilized for the bulk‐TCR‐seq to validate the sequencing saturation of the spatial‐TCR‐seq (the bulk_rep sample). We also performed other bulk‐TCR‐seq using five distinct biological thymus samples in the analyses of the logistic regression model (bulk_T1 to T5). To solve the resolution problem of the ST‐seq data, we additionally performed paired scRNA‐seq and scTCR‐seq utilizing two distinct biological thymus samples (sc_rep_1 and sc_rep_2). To quantify reproducibility, we compared TCR clonotypes obtained with the four platforms represented in our dataset: spatial‐TCR‐seq, bulk‐TCR‐seq (five biological replicates), bulk‐TCR‐seq replicate of the spatial section (bulk_rep), and scTCR‐seq (two biological replicates). Overlapping clonotypes were first assessed with the jaccard index (Figure S32A). Spatial‐TCR‐seq shared the majority of its clonotypes with the matched bulk_rep sample (overlap ≈90%), confirming faithful detection of the tissue‐resident repertoire. In contrast, clonotypes among the five independent bulk‐TCR‐seq replicates exhibited limited mutual overlap (mean 26%–32%), reflecting expected biological heterogeneity between mice. Similarly, the two scTCR‐seq replicates displayed moderate overlap (≈4%), consistent with sampling noise inherent to low‐input single‐cell protocols. We further compared the proportion of VJ genes usage (Figure S32B–E) and VJ genes pairing pattern (Figure S32F,G). The spatial‐TCR‐seq and its bulk‐TCR replication showed similar VJ genes usage and pairing patterns. The VJ proportion correlation and clonotypes overlap were separately analyzed utilizing TCR clonotypes in distinct sequencing platforms (Figures S33 and S34).

Thus, through integrated scRNA‐seq, ST‐seq, Spatial TCR‐seq, scTCR‐seq, and TCR‐seq investigation, the network including spatial features, gene expression profile, and immune repertoire in the thymus was profoundly illustrated and could be very helpful for further investigating the link of thymus and various disease susceptibility. This integrated method could also be a powerful means for other organs' investigation in the future.

## 4 Methods

### 4.1 Sample and Design

#### 4.1.1 Mice

Five‐week‐old and 12‐month‐old C57BL/6 female mice were purchased from Beijing Vital River Laboratory Animal Technology Co. Ltd. (Beijing, China). All mice were maintained in the specific pathogen‐free animal facility at Wuhan University and all animal experiments were approved by the Institutional Animal Care and Use Committee of Wuhan University.

#### 4.1.2 Thymus and PBMC Acquisition

The thymus was obtained immediately after the execution of the mice. The whole thymus was washed with PBS and put in a 1.5 mL EP tube filled with RNA‐BE‐LOCKER at −20°C overnight and transferred to −80°C. The production was used for RNA extraction and TCR‐seq. The whole blood was obtained by collecting the mouse eyeball and transferring it to the EP tube. The PBMC was collected by the protocol of mouse lymphocyte separation medium. The whole blood was diluted with PBS and softly flowed into the mouse lymphocyte separation medium to keep the edge clear. Then the PBMC was separated by density gradient centrifugation at 800 *g* for 25 min, with slow increase speed and slow decrease speed. The cell at the intermediate level was isolated and washed with PBS three times. If red blood cells remain in the final production, the erythrocyte lysate was used. The collection was stored in Trizol at −80°C for RNA extraction and TCR‐seq.

#### 4.1.3 RNA Extraction for TCR‐Seq

The RNA of the paired thymus sample and PBMC sample was extracted based on the Trizol protocol and stored in DEPC water for further experiments. The TCR cDNA libraries were generated based on the SMART‐seq2 protocol. The RNA was first reversely transcribed to the cDNA first chain and was amplified with TCR‐specific primers. UMI containing 10 random nucleotides was added to the end of the cDNA library to eliminate the PCR bias. Finally, the sequence adaptors were ligated to the ends. The cDNA libraries were sequenced with the Illumina sequencing platform. The TCR information was extracted from the original sequencing files using TRUST4 to eliminate the PCR bias and transferred to further analyses. The TCR‐seq of the mouse thymus and corresponding PBMC were done as the supplementary material of the immune repertoire analyses in spatial transcriptomics and spatial immune repertoire analyses. The intrinsic characteristics of these samples were analyzed. The comparison between paired thymus and PBMC was done to analyze the differences and similarities. The immune repertoire data was utilized as the training data to build a logistic regression model to predict the maturation score of clonotypes in the thymus.

### 4.2 10× Genomics Spatial Transcriptomics Sequencing

#### 4.2.1 Sample Preparation

An isopentane and liquid nitrogen bath was prepared at least 10 min before freezing samples. The fresh thymus sample was transferred into isopentane using forceps. Once frozen, the sample was transferred to a pre‐chilled container for storage. Next, the sample was embedded in OCT after freezing. The bottom of the cryomold was filled with pre‐cooled OCT, the sample was put on the surface and any exposed area was covered with OCT. The sample was transported to −80°C for storage once the OCT was completely opaque. For cryosectioning, the OCT embedded sample was removed from the −80°C and cryosectioned in a cryostat to generate sized sections for Visium Spatial slides while keeping the sample frozen. The thymus sample was placed within the frames of capture areas on Visium Spatial slides. For the Visium Spatial Tissue Optimization Slide, 7 of the 8 Capture Areas are used for tissue and one is left empty for a positive RNA control. Only one tissue type and section thickness should be tested per slide. It's recommended to assess the RNA quality of the sample block by calculating the RNA Integrity Number (RIN) of freshly collected tissue sections. RIN should be ≥ 7. Large tissue should be scored during sectioning to generate smaller samples to fit the Capture Areas.

#### 4.2.2 Fixation, Staining, Imaging, and Construction of the cDNA Library

The thymus samples were processed according to the Visium Spatial Gene Expression User Guide (10× Genomics), and all reagents were from the Visium Spatial Gene Expression Kit (10× Genomics). First, sections were fixed in chilled methanal for 30 min at −20°C, stained with hematoxylin and eosin, and mounted in 85% glycerol for imaging, which was performed on a whole‐slide scanner at 40× magnification. After imaging, sections were permeabilized at 37°C for 45 min; the on‐slide reverse transcription reaction was performed at 53°C for 2 h. Permeabilization time and RT reaction length were determined using the Visium Spatial Tissue Optimization Kit (10× Genomics). Second‐strand synthesis was subsequently performed on the slide for 15 min at 65°C. All on‐slide reactions were performed in a thermocycler with a metal slide adapter plate. After that, samples were transferred to tubes for cDNA amplification and cleanup.

#### 4.2.3 Sequencing

10× Genomics Visium libraries were pooled, denatured, and diluted to a loading concentration of 1.8 pM with 1% PhiX control, followed by paired‐end sequencing on an Illumina NextSeq 500 to a depth of approximately 110–180 million paired reads per sample. Sequencing data were processed using the Space Ranger pipeline v.1.0.0 (10× Genomics).

### 4.3 Spatial Immune Repertoire Sequencing

#### 4.3.1 Generation of the Spatial Immune Repertoire Library

To generate a cDNA library that includes the CDR3 region of the TRV gene as well as the UMI and spatial barcodes indicating the spatial information, TCR must be specifically amplified to increase the proportion in the whole cDNA library. One primer was designed based on the TruSeq Read 1 sequence on sequence 3′. The other primer was designed based on the TRV gene on sequence 5′. Nested PCR reactions were done for the enrichment of the TCR sequence by extending the primers on sequence 5′. Each TRV gene was amplified separately and a pool of separate cDNA libraries was mixed to become a final cDNA library for sequencing. Such a TCR‐specific cDNA library amplified with these primers contains both spatial information and immune repertoire information. The sequences of primers were presented in Tables S1 and S2.

#### 4.3.2 The Recovery of the TCR and Spatial Information With TRUST4

The read1 original file sequencing the 3′ of the TCR cDNA library contains spatial barcodes and UMI information and the read2 file sequencing the 5′ of the TCR cDNA library contains the immune repertoire information including CDR3aa, V gene, and J gene. In the read1 original file, the 16 nt spatial barcodes were identified, and any base before the spatial barcode was abandoned to make sure every sequence read starts with spatial barcodes and UMI. The 16 nt length barcode and 12 nt length UMI were combined as the new barcode to eliminate the bias in different degrees of amplification. Every sequence read containing the spatial barcode was extracted and formed a new file, and the following UMI was used to count the original reads as well as eliminate the PCR bias. The corresponding sequencing reads were also extracted from the read2 file.

TCR Receptor Utilities for Solid Tissue (TRUST) is a computational tool to analyze TCR and BCR sequences. TRUST4 supports both single‐end and paired‐end sequencing data with any read length. Two files from the previous analysis were used as the input of the TRUST4 pipeline. TRUST4 performs de novo assembly on V, J, and C genes including the hypervariable complementarity‐determining region 3 (CDR3), and reports the consensus of BCR/TCR sequences. TRUST4 then realigns the contigs to IMGT reference gene sequences to report the corresponding information. The first 28 nt in the read1 file were labeled as barcodes and the rest of the sequence reads in the two files were labeled as TCR sequences. TCR reads were aligned to the GRCm38 reference genome and consensus TCR annotation which were acquired from IMTG.

## Author Contributions

Conceptualization: L.Y. Methodology: J.F., J.L., P.C., L.Y. Investigation: J.F., J.L., P.C., L.Y., Z.S., B.H., Y.W., Y.C. Visualization: J.F., J.L., P.C. Funding acquisition: L.Y., Y.C., J.L., P.W. Project administration: J.F., J.L., P.C. Supervision: L.Y. Writing – original draft: J.F., L.Y. Writing – review and editing: J.F., L.Y., J.L., P.C., Y.C., N.W., Y.H., P.W.

## Funding

This work was supported by the National Key R&D Program of China (2022YFA1303500 to L.Y.), the International Joint Project (NSFC‐GF) on Diagnostic Technology (82561128251/2025DNJP0203 to L.Y.), National Natural Science Foundation of China (32371271 to L.Y., 32171210 to L.Y., 32301017 to J.L., 31870728 to P.W.), Science and Technology Foundation of Wuhan (KYXM2022003 to L.Y.), State Key Laboratory of Metabolic Dysregulation & Prevention and Treatment of Esophageal Cancer (to Y.C.) and The Construction Fund of Key Medical Disciplines of Hangzhou (2025HZZD13 and 2025HZGF09 to J.L.).

## Ethics Statement

The authors have nothing to report.

## Consent

The authors have nothing to report.

## Conflicts of Interest

The authors declare no conflicts of interest.

## Supporting information


**Figure S1:** Quality control of the ST‐seq data and scRNA‐seq data of thymus samples.
**Figure S2:** Analyses of distinct cell populations of the thymus during aging of the scRNA‐seq data.
**Figure S3:** Analyses of distinct sub‐structures of the thymus ST‐seq data.
**Figure S4:** Validation of transcriptomic features and spatial distribution of the Cortex_0 in the SeekSpace single‐cell spatial transcriptomics dataset.
**Figure S5:** Validation of transcriptomic features and spatial distribution of the Cortex_0 in the 10× Visium human spatial transcriptomics dataset.
**Figure S6:** The predicted distribution of cell subsets in ST‐seq datasets using the CARD deconvolution pipeline.
**Figure S7:** Analyses of T cell lineage of the thymus during aging in scRNA‐seq libraries.
**Figure S8:** The predicted distribution of T cell subsets in ST‐seq datasets using the CARD deconvolution pipeline.
**Figure S9:** The validation of the spatial distribution prediction of CD69negDN T cells and CD69posDP T cells utilizing published datasets and databases.
**Figure S10:** The heterogeneity of CD69negDN T cells demonstrated the differentiation and migration in the thymus.
**Figure S11:** The single‐cell pseudo‐time trajectory of T cells during aging in the scRNA‐seq data.
**Figure S12:** The spatial pseudo‐time trajectory of the thymus in the ST‐seq data.
**Figure S13:** Transcription factor Maz mediated thymocyte apoptotic in subcapsular zone.
**Figure S14:** Increased interactions among B cells, NK cells, and CD69negDP T cells mediated by MHC signature during aging in the scRNA‐seq dataset.
**Figure S15:** Quality control of the spatial‐TCR‐seq data and the scTCR‐seq.
**Figure S16:** The PCA reduction of the proportion of distinct TCR VJ genes, CDR3, and lengths.
**Figure S17:** The PCA reduction of the proportion of distinct TCR VJ genes, CDR3, and lengths.
**Figure S18:** Analyses of the overall spatial distribution of clonotypes.
**Figure S19:** The difference in TCR immune repertoire in distinct samples of distinct ages in the scTCR‐seq data.
**Figure S20:** The difference in the TCR immune repertoire of T cell subsets in the scTCR‐seq data.
**Figure S21:** Spatial, single‐cell, and transcriptomic features of the TCR pairing process in the spatial‐TCR‐seq data and scTCR‐seq data.
**Figure S22:** The transcriptional and spatial features of the thymic TCR maturation process in the spatial‐TCR‐seq data.
**Figure S23:** Analyses of the immune repertoire of the thymic TCR maturation process in the spatial‐TCR‐seq data.
**Figure S24:** Analyses of the immune repertoire of the thymic TCR maturation process in the scTCR‐seq data.
**Figure S25:** Differences of mature clonotypes and apoptotic clonotypes revealed by the comparison of the immune repertoire among thymus sub‐structures in the ST‐seq dataset.
**Figure S26:** The overall features of the dynamic of TRB VJ gene usage upon the thymic positive selection in the spatial‐TCR‐seq data.
**Figure S27:** The overall features of the dynamic of the immune repertoire upon the thymic positive selection in the scTCR‐seq data.
**Figure S28:** The difference in TCR immune repertoire between the cortex and the medulla in the spatial‐TCR‐seq data.
**Figure S29:** The overall features of the dynamic of TRB VJ gene usage upon the thymic negative selection in the spatial‐TCR‐seq data.
**Figure S30:** The overall features of the dynamic of the immune repertoire upon the thymic negative selection in the scTCR‐seq data.
**Figure S31:** The comparative analyses of the thymus and the PBMC of the TCR‐seq data.
**Figure S32:** The comparative analyses of TCR clonotypes among distinct samples and distinct sequencing platforms.
**Figure S33:** Comparative analysis of TCR clonotypes among spatial‐TCR‐seq, matched bulk‐TCR‐seq replicate, and two scTCR‐seq biological replicates.
**Figure S34:** Comparative analysis of TCR clonotypes among spatial‐TCR‐seq, matched bulk‐TCR‐seq replicate, and two scTCR‐seq biological replicates.
**Table S1:** primers for TCR‐specific amplification (step 1).
**Table S2:** primers for nested amplification (step 2).

## Data Availability

All relevant data can be required under reasonable requests.
