# Supplementary material for: Single Cell and Spatially Resolved Transcriptome and Immune Repertoire of Mouse Thymus During Aging Reveal Immunological Heterogeneity and Direction of Thymic Selection Pressure
Source: Aging Cell. 2026 Jul 25;25(8):e70631. doi: 10.1111/acel.70631 (PMC13401621; doi:10.1111/acel.70631)
Supplement: Supplementary file 1 — Figure S1: Quality control of the ST‐seq data and scRNA‐seq data of thymus samples. Figure S2: Analyses of distinct cell populations of the thymus during aging of the scRNA‐seq data. Figure S3: Analyses of distinct sub‐structures of the thymus ST‐seq data. Figure S4: Validation of transcriptomic features and spatial distribution of the Cortex_0 in the SeekSpace single‐cell spatial transcriptomics dataset. Figure S5: Validation of transcriptomic features and spatial distribution of the Cortex_0 in the 10× Visium human spatial transcriptomics dataset. Figure S6: The predicted distribution of cell subsets in ST‐seq datasets using the CARD deconvolution pipeline. Figure S7: Analyses of T cell lineage of the thymus during aging in scRNA‐seq libraries. Figure S8: The predicted distribution of T cell subsets in ST‐seq datasets using the CARD deconvolution pipeline. Figure S9: The validation of the spatial distribution prediction of CD69negDN T cells and CD69posDP T cells utilizing published datasets and databases. Figure S10: The heterogeneity of CD69negDN T cells demonstrated the differentiation and migration in the thymus. Figure S11: The single‐cell pseudo‐time trajectory of T cells during aging in the scRNA‐seq data. Figure S12: The spatial pseudo‐time trajectory of the thymus in the ST‐seq data. Figure S13: Transcription factor Maz mediated thymocyte apoptotic in subcapsular zone. Figure S14: Increased interactions among B cells, NK cells, and CD69negDP T cells mediated by MHC signature during aging in the scRNA‐seq dataset. Figure S15: Quality control of the spatial‐TCR‐seq data and the scTCR‐seq. Figure S16: The PCA reduction of the proportion of distinct TCR VJ genes, CDR3, and lengths. Figure S17: The PCA reduction of the proportion of distinct TCR VJ genes, CDR3, and lengths. Figure S18: Analyses of the overall spatial distribution of clonotypes. Figure S19: The difference in TCR immune repertoire in distinct samples of distinct ages in the scTCR‐seq data. Figure [file ACEL-25-e70631-s001.docx]

# Supplementary Materials and Methods

# Spatial transcriptomics (SeekSpace)

Single-cell nuclei suspension with spatial barcodes was prepared from fresh-frozen tissues using the SeekSpace® Single Cell Spatial Transcriptome-seq Kit (K02501-08). Fresh-frozen tissues were cryo-sectioned to 10-20 μm on a cryostat (Leica) at -20 °C. The tissue regions of interest were carefully placed on the SeekSpace® Chip, ensuring no folds were present. A finger was pressed on the back of the chip to melt the tissue. The SeekSpace® Chip was then placed in the SeekSpace® sc-Spatial Chip Holder and incubated on a Thermocycler Adaptor at 37°C for 90 seconds. Then, a Space Chamber was placed on the chip and 150 µL of labeling reagent was added without introducing bubbles. Next, the tissue sections were fixed, fluorescence photographed, and homogenized in pre-chilled lysis buffer using a Dounce homogenizer (KIMBLE #D8938). After washing and filtration, the number of nuclei was estimated using a Fluorescence Cell Analyzer (Seekgene#M002B or Countstar® Rigel S2) with AO/PI reagent, before being placed on ice for further use. The single-cell RNA-Seq library and spatial barcode library were prepared using the SeekSpace® Single Cell Spatial Transcriptome-seq Kit (K02501-08) according to the manufacturer's instructions. The libraries were then sequenced on the Illumina NovaSeq 6000 with a paired-end 150 bp read length. The analyses process of the SeekSpace ST-seq datasets were similar to the 10X Genomics platform.

# scRNA-seq and scTCR-seq sequencing

## Thymus single-cell isolation

Thymus from 5 weeks old and 12 months old mice were placed into cold 1× PBS with 2% fetal bovine serum (FBS). Samples were chopped into small pieces and disintegrated using enzymatic digestion in a 37 °C water bath for 30–40 min, using 0.5 mg/ml collagenase D (Roche, 1088858), 10 mg/ml dispase II (Roche, 04942078001) and 100 μg/ml DNase II (Sigma-Aldrich, DN25) in RPMI with 2% FBS. Cells were filtered with a 50-μm mesh filter and washed with 7.5 ml MACS buffer (1× PBS with 5 mM EDTA and 2% FBS). Cells were centrifuged at 230g for 4 min. Percoll gradient density centrifugation was performed to enrich the T cell compartment. T cells were resuspended in 2 ml of 1.115 g/ml isotonic Percoll (Sigma-Aldrich, P1644) and placed at the bottom of a tube. Subsequently, 1 ml of isotonic 1.065 g/ml Percoll and then 1 ml of 1× PBS were layered on top. The Percoll gradient was centrifuged at 2,700 rpm., 4 °C, with no deceleration for 30 min. T cells that accumulated between the top and middle layers were collected and washed with MACS buffer and centrifuged at 230g for 4 min for single-cell library construction.

## Mozhuo single-cell library preparation and sequencing

After the digestion of the thymus sample, the 5’ mRNA single-cell method was utilized. Cells were loaded on the Mozhuo platform to capture single cells following the manufacturer’s protocol. Generation of gel beads in emulsion (GEMs), barcoding, and GEM reverse transcription was performed using the Chromium Single Cell 5’ (user guide, no. S060200201) and Chromium Single Cell V(D)J Reagent Kits (user guide, no. S060420201) according to manufacturer’s instructions. Full-length, barcoded cDNA was amplified by PCR to generate enough mass for library construction (Nextera® PCR primers) (Illumina, CA, USA). Full-length TCR V(D)J segments were enriched from amplified cDNA from the 5’ library via PCR amplification by TCR-specific primers using a Chromium Single Cell V(D)J Reagent Kits (user guide, no. S060420201) according to the manufacturer’s protocol. Sequencing of the libraries was performed on HiSeq2500 (Illumina, CA, USA).

# Data processing

## Processing of transcriptomics data

### Data preprocessing and quality control

The initial preprocessing steps that we performed on the spot by gene expression data are similar to a typical single-cell RNA-seq experiment. The first step is quality control, barcodes whose percent of mitochondrial genes and ribosomal genes were too high need to be abandoned. Barcodes whose unique gene count is less than 200 or more than 2500 also need to be abandoned. To mitigate the effects of cell cycle heterogeneity in datasets, we calculated cell cycle phase scores based on canonical markers and regressed these out of the data during pre-processing. Then the data need to be normalized first to account for variance in sequencing depth across data points. The variance in molecular counts/spots can be substantial for spatial datasets if there are differences in cell density across the thymus sample. The substantial heterogeneity requires effective normalization. As for the spatial transcriptomics sample, the SCTransform was recommended to build regularized negative binomial models of gene expression to account for technical artifacts and preserve biological variance.

### Dimensionality reduction and clustering

The data was processed by R version 4.0.0, R package Seurat version 3.1.4, and other related R packages. The data was scaled with all the genes. Then we found the top 2000 variable genes which were used for the following dimensionality reductions. The principal component analysis reduction and UMAP reduction were done with default parameters. Clusters identification was done with the FindCluster function in the Seurat R package with a resolution of 1.6 and the first 50 PCs. The further annotation of the sub-structures was decided by plotting clusters onto the associated histology images and tested with specific signature expression levels based on formerly known knowledge.

### Cell type annotation of scRNA-seq data and cell type prediction of ST-seq data

In the scRNA-seq data, the cell type of each cluster after the reduction was annotated based on marker genes of distinct cell types. Clusters highly expressing Cd19, Cd79a, and Cd79b were inferred to be B cells. Clusters highly expressing Cd74, H2-Eb1, and H2-Ab1 were inferred to be dendritic cells. Clusters highly expressing Gsn, Col5a2, and Col3a1 were inferred to be fibroblasts. Clusters highly expressing Adgre1, Mrc1, and Cd68 were inferred to be macrophages. Clusters highly expressing Kit were inferred to be mast cells. Clusters highly expressing Klrb1c and Nkg7 were inferred to be natural killer cells. Clusters highly expressing Cd3e, Cd3d, and Cd3g were inferred to be T cells. Clusters highly expressing Epcam, Cd1d1, and Cd44 were inferred to be thymic epithelial cells.

Each spot from the spatial visium assay might contain the expression file of multiple cells at a 50um resolution. The cell type percent of barcodes can be predicted with the thymus scRNA-seq dataset based on the deconvolution algorithm CARD^1^. The single-cell RNA-seq-derived annotation labels can be transferred to the spatial transcriptomics dataset and the probabilistic classification scores and predictions were set as a new assay in the thymus Seurat object^2-5^. The clustering and annotation of T cell subsets were analyzed in a similar pipeline.

### Gene expression analysis and signaling pathway analyses

Differential gene expression of cell populations in scRNA-seq data, as well as sub-structures in ST-seq data with biological significances, was calculated via the FindMarkers function of the Seurat package. Differential genes-enriched signatures were calculated with clusterProfiler, org.Mm.eg.db, and AnnotationHub R packages. The expression level of interesting spatial signaling pathways was also calculated by the AddModuleScore function of the Seurat package to imply restricted distribution or expression patterns of a specific gene set obtained from the Gene ontology database^6^.

### Spatial transcriptomics spot distance-based analyses

Since the result for Cell Ranger (10X genomics) contains the spatial coordinates of each barcode, the Euclidean geometric distance could be calculated^7^. The shortest distance was implied by calculating the shortest distance between each barcode of a specific type and the barcodes of another type. The aggregate of the shortest distances was used to directly imply the spatial distribution pattern between cell types or sub-structures. The aggregate of the shortest distance to the border of the thymus sample implied the tissue depth of a single region or cell type. We calculated the proportion of the nearest 6 barcodes around a specific barcode to illustrate the spatial interaction among sub-structures.

## Processing of trajectory data

The pseudo-time trajectory of the thymus was analyzed using the R package monocle and Biobase. The pseudo-time trajectory of the scRNA-seq dataset was performed with the default pipeline using the Monocle3^8^ package. The distribution of T cell subsets and the correlation between signatures and pseudo-time were analyzed. For the thymus ST-seq dataset, the whole tissue’s trajectories mainly focus on the development trajectories of sub-structures. The gene set used for establishing the trajectories was from the top 2000 variable genes whose p-value < 0.05. The root of the trajectory was decided based on previous knowledge. With the result from pseudo-time analysis, the differential gene expression and spatial pseudo-time analysis can be done to imply the specific biological progress and the spatial distribution of thymocytes’ development. The Slingshot^9^ analyses were performed using default parameters and the Cortex_0 was selected as the route.

## Processing of transcription factor data

The prediction of transcription factors (TFs) of distinct sub-structures in the thymus ST-seq dataset was performed by the SCENIC^10^, GENIE3, Rcis Target, and AUCell packages. The expression matrix was utilized as the input material. The first step was building the gene regulatory network which identified potential targets for each TF based on co-expression, and selected potential direct-binding targets based on DNA-motif analyses (Rcis Target). The second step was identifying cell states and their regulators which analyzed the network activity in each barcode (AUCell) and identified stable barcode states based on their gene regulatory network activity. The activity and openness level of TFs were included in the results. We calculated the correlation between the activity & openness of TFs and the pseudo-time trajectory to reveal their dynamics during the differentiation. We also calculated the correlation between the activity & openness of TFs and several signatures. At last, we calculated the activity heterogeneity of TFs in distinct spatial sub-structures and the correlation of TFs activity levels.

## Processing of the single-cell receptor & ligand analyses

We analyzed the differences in receptor and ligand communication between scRNA-seq samples with R packages including CellChat^11^, NMF, and ggalluvial. The cellchat object was constructed by importing the gene expression matrix and the annotation of cell types. We import the mouse receptor & ligand database as the reference. The signal pathway-related genes were extracted from the matrix, and the receptors and ligands that were highly expressed in each subgroup were searched which were projected onto the protein-protein interaction network. If the ligand or receptor is overexpressed, the interaction between the overexpressed ligand and receptor is identified. By inferring the ligand-receptor communication network, the aggregated communication network between subgroups is calculated based on the number of links and summarizing the total communication probability.

## Processing of immune repertoire data

### Quality control of the spatial immune repertoire data

We compared barcodes detecting the variable region of TCRs in the spatial immune repertoire data and the constant region of TCRs in the ST-seq assay. We also calculated the correlation between the variable region counts and the constant region transcript counts as well as marker genes. We calculated the percent of UMIs that only detected one variable region of TCRs. For the scTCR-seq data, we calculated the percent of T cell barcodes that detected paired TCR reads.

### Space-based immune repertoire analysis

Since every single clonotype corresponds to a specific spatial barcode, the spatial distribution of the clonotype can be determined. A specific barcode contains multiple cells and multiple clonotypes because of the resolution of spatial transcriptomics of the 10X genomics platform. Every barcode was replaced by a specific pile chart which tells the proportion of each clonotype within the barcode. The pile chart was placed on the specific coordinate corresponding to the barcode. This method helps us to get the spatial distribution pattern of any single clonotype which also applies to the CDR3 amino acid sequence, TRV gene, TRJ gene, and CDR3aa length. Based on the composition of clonotypes of each barcode, we performed the PCA reduction to imply the dispersion of the immune repertoire in each sample. The diversity and clonality of each barcode were calculated with the CalcDiversityStats function of the Vdjtools software^12^. The diversity index was determined by calculating the logarithm of Shannon entropy. The richness index was assessed based on the count of unique clonotype types. The clonality index was derived by comparing the diversity index to the richness index. The physicochemical features including hydropathicity, pI, and molecular weight of CDR3 of distinct tissue regions were extracted from the ExPASy Compute Pi/Mw websites (http://web.expasy.org/compute_pi/)^13^.

### TCR chain-pair analysis

We analyzed the spatial distribution of pre-TCRα, TCRα chain, and TCRβ chain. Barcodes expressing Ptcra were annotated as Ptcra (+) region. Barcodes not expressing Ptcra were annotated as Ptcra (-) region. The spatial distribution of distinct TCR chains and distinct tissue regions. The transcriptional profile of each cluster and the correlation between TCR and pseudo time order were analyzed. We analyzed the expression of Ptcra in T cell subsets in the scTCR-seq data.

### The influence of the thymic selection process on the immune repertoire

The thymus is the organ where thymic positive selection and negative selection happen. T cells that experienced the selection become mature and exported to the peripheral environment. Analyzing the clonotype appearance both in the thymus and PBMC (peripheral blood mononuclear cell) helped us to identify the mature clonotypes as well as immature clonotypes. The annotation of the TCR maturation condition is vital for further analysis including spatial distribution analysis and differential gene expression profile which help us to understand how thymic selection pressure generally influences thymic immune repertoire. Thymic positive selection region and negative selection region were acquired by the K-means algorithm. Clonotypes that appeared both in two regions were annotated as the clonotypes that were preferred to pass the positive selection. Clonotypes that appeared both in the negative selection region and PBMC sample were annotated as the clonotypes that were preferred to get through the negative selection and get mature. The scRNA-seq data and scTCR-seq data were utilized to improve the resolution of the above conclusions by similar methods and analyses. Two effect-size metrics: Cohen’s d and Log2FC were utilizing to validate the significance resulted from the large sample size of the spatial-TCR-seq dataset. Cohen’s d – the standardized mean difference: d = (μ₁ – μ₂) / σ_pooled, where σ_pooled = √[((n₁ – 1)σ₁² + (n₂ – 1)σ₂²) / (n₁ + n₂ – 2)]. |d| ≥ 0.2 was taken as the smallest biologically relevant threshold (small effect). Log2 fold-change (Log2FC) – the log2-ratio of group means: Log2FC = log2(mean₁ + 1) – log2(mean₂ + 1). |Log2FC| ≥ 0.2 (∼15 % linear change) was set as the minimal meaningful cutoff. Only comparisons satisfying |Cohen’s d| ≥ 0.2 **or** |Log2FC| ≥ 0.2 were classified as biologically significant.

### Immune repertoire analysis of multiple sub-structures

Since the TRUST4 results included the immune repertoire information corresponding to the spatial information, the whole immune repertoire data can be divided into multiple sub-immune repertoires. The TCR VJ gene usage, pairing proportion, CDR3aa bias, and clone size were calculated among multiple tissue regions. CDR3aa amino acid sequence bias analysis was done with the ggseqlogo R package. The frequency of residues in each specific position was calculated with the TCR-intrinsic regulatory potential (TiRP)^14^. The physicochemical features of amino acids were obtained from the Atchley factor of each kind of amino acid^15,16^.

### Processing of TCR-seq data for the comparison and the scoring system

The TCR-seq of paired thymus and PBMC samples were utilized as the supplementary material of ST-seq to compare the differences between the thymus and the PBMC sample and for the construction of the TCR-maturation potential scoring system to predict the maturation of clonotypes in the thymus. The mature clonotypes and immature clonotypes were acquired by comparison between the thymus and the PBMC. Several immune repertoire features including diversity, clonality, and evenness were calculated using VDJtools. Intrinsic features including CDR3 length, gene usage, clone size, and residue composition were extracted from the clonotypes. Physicochemical features including polarity, secondary structure, molecular weight, codon diversity, and electrostatic charge were acquired from previous research^15,16^ and the website.

Mature clonotypes and immature clonotypes were combined as the input material. To eliminate the error caused by the small proportion of positive control as well as to preserve the integrity of the whole sample, the mature clonotypes were replicated properly. We performed this analysis based on a previously published research^14^. A single-level logistic regression model (generalized linear model, GLM) was fitted to predict binary TCR maturation status (mature:1; immature:0) as the dependent variable. No mixed-effects structure (i.e., no random intercepts or slopes) was ultimately adopted, because the dataset was curated to ensure independence of observations (one maturation status per TCR sequence). Fixed-effects predictors (110 in total) comprised: CDR3 length (length) and global biophysical properties (isoelectric point pI, log10-transformed molecular weight, hydropathicity, and average residue size); Amino-acid composition: frequencies of each of the 20 standard amino acids within the CDR3 motif region (*_CDR3mr), V-motif (*_Vmotif), and J-motif (*_Jmotif) (CDR3mr, Vmotif, and Jmotif were acquired utilizing the TiRP pipeline^14^); 5 Atchley factor scores13 in distinct positions in the CDR3mr: quantitative representation of biochemical properties (including polarity, secondary structure, molecular weight, codon diversity, and electrostatic charge) at each aligned position of the CDR3mr; V- and J-gene usage (commented out in final model): exploratory analyses encoded TRBV/TRBJ identity as five random numeric factors per gene (to avoid arbitrary ordering), but these were removed after backward stepwise selection failed to improve predictive performance.

Variable selection procedure: Full model (110 candidate predictors) was fitted with all biologically plausible features. Backward stepwise elimination based on Akaike Information Criterion (AIC) was applied to remove non-contributing variables, yielding the reduced model (88 predictors). Multicollinearity was monitored by calculating Variance Inflation Factor (VIF); no retained variable exceeded VIF = 5. Model comparison: The reduced model (88 predictors) was compared with the full model by a likelihood-ratio χ² test: Resid. Dev difference: 11301; df: 18; p < 2.2 × 10⁻¹⁶. Performance: final model achieved AUC = 0.851 (95 % CI 0.699–0.896) on the combined training + test set (internal validation). All analyses were performed in R 4.3.0 using the built-in glm() function with family = binomial(link = "logit").

### Processing of reference database immune repertoire data

The PBMC immune repertoire dataset used in the comparison between the thymus and the PBMC was acquired from the immune ACCESS database. To eliminate the error made by individual variation, all the related C57BL/6-derived datasets were downloaded and integrated into a total dataset by counting the number of all the specific clonotypes.

# Supplementary figures

## Figure S1


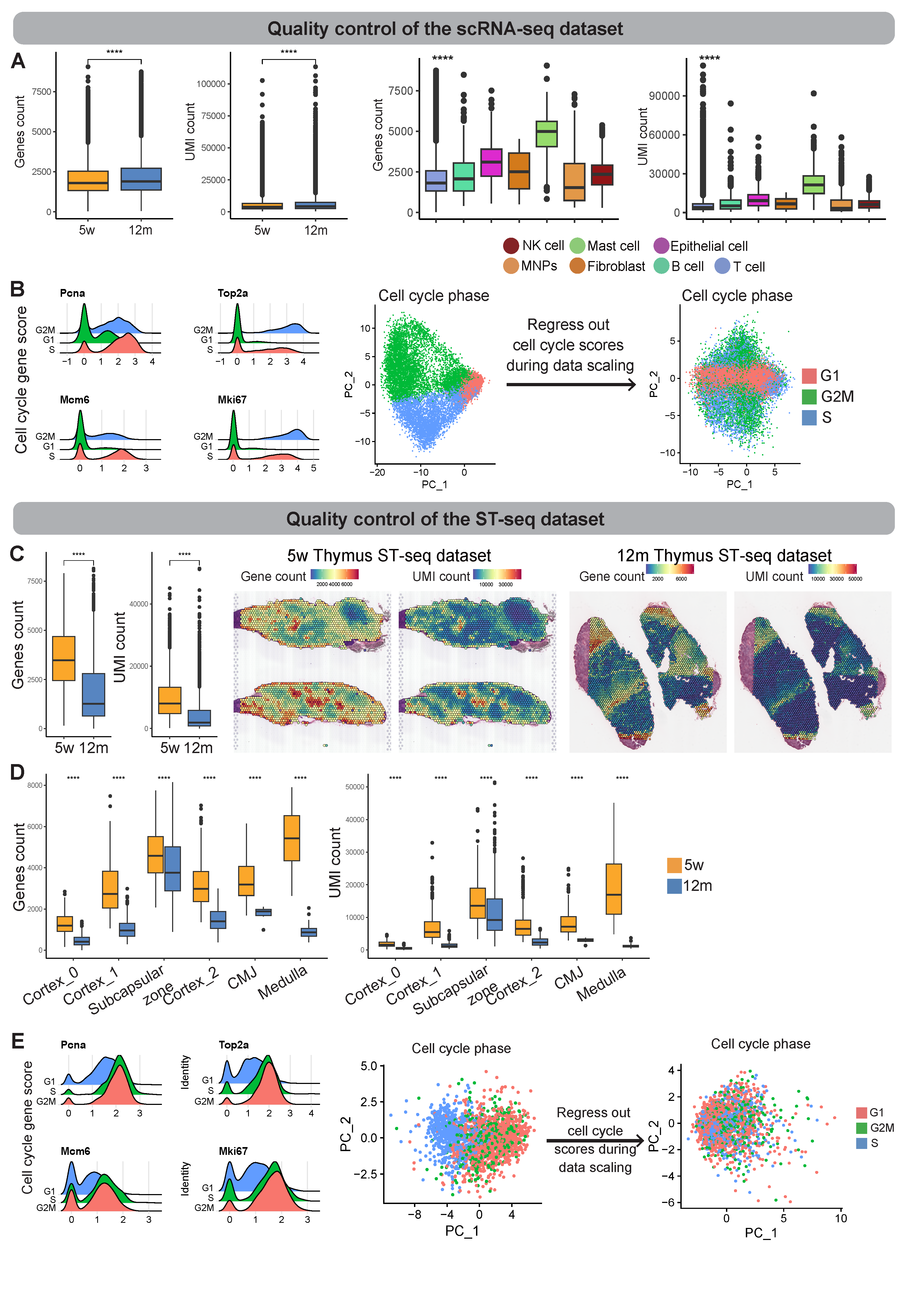


## Figure S1. Quality control of the ST-seq data and scRNA-seq data of thymus samples

1. Box plots indicating gene counts and UMI count of the thymus scRNA-seq data grouped by distinct ages (left) and cell types (right). Significances were calculated with the t-test and anova methods (ns: p>0.05; *: p<0.05; **: p<0.01; ***: p<0.001; ****: p<0.0001).
2. Regressing out cell cycle scores in the scRNA-seq data. Left, expression levels of cell cycle-related genes by states. Right, PCA of ST-seq barcodes labeled by distinct cell cycle states.
3. Left, box plots depicting genes count and UMI count of the thymus ST-seq library. Right, spatial feature plots of genes count and UMI count of the thymus ST-seq library. Significances were calculated with the t-test method (ns: p>0.05; *: p<0.05; **: p<0.01; ***: p<0.001; ****: p<0.0001).
4. Box plots and the scatter plot of genes count and UMI count of the ST-seq data by distinct sub-structures of distinct thymus samples. Significances were calculated with the t-test method (ns: p>0.05; *: p<0.05; **: p<0.01; ***: p<0.001; ****: p<0.0001).
5. Regressing out cell cycle scores in the ST-seq data. Left, expression levels of cell cycle-related genes by states. Right, PCA of ST-seq barcodes labeled by distinct cell cycle states.

## Figure S2


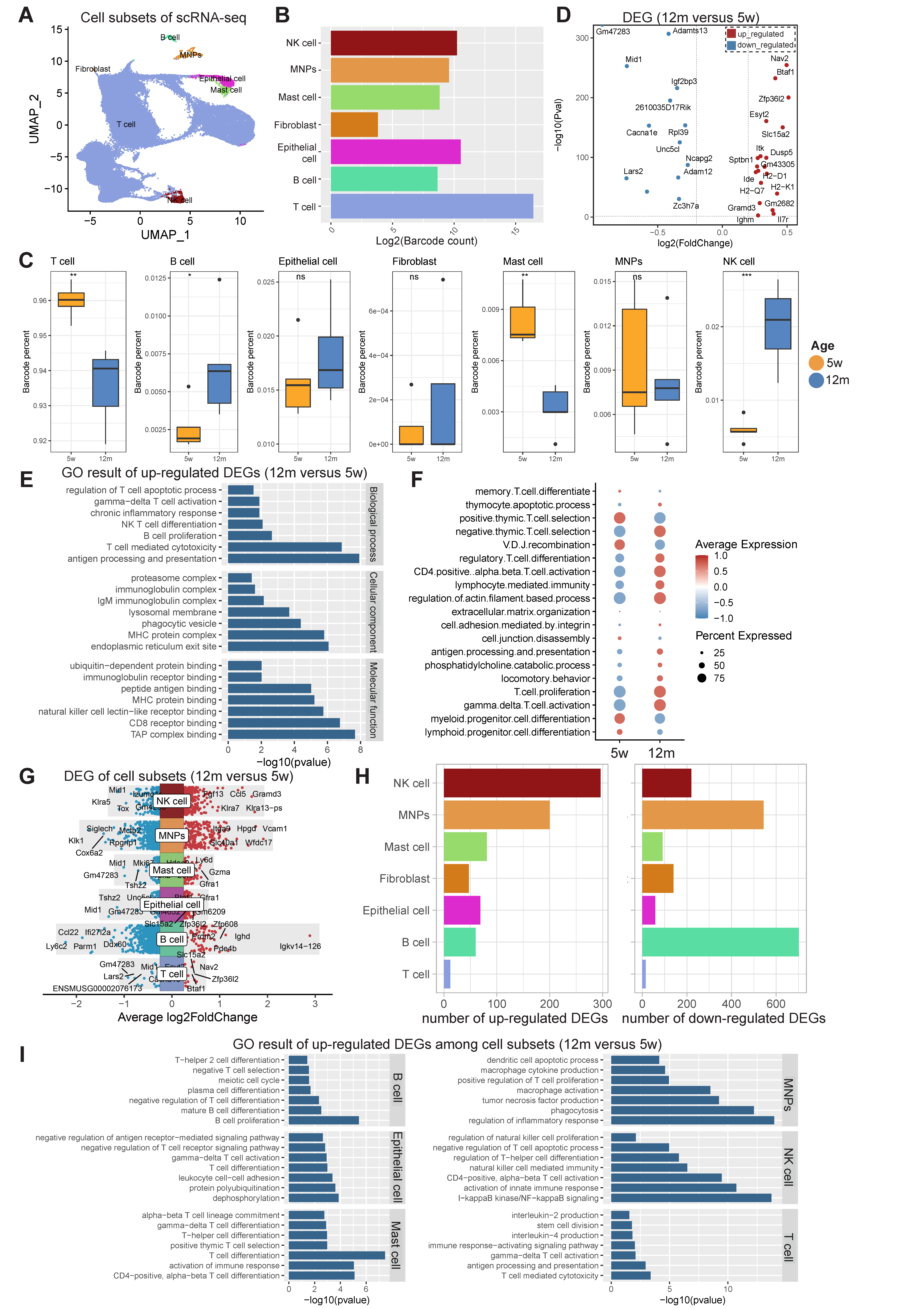


## Figure S2. Analyses of distinct cell populations of the thymus during aging of the scRNA-seq data

1. The uniform manifold approximation and projection (UMAP) of scRNA-seq barcodes recovered from thymus samples labeled by cell types.
2. Bar plots of the proportion of cell types by the total barcode number.
3. Box plots indicating the proportion of distinct cell subsets during the aging. The t-test was used to calculate the significance (ns: p>0.05; *: p<0.05; **: p<0.01; ***: p<0.001; ****: p<0.0001).
4. The scatter plot indicating differential genes of the aging thymus (12m) compared to the young thymus (5w).
5. Bar plots of enriched signature pathways of differential genes in the aging thymus (12m) using the GO analyses. Differential genes (p_val < 0.05 and log2foldchange >0) were used to perform the gene ontology analysis.
6. The dot plot indicating differences in several signatures between samples of distinct ages.
7. The scatter plot indicating differential genes of the aging thymus (12m) compared to the young thymus (5w) in distinct cell types.
8. Bar plots indicating the number of up-regulated and down-regulated DEGs of distinct cell types.
9. Bar plots of enriched signature pathways of differential genes in the aging thymus (12m) in distinct cell types using the GO analyses. Differential genes (p_val < 0.05 and log2foldchange >0) were used to perform the gene ontology analysis.

## Figure S3


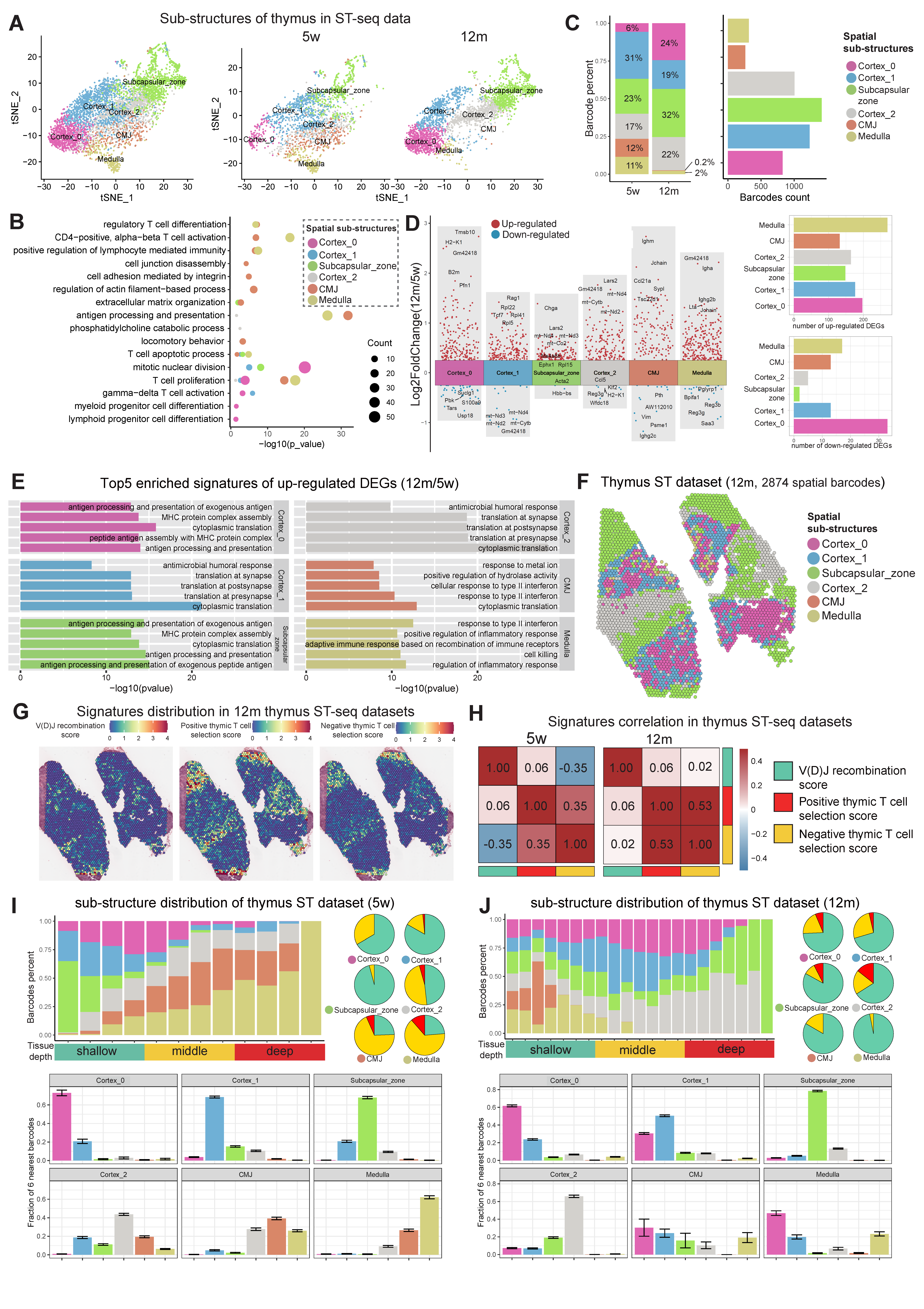


## Figure S3. Analyses of distinct sub-structures of the thymus ST-seq data

1. TSNE reduction plots of ST-seq barcodes labeled by 6 sub-structures (left) and distinct samples (right).
2. Enriched scores of specific signaling pathways of each sub-structure for the identification.
3. Bar plots of the proportion of sub-structures in distinct samples (left) and the total barcode number (right).
4. Scatter plots of differentiated genes of distinct sub-structures in the ST-seq data (12m versus 5w) (left) and bar plot indicating the number of up-regulated and down-regulated DEGs of distinct histology sub-structures (right).
5. Bar plots of enriched signature pathways of differential genes in the aging thymus (12m) in distinct histology sub-structures using the GO analyses. Differential genes (p_val < 0.05 and log2foldchange >0) were used to perform the gene ontology analysis.
6. Clustering of ST spots based on the tissue regions and annotated with distinct histological sub-structures of the 12-month thymus sample.
7. Spatial feature plots of several vital signatures including V(D)J recombination, positive thymic T cell selection, and negative thymic T cell selection of the 12-month thymus
8. Heat maps indicating the correlation of signatures in distinct ST-seq samples. The pearson was used to calculate the correlation.
9. Bar plots of the tissue depth of sub-structures in the 5-week thymus. The tissue depth was equally divided and pie charts of sub-structures in distinct tissue depth ranges. The bottom bar plot indicating the annotation of the nearest 6 barcodes of the specific barcode of sub-structures.
10. Bar plots of the tissue depth of sub-structures in the 12-month thymus. The tissue depth was equally divided and pie charts of sub-structures in distinct tissue depth ranges. The bottom bar plot indicating the annotation of the nearest 6 barcodes of the specific barcode of sub-structures.

## Figure S4





## Figure S4. Validation of transcriptomic features and spatial distribution of the Cortex_0 in the SeekSpace single-cell spatial transcriptomics dataset

1. The uniform manifold approximation and projection (UMAP) of scRNA-seq barcodes recovered from the thymus sample of distinct ages labeled by cell types of the SeekSpace platform.
2. The dot plot for expression of marker genes in distinct cell populations. Color represents the maximum-normalized mean expression of marker genes in each cell type, and size indicates the proportion of cells expressing marker genes.
3. The spatial distribution of scRNA-seq barcodes recovered from the thymus sample of distinct ages labeled by distinct histological sub-structures of the SeekSpace platform.
4. The dot plot for expression of sub-structures specific score (composed of up-regulated DEGs of distinct sub-structures in the 10X Visium platform, p value < 0.05 and log2Foldchange > 0) in distinct histological sub-structures. Color represents the maximum-normalized mean expression of marker genes in each cell type, and size indicates the proportion of cells expressing marker genes.
5. The dot plot for expression of selected genes (left) and specific signatures (right) in distinct histological sub-structures. Color represents the maximum-normalized mean expression of marker genes in each cell type, and size indicates the proportion of cells expressing marker genes.
6. The scatter plot of differential expressed genes (left) and enriched signature (right) of the Cortex_0 in the SeekSpace platform.
7. Box plots indicating the minimal inter-barcode distance among spatially distinct histological sub-structures barcodes during aging; this metric serves as an inverse proxy for structural cohesion. Significances were calculated with the t-test method (ns: p>0.05; *: p<0.05; **: p<0.01; ***: p<0.001; ****: p<0.0001).
8. The spatial distribution relationship between the Cortex_0 and CMJ & Medulla in the SeekSpace platform.

## Figure S5

## Figure S5. Validation of transcriptomic features and spatial distribution of the Cortex_0 in the 10X Visium human spatial transcriptomics dataset

1. The uniform manifold approximation and projection (UMAP) of scRNA-seq barcodes recovered from the thymus sample of distinct ages labeled by histological sub-structures in the 10X Visium human spatial transcriptomics dataset.
2. The dot plot for expression of sub-structures specific score (composed of up-regulated DEGs of distinct sub-structures in the 10X Visium platform, p value < 0.05 and log2Foldchange > 0) in distinct histological sub-structures. Color represents the maximum-normalized mean expression of marker genes in each cell type, and size indicates the proportion of cells expressing marker genes.
3. The dot plot for expression of selected genes in distinct histological sub-structures. Color represents the maximum-normalized mean expression of marker genes in each cell type, and size indicates the proportion of cells expressing marker genes.
4. The spatial distribution of scRNA-seq barcodes recovered from the thymus sample of distinct ages labeled by distinct histological sub-structures of the 10X Visium human spatial transcriptomics dataset.

## Figure S6


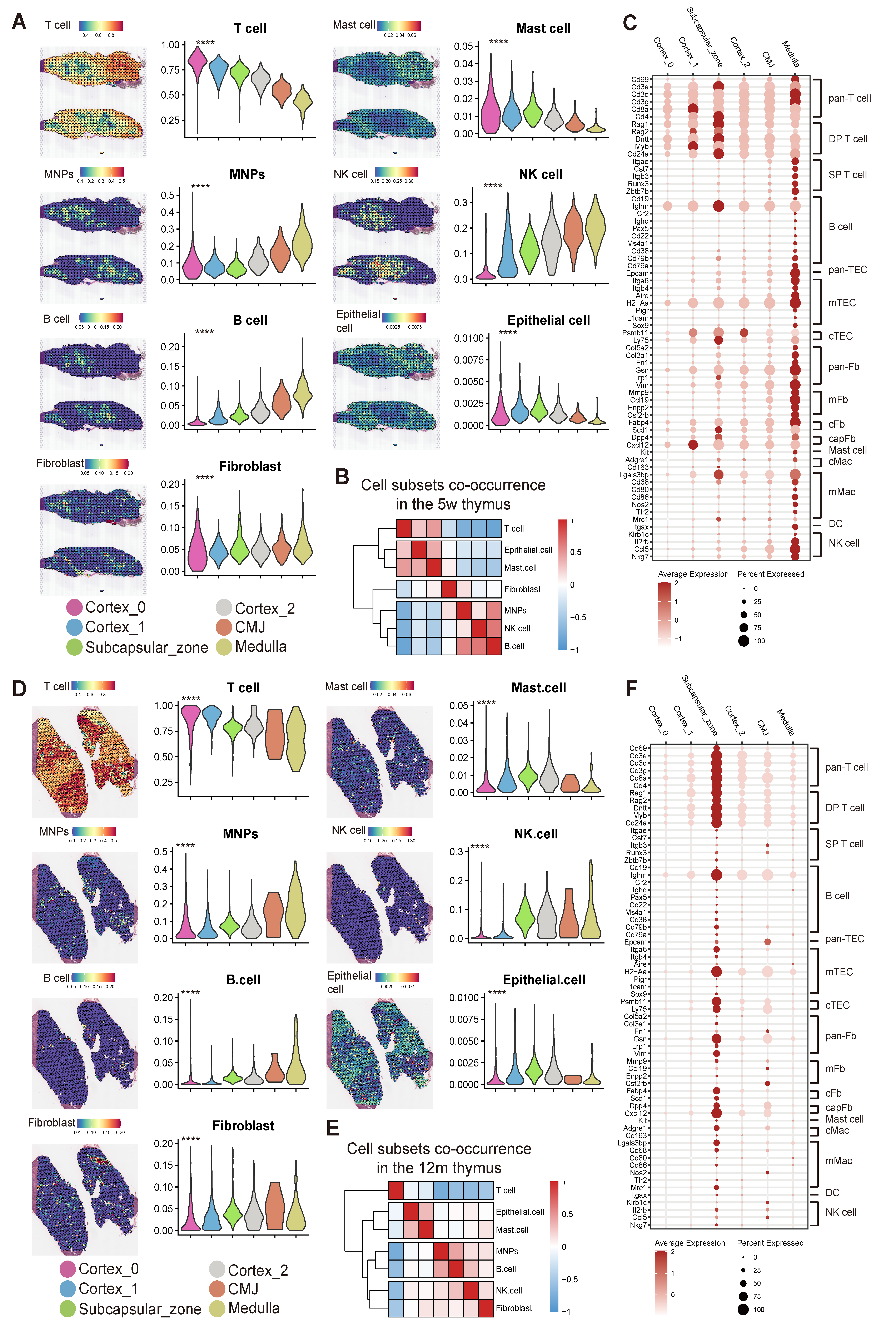


## Figure S6. The predicted distribution of cell subsets in ST-seq datasets using the CARD deconvolution pipeline

1. Spatial distribution of the predicted percent of distinct cell types in the 5w ST-seq dataset. Significances were calculated with the anova method (ns: p>0.05; *: p<0.05; **: p<0.01; ***: p<0.001; ****: p<0.0001).
2. The heatmap indicating the same-spot co-occurrence of distinct cell types in the 5w ST-seq dataset. The pearson was used to calculate the correlation.
3. The dot plot indicating marker genes of distinct cell types in distinct regions of the 5w ST-seq dataset. Color represents the maximum-normalized mean expression of marker genes in each cell type, and size indicates the proportion of cells expressing marker genes.
4. Spatial distribution of the predicted percent of distinct cell types in the 12m ST-seq dataset. Significances were calculated with the anova method (ns: p>0.05; *: p<0.05; **: p<0.01; ***: p<0.001; ****: p<0.0001).
5. The heatmap indicating the same-spot co-occurrence of distinct cell types in the 12m ST-seq dataset. The pearson was used to calculate the correlation.
6. The dot plot indicating marker genes of distinct cell types in distinct regions of the 12m ST-seq dataset. Color represents the maximum-normalized mean expression of marker genes in each cell type, and size indicates the proportion of cells expressing marker genes.

## Figure S7


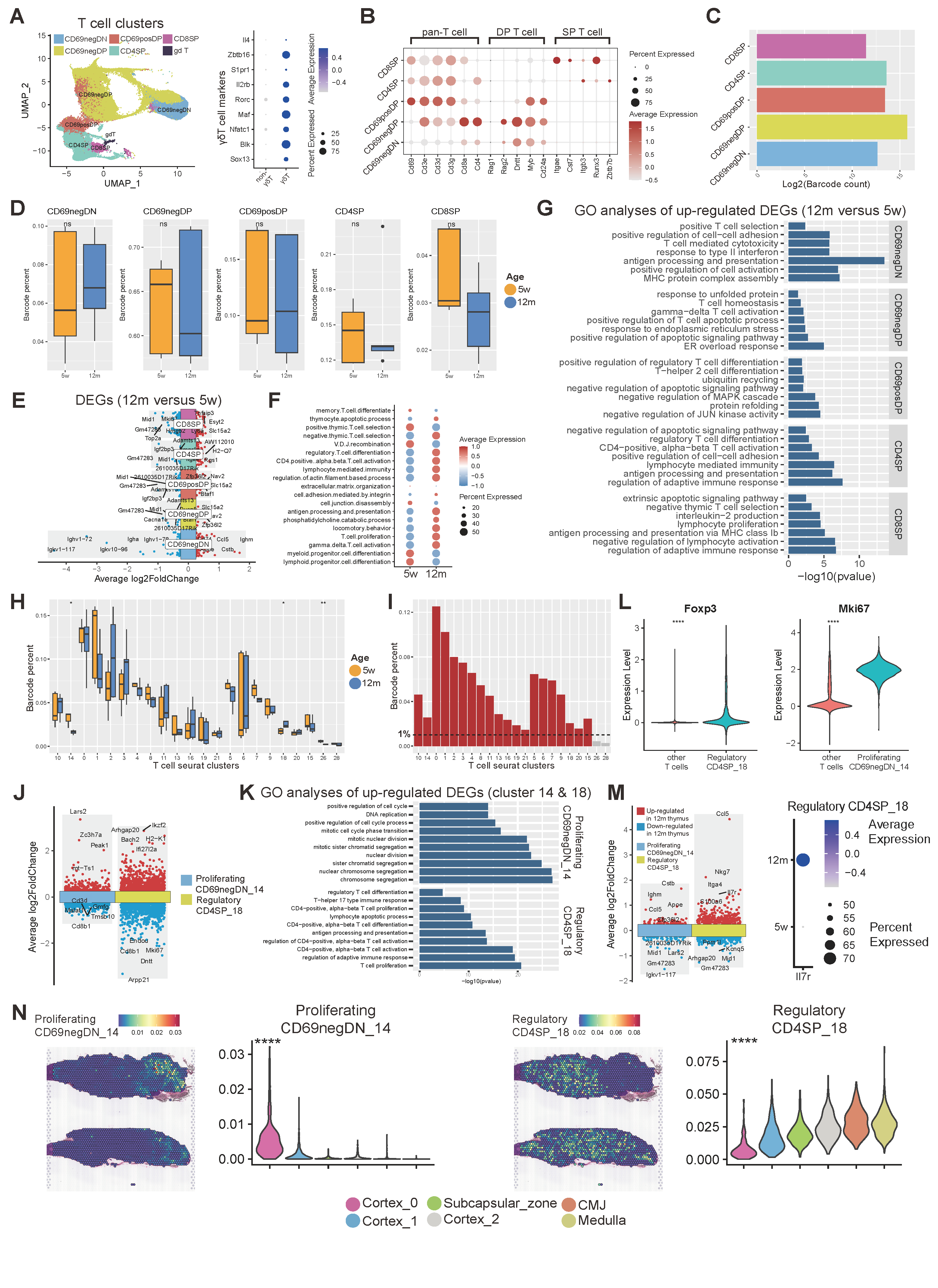


## Figure S7. Analyses of T cell lineage of the thymus during aging in scRNA-seq libraries

1. Left, the UMAP of scRNA-seq barcodes grouped by distinct Seurat clusters and labeled by T cell subsets. Right, the dot plot indicating the expression of γδT cell marker genes in T cell subsets. Right, the bar plot indicating the total cell number of T cell subsets.
2. The dot plot for expression of marker genes in distinct T cell subsets for identification. Color represents the maximum-normalized mean expression of marker genes in each cell type, and size indicates the proportion of cells expressing marker genes.
3. The bar plot indicating the total cell number of T cell subsets.
4. Box plots indicating the proportion of distinct T cell subsets during the aging. The t-test was used to calculate the significance (ns: p>0.05; *: p<0.05; **: p<0.01; ***: p<0.001; ****: p<0.0001).
5. The dot plot indicating differences in several signatures between T cells of distinct ages.
6. The scatter plot indicating differential genes of the aging thymus (12m) compared to the young thymus (5w) in distinct T cell subsets.
7. Bar plots indicating enriched signatures of each T cell subset in aging samples compared to young samples. Differential genes (p_val < 0.05 and log2foldchange >0) were used to perform the gene ontology analysis.
8. Box plots indicating the proportion of distinct T cell Seurat subsets during the aging. The t-test was used to calculate the significance (ns: p>0.05; *: p<0.05; **: p<0.01; ***: p<0.001; ****: p<0.0001).
9. The bar plot indicating the number of distinct T cell Seurat subsets.
10. The scatter plot of differential genes of CD69negDN_14 and CD4SP_18 in young samples compared to other T cell subsets.
11. The bar plot of enriched signatures of genes in the Fig S5J. Differential genes (p_val < 0.05 and log2foldchange >0) were used to perform the gene ontology analysis.
12. Violin plots indicating the expression of Mki67 and Foxp3 in CD69negDN_14, CD4SP_18, and other cells. Significances were calculated with the t-test method (ns: p>0.05; *: p<0.05; **: p<0.01; ***: p<0.001; ****: p<0.0001).
13. Left, the scatter plot of differential genes of CD69negDN_14 and CD4SP_18 in aging samples compared to young samples. Right, the dot plot indicating the expression of Il7r in CD4SP_16 during aging.
14. The predicted spatial distribution of CD69negDN_14 and CD4SP_18 in the 5w thymus ST-seq data. Significances were calculated with the anova method (ns: p>0.05; *: p<0.05; **: p<0.01; ***: p<0.001; ****: p<0.0001).

## Figure S8


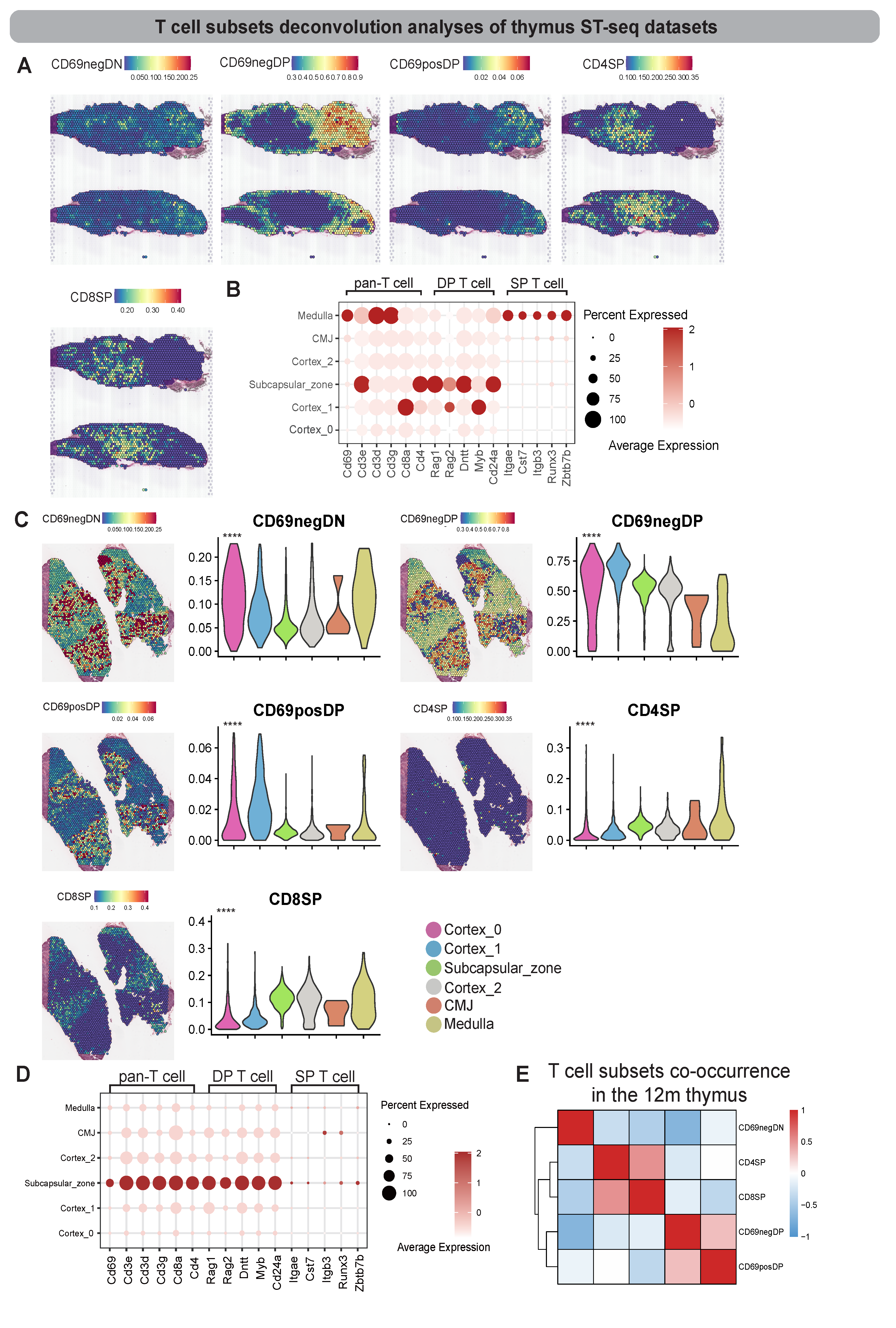


## Figure S8. The predicted distribution of T cell subsets in ST-seq datasets using the CARD deconvolution pipeline

1. Spatial distribution of the predicted percent of distinct T cell subsets in the 5w ST-seq data.
2. The dot plot indicating marker genes of T cell subsets in distinct regions of the 5w thymus ST-seq data. Color represents the maximum-normalized mean expression of marker genes in each cell type, and size indicates the proportion of cells expressing marker genes.
3. Spatial distribution of the predicted percent of distinct T cell subsets in the 12m ST-seq data. Significances were calculated with the anova method (ns: p>0.05; *: p<0.05; **: p<0.01; ***: p<0.001; ****: p<0.0001).
4. The dot plot indicating marker genes of T cell subsets in distinct regions of the 12m thymus ST-seq data. Color represents the maximum-normalized mean expression of marker genes in each cell type, and size indicates the proportion of cells expressing marker genes.
5. The heatmap indicating the same-spot co-occurrence of distinct T cell subsets in the 12-month ST-seq data. The pearson was used to calculate the correlation.

## Figure S9


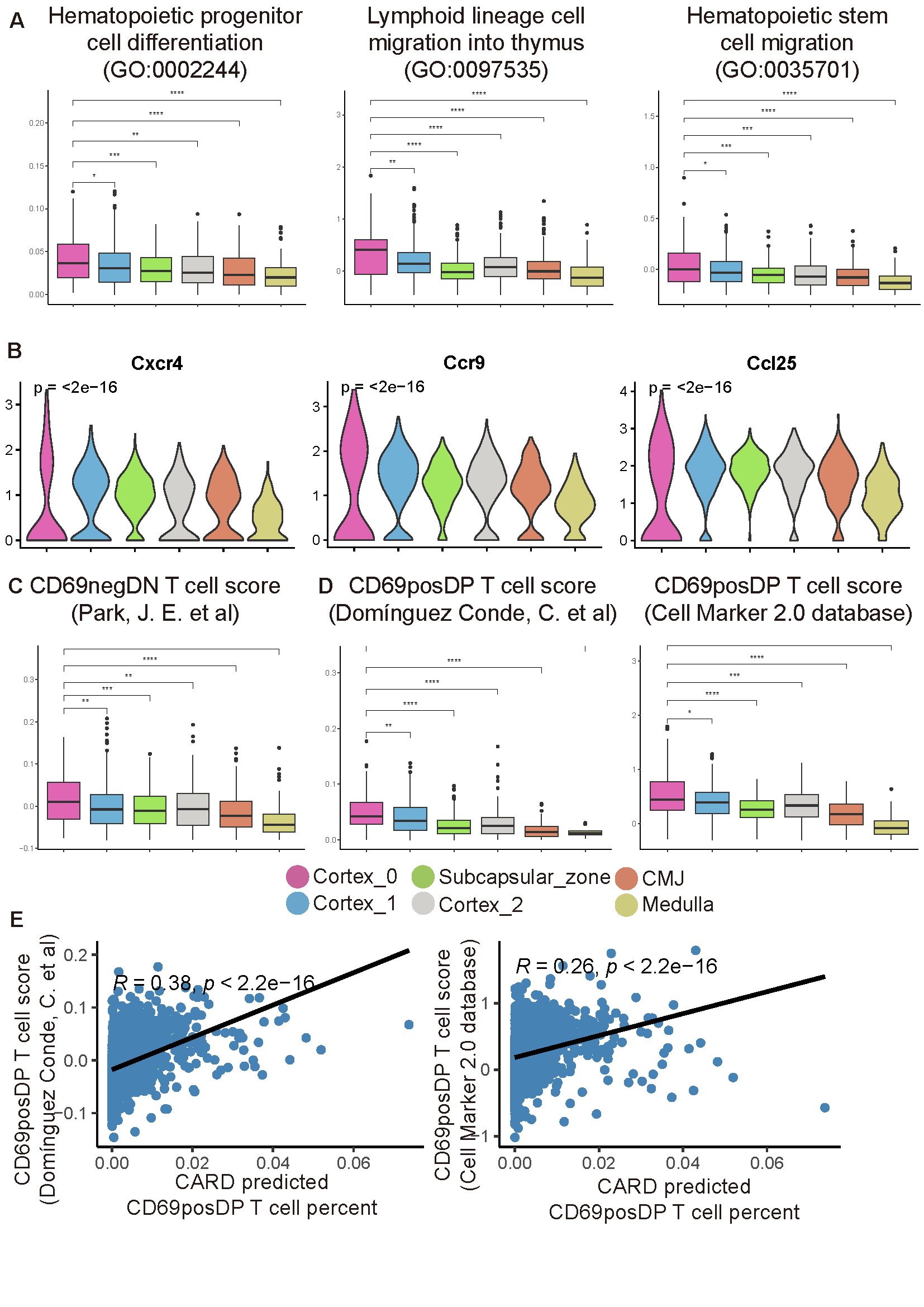


## Figure S9. The validation of the spatial distribution prediction of CD69negDN T cells and CD69posDP T cells utilizing published datasets and databases

1. Box plots indicating several signatures in distinct sub-structures of thymus from the ST-seq dataset. Signatures include genes acquired from the gene ontology database and calculated with the AddModule function in the Seurat package. Significances were calculated with the t-test method (ns: p>0.05; *: p<0.05; **: p<0.01; ***: p<0.001; ****: p<0.0001).
2. Violin plots indicating several genes related with the migration of progenitor T cells into the thymus from the ST-seq dataset. Significances were calculated with the anova method.
3. The box plot indicating the enrichment of CD69negDN T cells in the ST-seq thymus dataset. Significances were calculated with the t-test method (ns: p>0.05; *: p<0.05; **: p<0.01; ***: p<0.001; ****: p<0.0001).
4. Box plots indicating the predicted percent of CD69posDP T cells (left), the expression of CD69posDP T cells scores consisting of marker genes acquired from Dominguez Conde, C et al research (middle) and Cell Marker 2.0 database (right) in the thymus ST-seq dataset. Significances were calculated with the t-test method (ns: p>0.05; *: p<0.05; **: p<0.01; ***: p<0.001; ****: p<0.0001).
5. Correlation of the predicted percent of CD69posDP T cells and CD69posDP T cells scores. Significances were calculated with the t-test method and the correlation was calculated with the spearman method.

## Figure S10


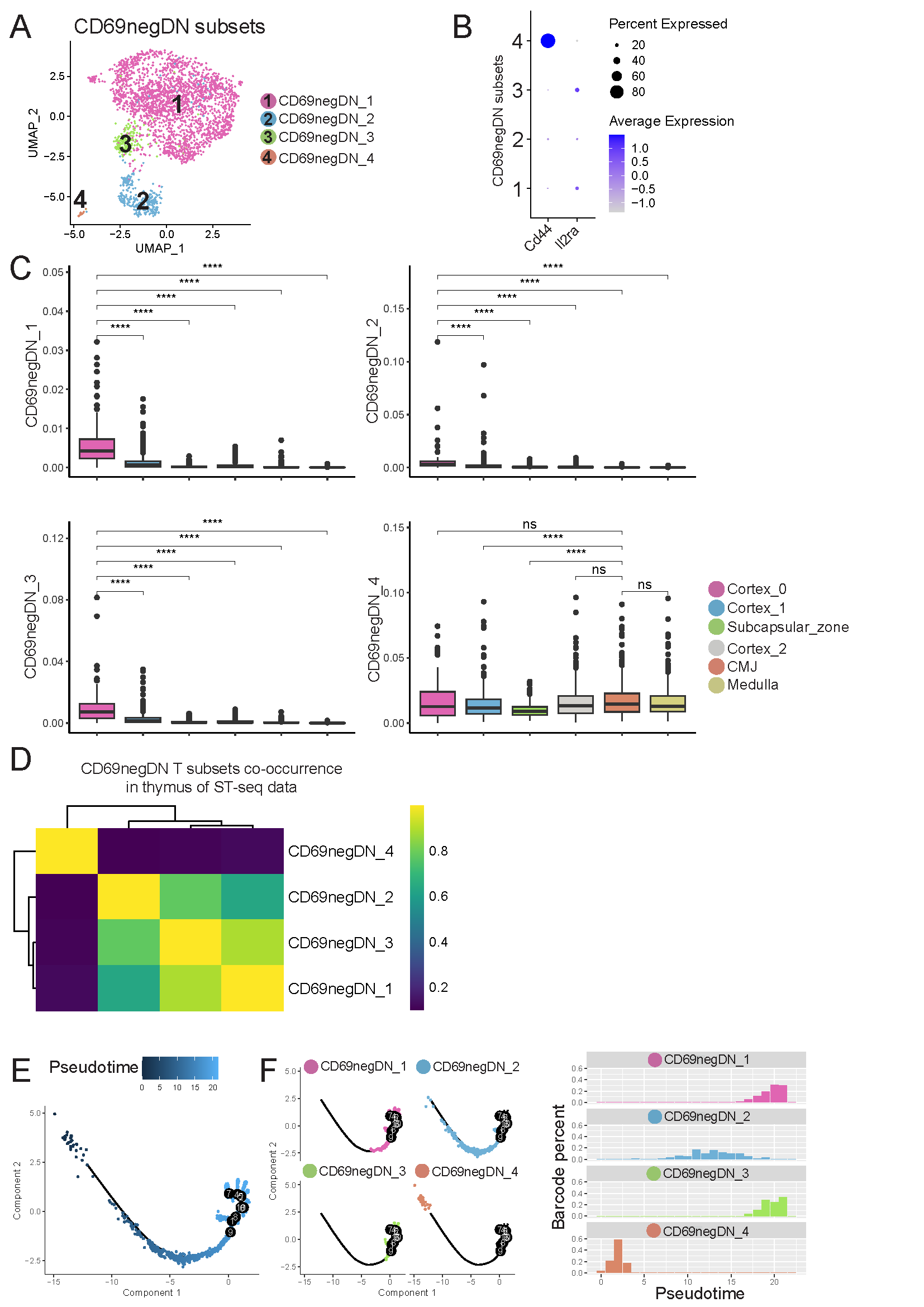


## Figure S10. The heterogeneity of CD69negDN T cells demonstrated the differentiation and migration in the thymus

1. The uniform manifold approximation and projection (UMAP) of scRNA-seq barcodes recovered of CD69negDN T cells labeled by minor Seurat clusters.
2. The dot plot indicating several marker genes between distinct CD69negDN T subsets.
3. The box plot indicating the distribution of CD69negDN T cells in the ST-seq thymus dataset using CARD pipeline. Significances were calculated with the t-test method (ns: p>0.05; *: p<0.05; **: p<0.01; ***: p<0.001; ****: p<0.0001).
4. Correlation of the predicted percent of CD69posDP T cells and CD69posDP T cells scores. The pearson was used to calculate the correlation.
5. The pseudo-time trajectory order of CD69negDN T cells in the scRNA-seq dataset.
6. Left, the pseudo-time trajectory order of CD69negDN T cells labeled by minor Seurat clusters in the scRNA-seq dataset. Right, the bar plot indicating the distribution of the pseudo-time trajectory order of CD69negDN T cells labeled by minor Seurat clusters in the scRNA-seq dataset.

## Figure S11


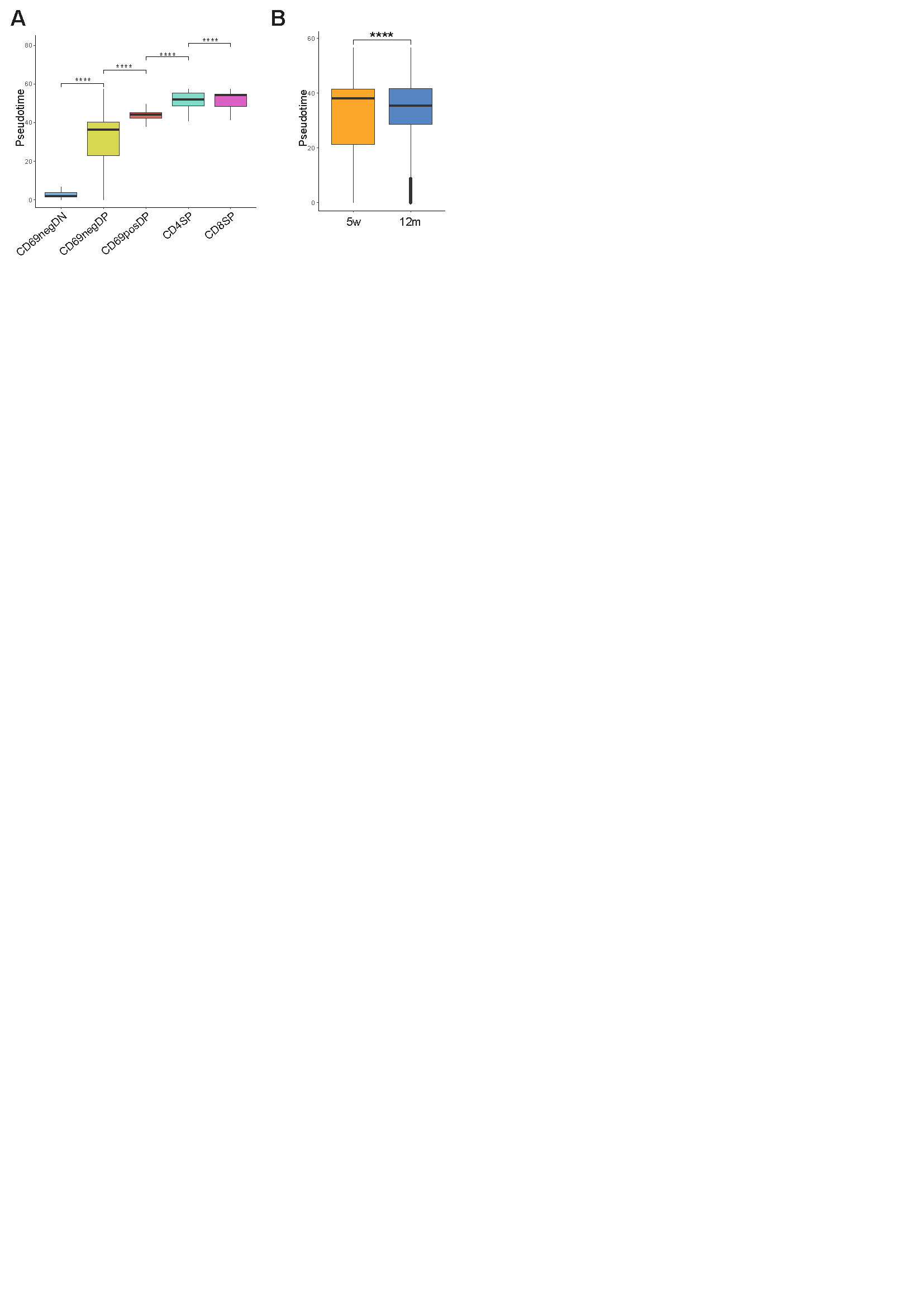


## Figure S11. The single-cell pseudo-time trajectory of T cells during aging in the scRNA-seq data

1. The box plot of the trajectory of distinct T cell subsets. Significances were calculated with the t-test test method (ns: p>0.05; *: p<0.05; **: p<0.01; ***: p<0.001; ****: p<0.0001).
2. The box plot of the trajectory of distinct ages. Significances were calculated with the t-test test method (ns: p>0.05; *: p<0.05; **: p<0.01; ***: p<0.001; ****: p<0.0001)

## Figure S12


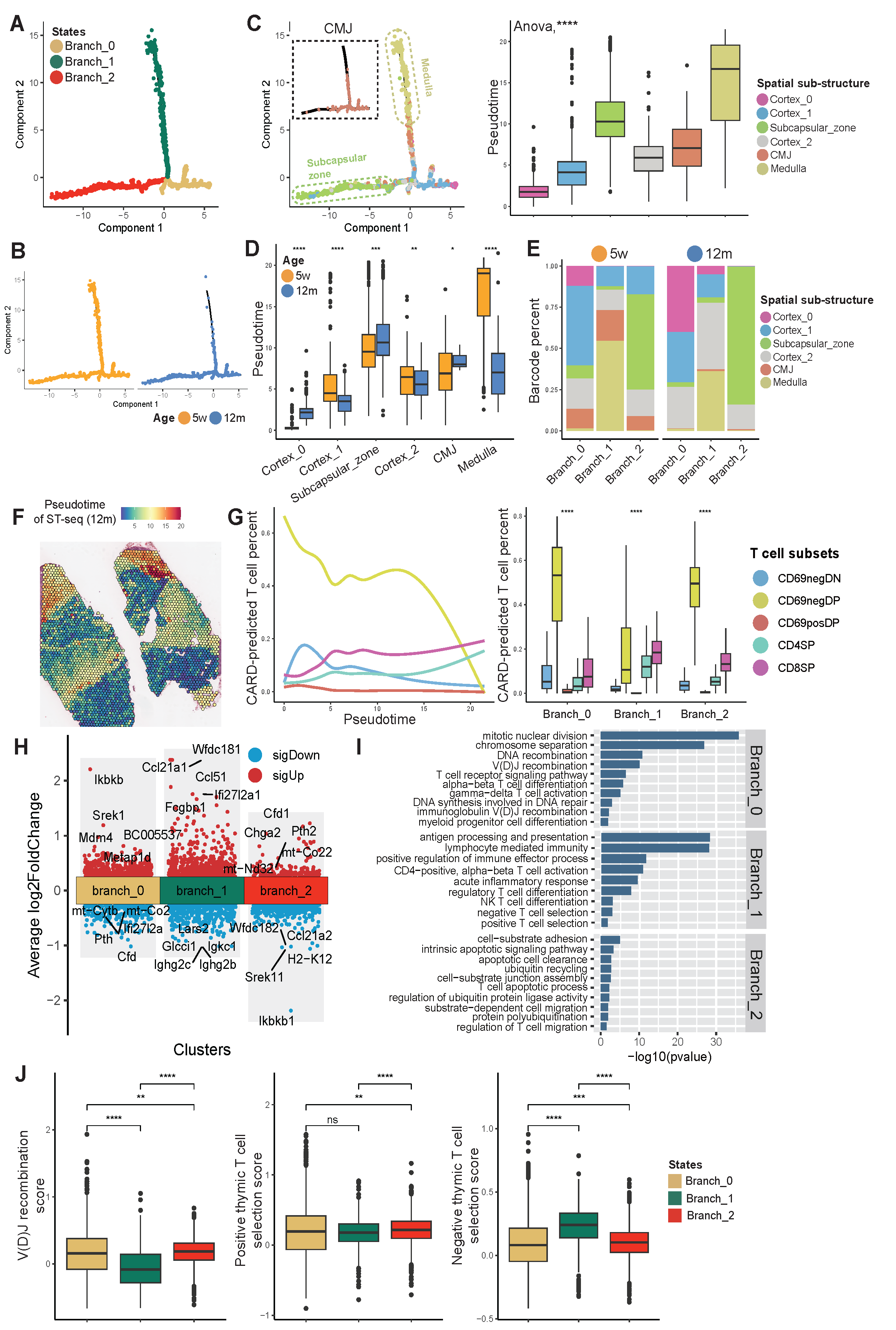


## Figure S12. The spatial pseudo-time trajectory of the thymus in the ST-seq data

1. The trajectory order of distinct states decided by the structure of the trajectory.
2. The trajectory order of distinct states decided by distinct ages.
3. The trajectory order (left) and the box plot (right) of the correlation between pseudo-time trajectory and sub-structures. Significances were calculated with the anova method (ns: p>0.05; *: p<0.05; **: p<0.01; ***: p<0.001; ****: p<0.0001). The trajectory of the CMJ was separately posited in the top left.
4. The box plot of the correlation between pseudo-time trajectory and sub-structures in distinct samples. Significances were calculated with the t-test method (ns: p>0.05; *: p<0.05; **: p<0.01; ***: p<0.001; ****: p<0.0001).
5. Stacked bar plot indicating proportions of sub-structures in pseudo-time trajectory states.
6. The spatial distribution of the pseudo-time trajectory in the 12-month sample.
7. The line plot (left) indicating the trajectory order of distinct T cell subpopulations predicted using the corresponding scRNA-seq dataset and the box plot (right) indicating the predicted percent of T cell subsets in each state. Significances were calculated with the anova method (ns: p>0.05; *: p<0.05; **: p<0.01; ***: p<0.001; ****: p<0.0001).
8. The scatter plot of differentiated genes of distinct states in the trajectory order.
9. Bar plots of enriched signaling pathways of each state in the trajectory order. Differential genes (p_val < 0.05 and log2foldchange >0) were used to perform the gene ontology analysis.
10. Box plots indicating the expression of several signatures in each state of the trajectory. Significances were calculated with the t-test method (ns: p>0.05; *: p<0.05; **: p<0.01; ***: p<0.001; ****: p<0.0001).

## Figure S13


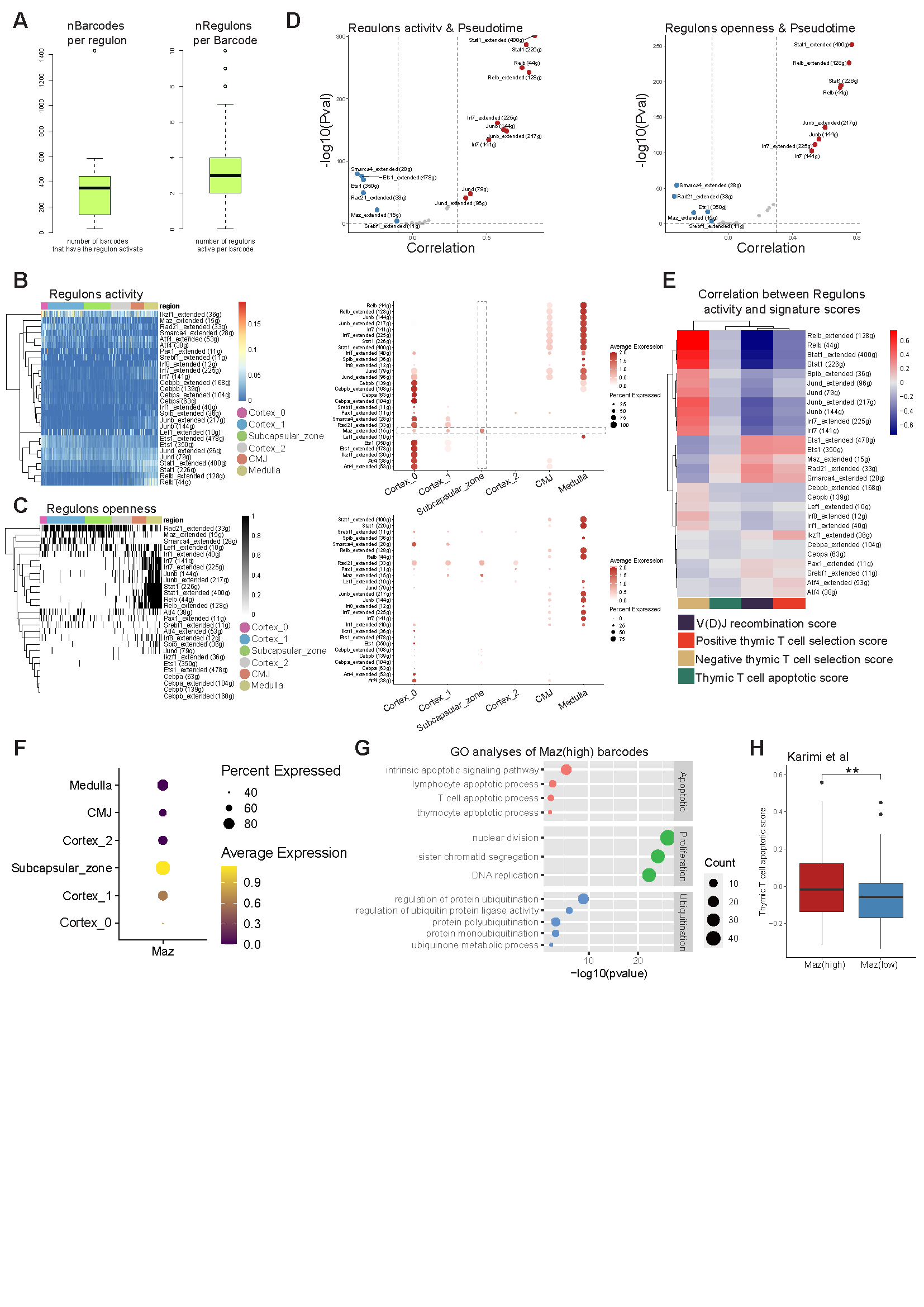


## Figure S13. Transcription factor Maz mediated thymocyte apoptotic in subcapsular zone

1. Box plots indicating the number of regulons per barcode and number of barcodes per regulon.
2. The heatmap (left) and the dot plot (right) of the activity of regulons in sub-structures.
3. The heatmap (left) and the dot plot (right) of the openness of regulons in sub-structures.
4. Scatter plots of the correlation between regulons and pseudo-time trajectory.
5. The heatmap indicating the correlation between regulons and signaling pathways.
6. The dot plot indicating the expression of TF Maz in sub-structures.
7. Scatter plots indicating enriched pathways in Maz high regions.
8. The boxplot indicating the difference of T cell apoptotic in Maz-high and Maz-low barcodes in the published scRNA-seq dataset. Significances were calculated with the t-test method (ns: p>0.05; *: p<0.05; **: p<0.01; ***: p<0.001; ****: p<0.0001).

## Figure S14


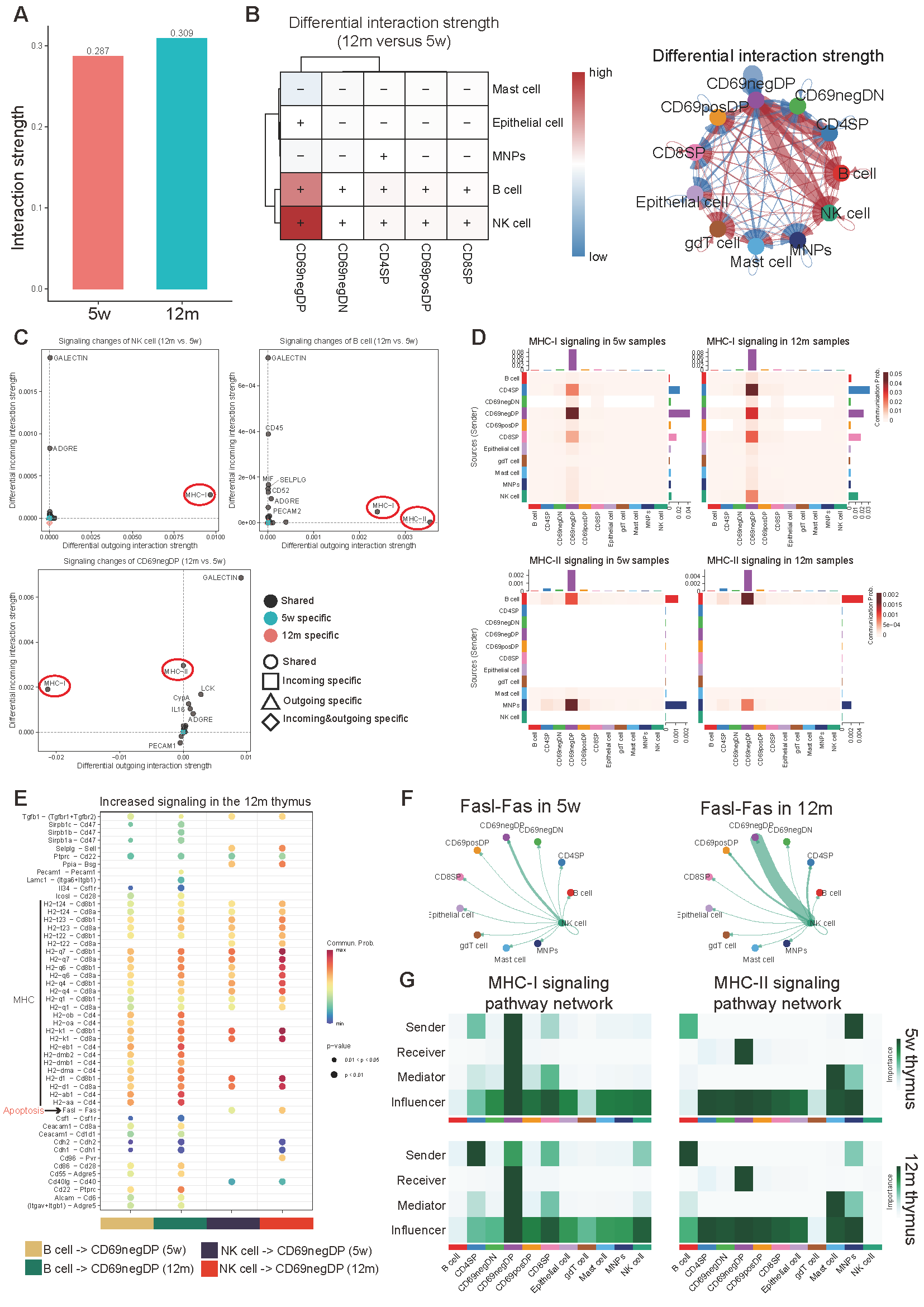


## Figure S14. Increased interactions among B cells, NK cells, and CD69negDP T cells mediated by MHC signature during aging in the scRNA-seq dataset

1. The bar plot indicating the difference in interaction strength among samples of distinct ages in the scRNA-seq dataset.
2. The heatmap (left) and the circle plot (right) indicating the interaction strength dynamic between stromal cells and T cells during aging in the scRNA-seq dataset. Red means the strength increased while blue means the strength decreased in the aging sample.
3. Scatter plots indicating the dynamic in interaction strength in several pathways in NK cells, B cells, and CD69negDP T cells in the scRNA-seq dataset.
4. Heatmaps indicating the dynamic of MHC-I and MHC-II pathways among cell types during aging in the scRNA-seq dataset.
5. The dot plot indicating increased ligand & receptor interaction among NK cells, B cells, and CD69negDP T cells during aging in the scRNA-seq dataset.
6. Circle plots indicating the dynamic of Fas-Fasl interaction among cell types during aging in the scRNA-seq dataset.
7. Heatmaps indicating roles of each cell type in MHC-I and MHC-II pathways in the scRNA-seq dataset.

## Figure S15


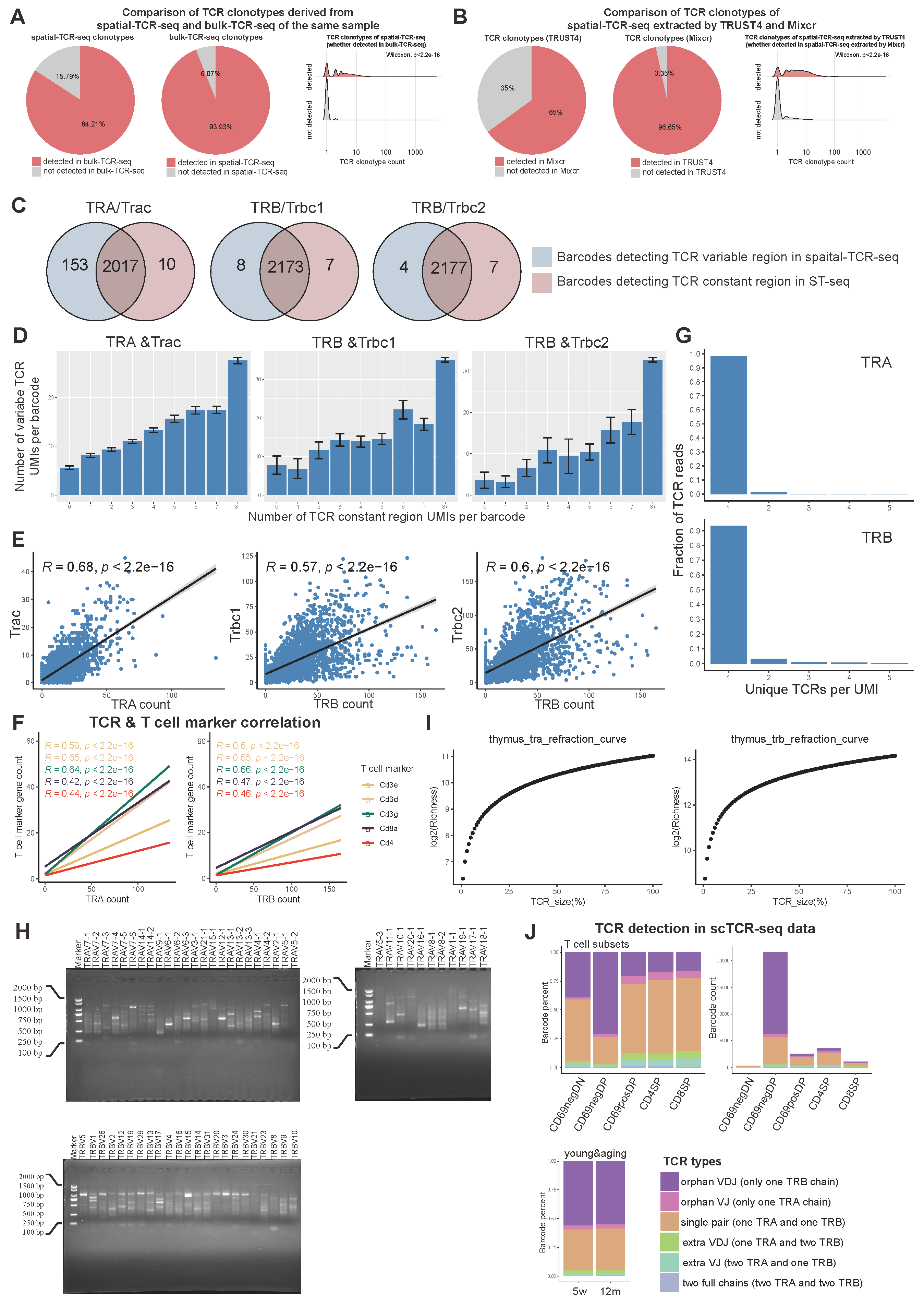


## Figure S15. Quality control of the spatial-TCR-seq data and the scTCR-seq

1. Left, the pie plot indicating the percent of TCR clonotypes in the spatial-TCR-seq that can be detected in the bulk-TCR-seq. Middle, the pie plot indicating the percent of TCR clonotypes in the bulk-TCR-seq that can be detected in the spatial-TCR-seq. Right, the ridge plot indicating the TCR clonotype count distribution between TCR clonotype of spatial-TCR-seq that can be detected or not detected in the bulk-TCR-seq.
2. Left, the pie plot indicating the percent of TCR clonotypes extracted by TRUST4 that can be extracted by MiXCR. Middle, the pie plot indicating the percent of TCR clonotypes extracted by MiXCR that can be extracted byTRUST4. Right, theridge plot indicating the TCR clonotype count distribution between TCR clonotype extracted by TRUST4 that can be detected or not detected by the MiXCR.
3. Venn plots indicating barcodes detecting the variable region and constant region of TCRα and TRBβ locus.
4. Bar plots indicating the correlation between the count of the variable region and the constant region of TCRs per barcode.
5. Scatter plots indicating the correlation between the variable region and constant regions of TCRs. Significances were calculated with the t-test method and the correlation was calculated with the spearman method.
6. Bar plots indicating the count of TCR reads per UMI detected in spatial-TCR-seq data. Significances were calculated with the t-test method and the correlation was calculated with the spearman method.
7. Line plots indicating the correlation between TCR counts and T cell marker genes. Significances were calculated with the t-test method and the correlation was calculated with the spearman method.
8. The TCR-specific amplification nucleic acid gel diagram of distinct TRAV and TRBV genes.
9. The refraction curve of the TCR clonotypes indicating the sequencing saturation of the spatial-TCR-seq. The X lab indicated the percent of the selected TCR clonotypes in the whole dataset. The Y lab indicated the number of unique TCR clonotypes types (the Richness index, which was calculated by the VDJtools pipeline^12^).
10. Bar plots indicating the proportion of barcodes detecting TRB chains and TRA chains in distinct ages and T cell subsets in the scTCR-seq data. Orphan VDJ means that only one TRB chain was detected. Orphan VJ means that only one TRA chain was detected. Single pair means that one TRB chain and one TRA chain were detected. Extra VDJ means that two TRB chains and one TRA chain were detected. Extra VJ means that two TRA chains and one TRB chain were detected. Two full chains means that two TRB chains and two TRA chains were detected.

## Figure S16


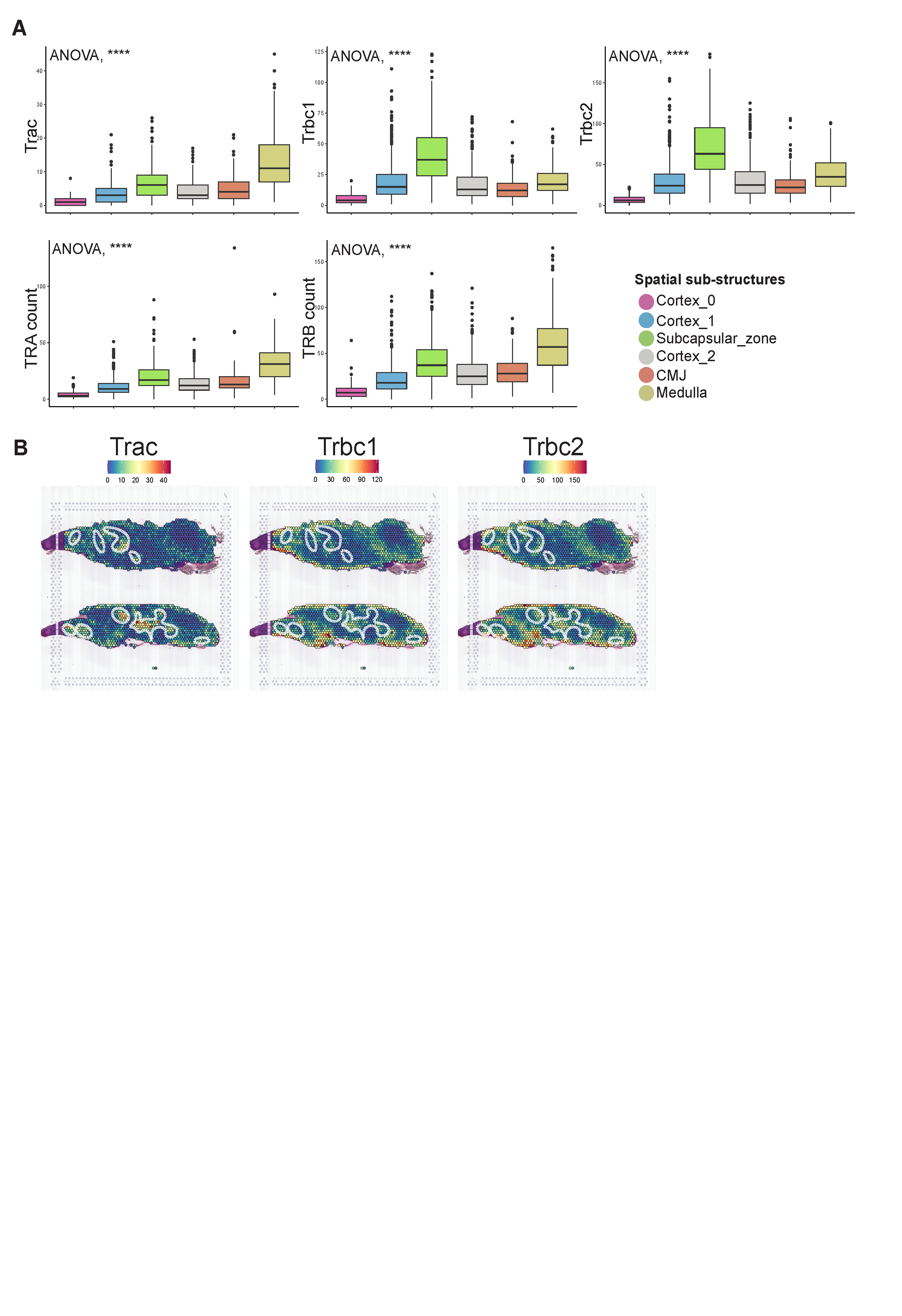


## Figure S16. The PCA reduction of the proportion of distinct TCR VJ genes, CDR3, and lengths

1. Spatial feature plots of TCRα locus constant region, and TCRβ locus constant region.
2. Box plots of pre-TCRα locus, TCRα locus, and TCRβ locus as well as their constant regions count in sub-structures. Significances were calculated with the anova method.

## Figure S17


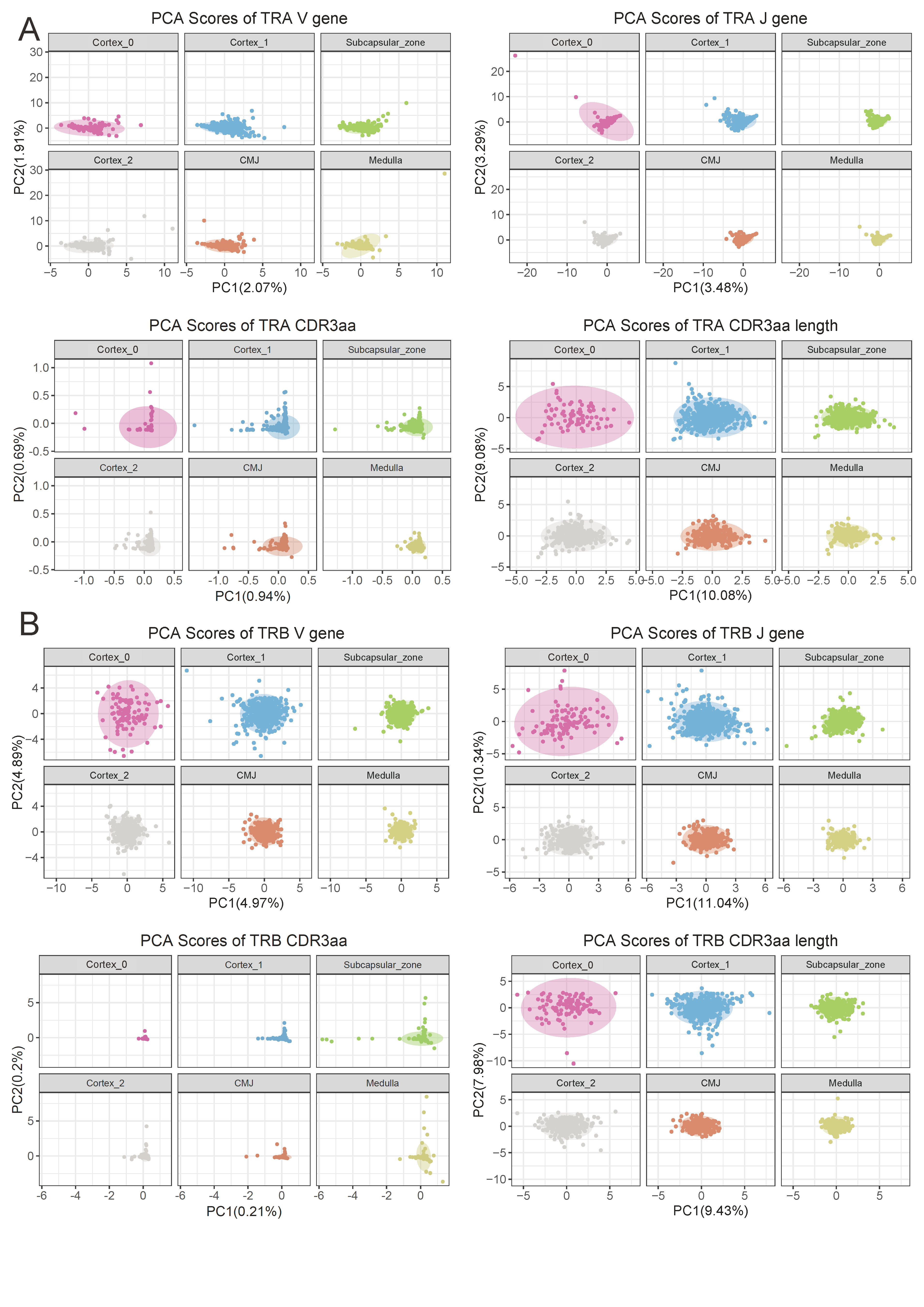


## Figure S17. The PCA reduction of the proportion of distinct TCR VJ genes, CDR3, and lengths

1. The PCA reduction of spatial barcodes based on the distribution of TRA VJ gene usage, CDR3aa distribution, and CDR3aa length.
2. The PCA reduction of spatial barcodes based on the distribution of TRB VJ gene usage, CDR3aa distribution, and CDR3aa length.

## Figure S18


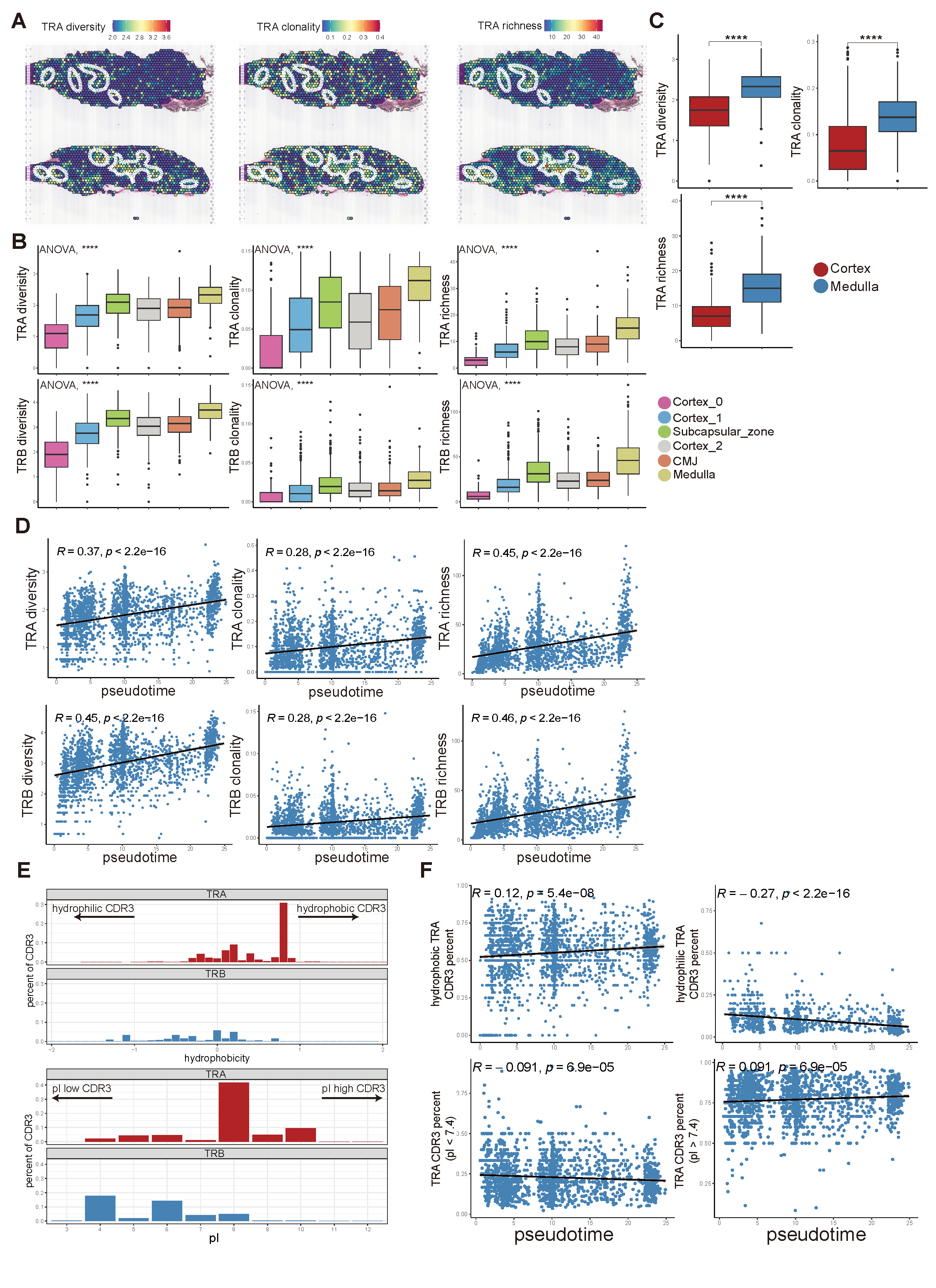


## Figure S18. Analyses of the overall spatial distribution of clonotypes

1. Spatial feature plots of diversity, clonality, and richness of TCRα locus calculated by the Vdjtools software.
2. Box plots of diversity, clonality, and richness of TCRα locus in sub-structures. Significances were calculated with the anova method (ns: p>0.05; *: p<0.05; **: p<0.01; ***: p<0.001; ****: p<0.0001).
3. Box plots of diversity, clonality, and richness of TCRα locus in the medulla and the cortex. Significances were calculated with the t-test method (ns: p>0.05; *: p<0.05; **: p<0.01; ***: p<0.001; ****: p<0.0001).
4. Scatter plots and correlation between pseudo time trajectory and diversity, clonality, and richness of TCRα and TRBβ locus. Significances were calculated with the t-test method and the correlation was calculated with the spearman method.
5. Bar plots of the distribution of the hydrophobicity and pI of different chains of CDR3s.
6. The scatterplot and correlation of the TRA CDR3aa frequency of distinct physicochemical properties including hydropathicity and electrostatic charge derived from both spatial-TCR-seq and ST-seq data with the pseudo-time trajectory. Hydrophobicity index < -0.25 means hydrophilic CDR3s. A hydrophobicity index > 0.25 means hydrophobic CDR3s. pI index > 7.4 means alkaline CDR3s which were positively charged. pI index < 7.4 means acidic CDR3s which were negatively charged. Significances were calculated with the t-test method and the correlation was calculated with the spearman method.

## Figure S19


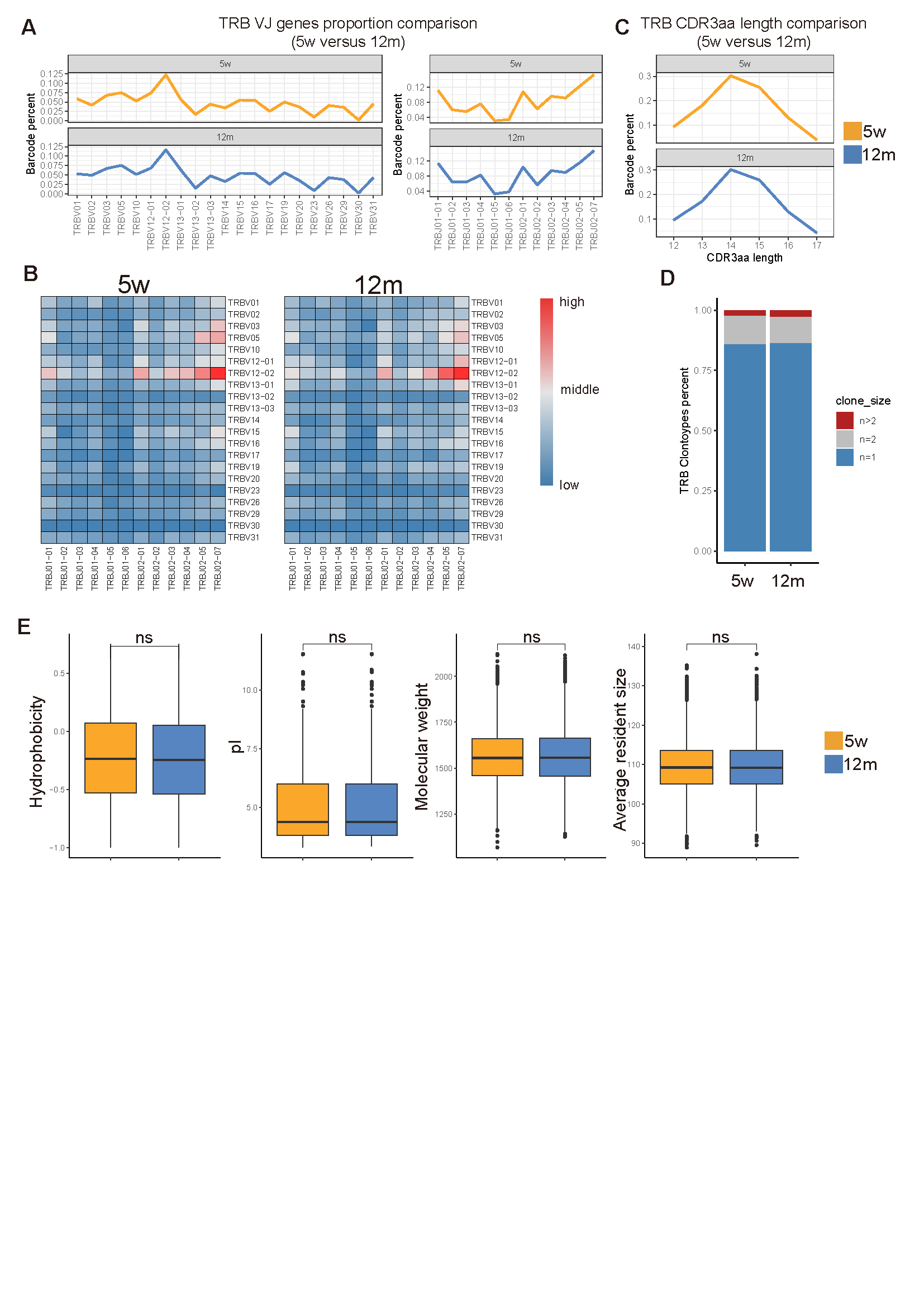


## Figure S19. The difference in TCR immune repertoire in distinct samples of distinct ages in the scTCR-seq data

1. Line plots indicating the TRB VJ genes usage in 5w and 12m thymus samples.
2. Heat maps indicating the TRB VJ gene pairing patterns in 5w and 12m thymus samples.
3. The line plot indicating the distribution of TRB CDR3aa length in 5w and 12m thymus samples.
4. The stacked bar plot indicating the clonal expansion of TRB TCR in 5w and 12m thymus samples.
5. Box plots indicating overall physicochemical features of TRB CDR3aa in 5w and 12m thymus samples. Significances were calculated with the t-test method (ns: p>0.05; *: p<0.05; **: p<0.01; ***: p<0.001; ****: p<0.0001).

## Figure S20


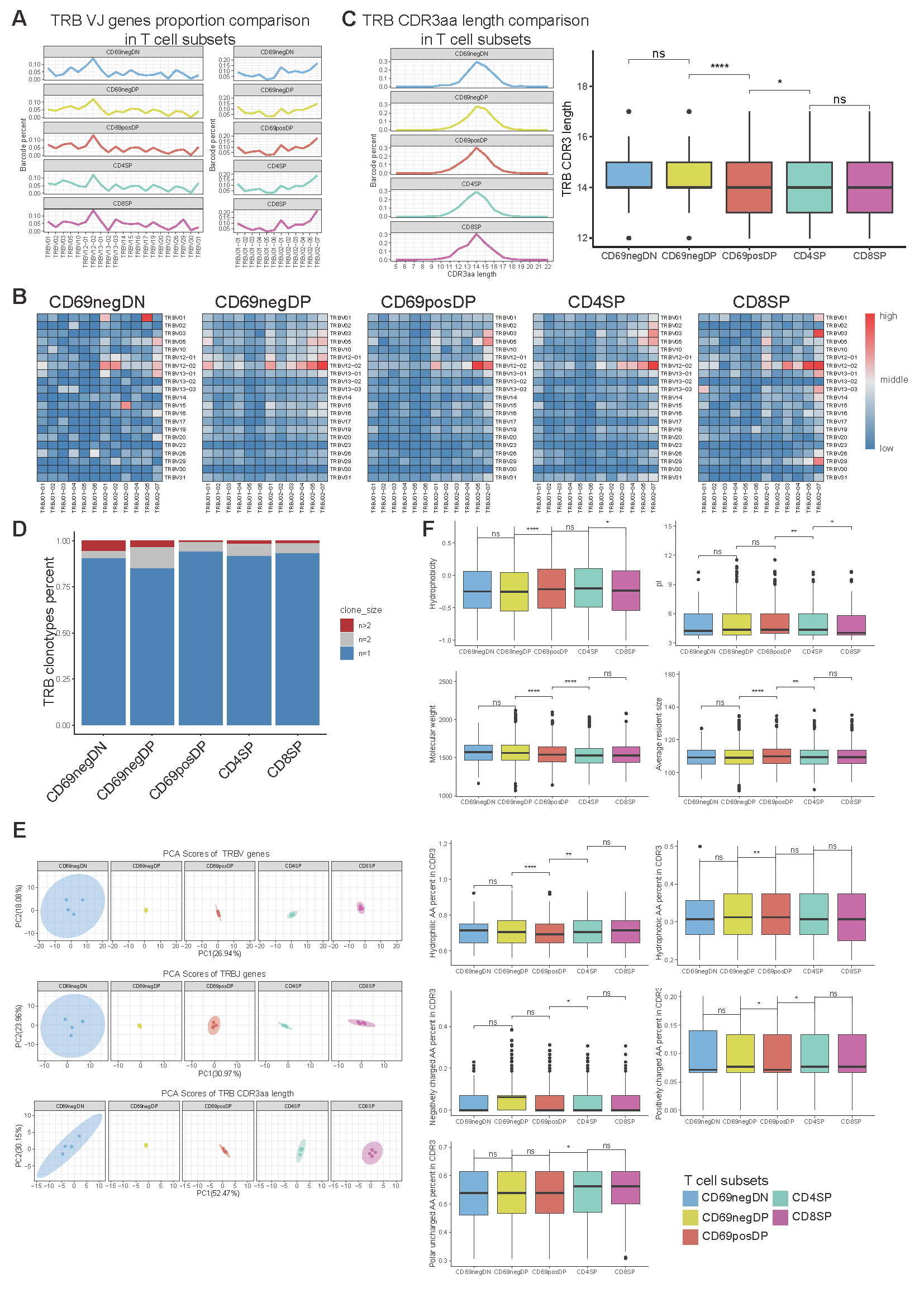


## Figure S20. The difference in the TCR immune repertoire of T cell subsets in the scTCR-seq data

1. Line plots indicating the TRB VJ genes usage in distinct T cell subsets.
2. Heat maps indicating the TRB VJ gene pairing patterns in distinct T cell subsets.
3. The line plot and the box plot indicating the distribution of TRB CDR3aa length in distinct T cell subsets. Significances were calculated with the t-test method (ns: p>0.05; *: p<0.05; **: p<0.01; ***: p<0.001; ****: p<0.0001).
4. The stacked bar plot indicating the clonal expansion of TRB TCR in distinct T cell subsets.
5. Scatter plots indicating the PCA reduction of barcodes based on the distribution of VJ gene usage, and CDR3aa length in distinct T cell subsets.
6. Box plots indicating overall physicochemical features and percent of amino acids of TRB CDR3aa in distinct T cell subsets. Significances were calculated with the t-test method (ns: p>0.05; *: p<0.05; **: p<0.01; ***: p<0.001; ****: p<0.0001).

## Figure S21


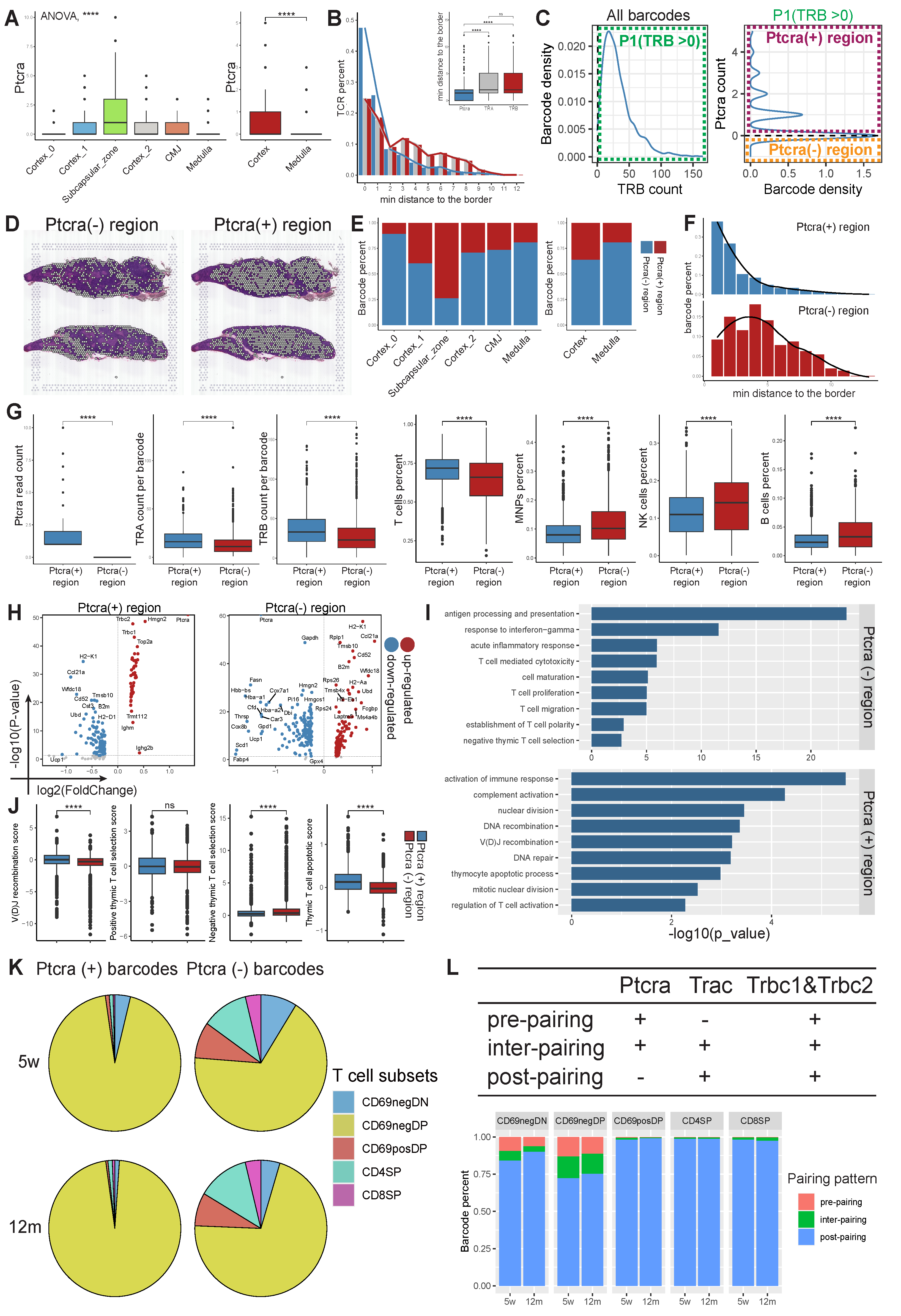


## Figure S21. Spatial, single-cell, and transcriptomic features of the TCR pairing process in the spatial-TCR-seq data and scTCR-seq data

1. Box plots indicating the expression of the pre-TCRα locus in distinct sub-structures. The cortex of the right part means the combination of cortex_0, cortex_1, and cortex_2. Significances were calculated with the t-test and anova methods (ns: p>0.05; *: p<0.05; **: p<0.01; ***: p<0.001; ****: p<0.0001).
2. The bar plot, line plot (left), and box plot (right) of the tissue depth of the pre-TCRα locus, TCRα locus, and TCRβ locus. Significances were calculated with the t-test method (ns: p>0.05; *: p<0.05; **: p<0.01; ***: p<0.001; ****: p<0.0001).
3. Line plots indicating the concept the the Ptcra (-) region and the Ptcra (+) region.
4. Spatial feature plots indicating the distribution of the Ptcra (-) region and the Ptcra (+) region.
5. Bar plots of the distribution of both the Ptcra (-) region and the Ptcra (+) region in sub-structures and the medulla and the cortex region. The cortex of the right part means the combination of cortex_0, cortex_1, and cortex_2.
6. The bar plot of the distance to the border of the thymus of both the Ptcra (-) region and the Ptcra (+) region which indicated the tissue depth.
7. Left, box plots of pre-TCRα locus, TCRα locus, and TCRβ locus count in both the Ptcra (-) region and the Ptcra (+) region. Right, box plots of predicated cell percent in both the Ptcra (-) region and the Ptcra (+) region. Significances were calculated with the t-test method (ns: p>0.05; *: p<0.05; **: p<0.01; ***: p<0.001; ****: p<0.0001).
8. Scatter plots of differentiated genes of both the Ptcra (-) region and the Ptcra (+) region.
9. Bar plots indicating enriched signatures of both the Ptcra (-) region and the Ptcra (+) region. Differential genes (p_val < 0.05 and log2foldchange >0) were used to perform the gene ontology analysis.
10. Box plots indicating the the expression of several signatures in both the Ptcra (-) region and the Ptcra (+) region. Significances were calculated with the t-test method (ns: p>0.05; *: p<0.05; **: p<0.01; ***: p<0.001; ****: p<0.0001).
11. Pie plots indicating the proportion of T cell subsets detecting the pre-TCRα locus during aging in the scTCR-seq data.
12. Top, the annotation of distinct barcodes including pre-pairing, inter-pairing, and post-pairing based on the expression of distinct kinds of TCR locus in the scTCR-seq data. Bottom, the stacked bar plot indicating the proportion of distinct pairing patterns among T cell subsets in the scTCR-seq data.

## Figure S22


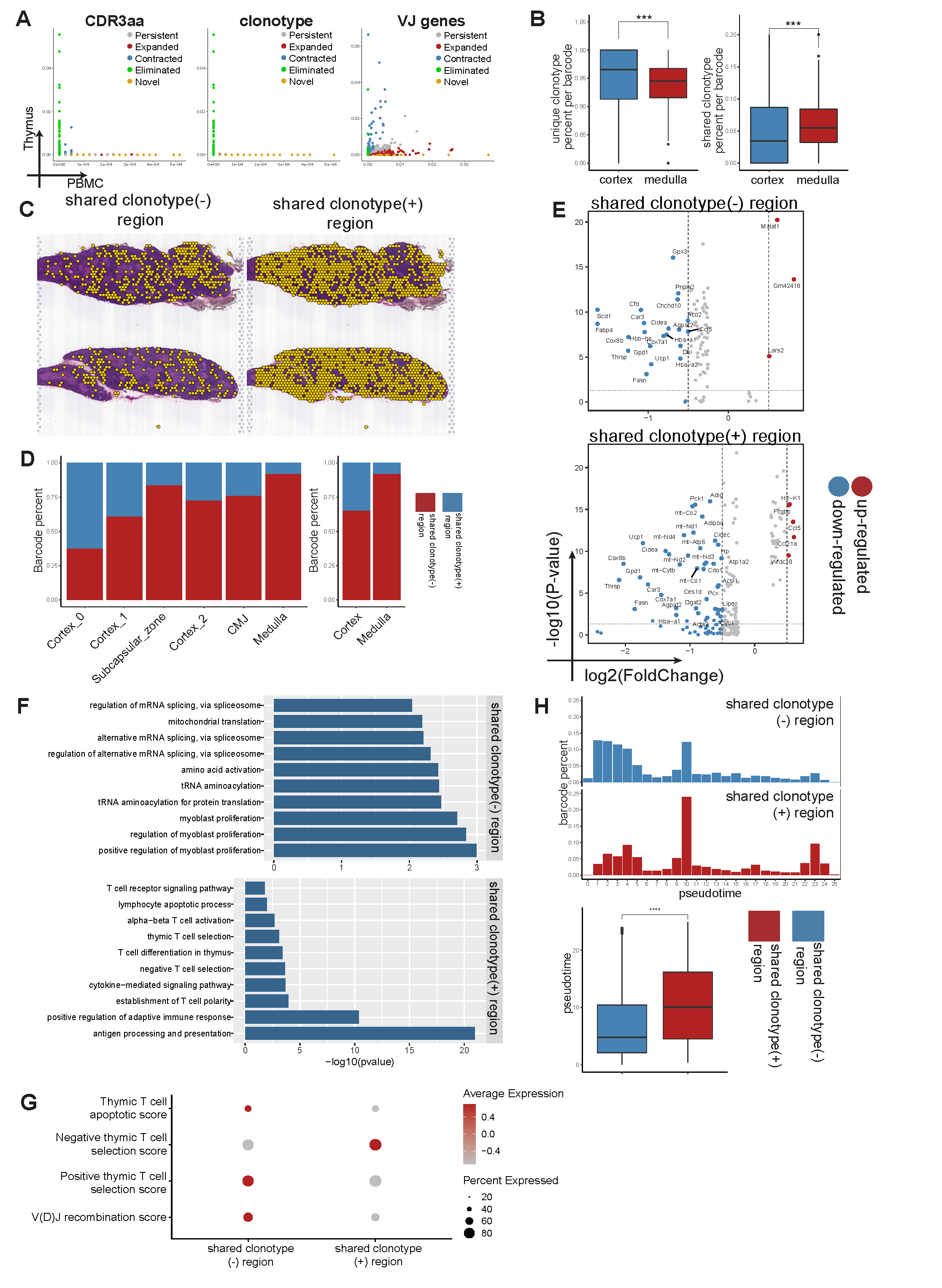


## Figure S22. The transcriptional and spatial features of the thymic TCR maturation process in the spatial-TCR-seq data

1. Scatter plots and the correlation of the frequency of CDR3 (left), clonotypes (middle), and VJ gene pairing (right) between the thymus sample and the PBMC sample. Expanded means the percent of clonotypes in the PBMC was two times higher than the percent in the thymus. Contracted means the percent of clonotypes in the thymus was two times higher than the percent in the PBMC.
2. Box plots of the frequency of unique clonotypes and shared clonotypes per barcode between the medulla region and the cortex region. The cortex means the combination of cortex_0, cortex_1, and cortex_2. Significances were calculated with the t-test method (ns: p>0.05; *: p<0.05; **: p<0.01; ***: p<0.001; ****: p<0.0001).
3. Spatial distribution of the shared clonotype (-) region and the shared clonotype (+) region.
4. Bar plots of the distribution of both the shared clonotype (-) region and the shared clonotype (+) region in sub-structures (left) as well as in the cortex and the medulla (right). The cortex of the right part means the combination of cortex_0, cortex_1, and cortex_2.
5. Scatter plots of differentiated genes of both the shared clonotype (-) region and the shared clonotype (+) region.
6. bar plots of enriched signature pathways of both the shared clonotype (-) region and the shared clonotype (+) region. Differential genes (p_val < 0.05 and log2foldchange >0) were used to perform the gene ontology analysis.
7. The dot plot of the score of several vital signature pathways in both the shared clonotype (-) region and the shared clonotype (+) region.
8. The bar plot (top) and the box plot (bottom) of the correlation between the pseudo-time trajectory and the two regions mentioned above. Significances were calculated with the t-test method (ns: p>0.05; *: p<0.05; **: p<0.01; ***: p<0.001; ****: p<0.0001).

## Figure S23


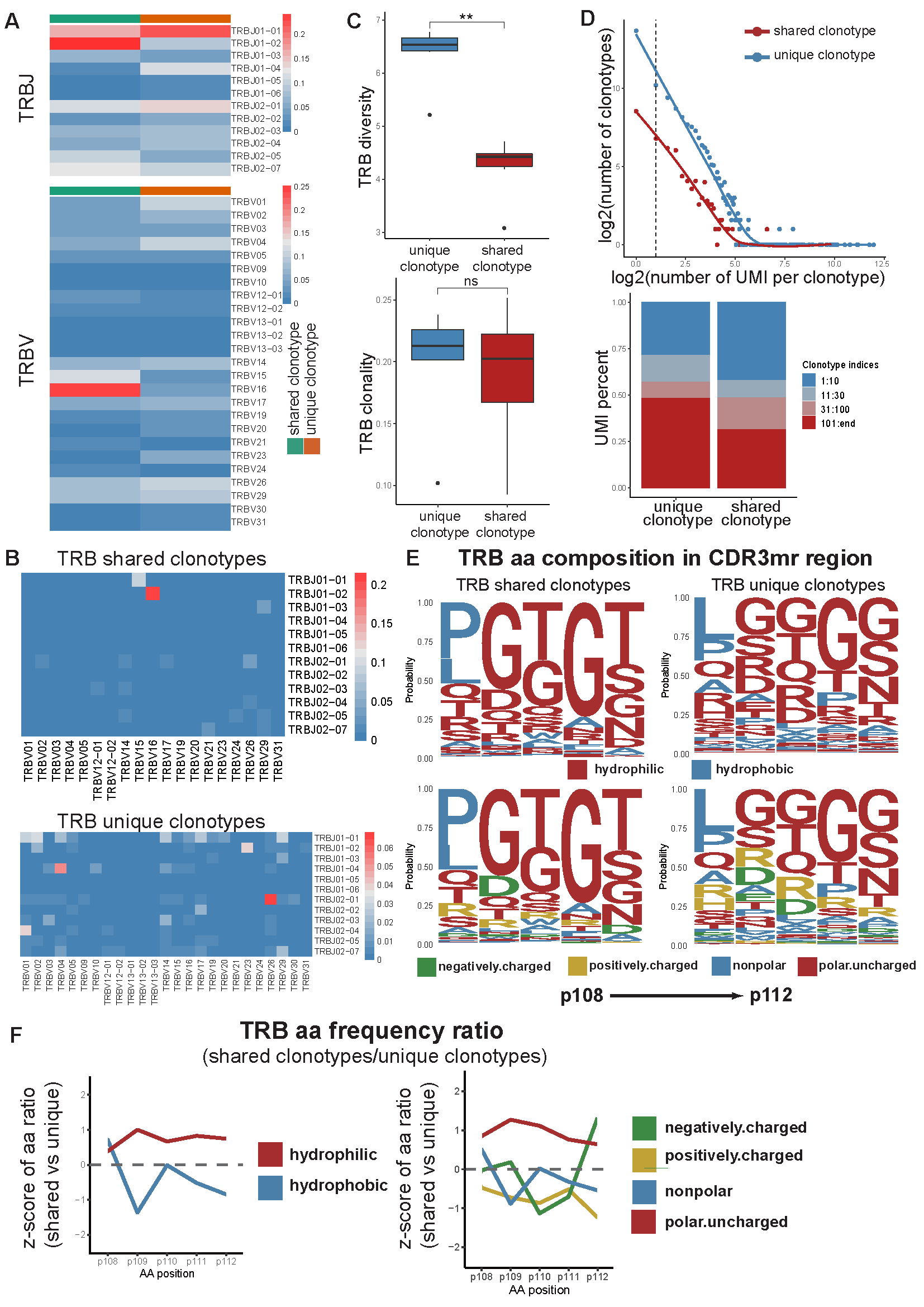


## Figure S23. Analyses of the immune repertoire of the thymic TCR maturation process in the spatial-TCR-seq data

1. TRB VJ gene usage between shared clonotypes that were detected both in the thymus and PBMC and unique clonotypes that were detected only in the thymus.
2. The frequency of TRB VJ pairing patterns of both kinds of clonotypes.
3. Boxplots of diversity and clonality of both unique clonotypes and shared clonotypes of TCRβ locus. Significances were calculated with the t-test method (ns: p>0.05; *: p<0.05; **: p<0.01; ***: p<0.001; ****: p<0.0001).
4. The scatter plot (top) and the bar plot (bottom) of the proportion of clonotypes of distinct clone sizes by unique clonotypes and shared clonotypes.
5. Sequence logo plots of TRB CDR3 middle region between shared clonotypes (left) and unique clonotypes (right) of the TCRβ locus by the physicochemical features of the residues.
6. Line plots of the frequency comparison of TRB amino acids grouped by distinct physicochemical features. Z-score > 0 means amino acids were more enriched in shared clonotypes.

## Figure S24


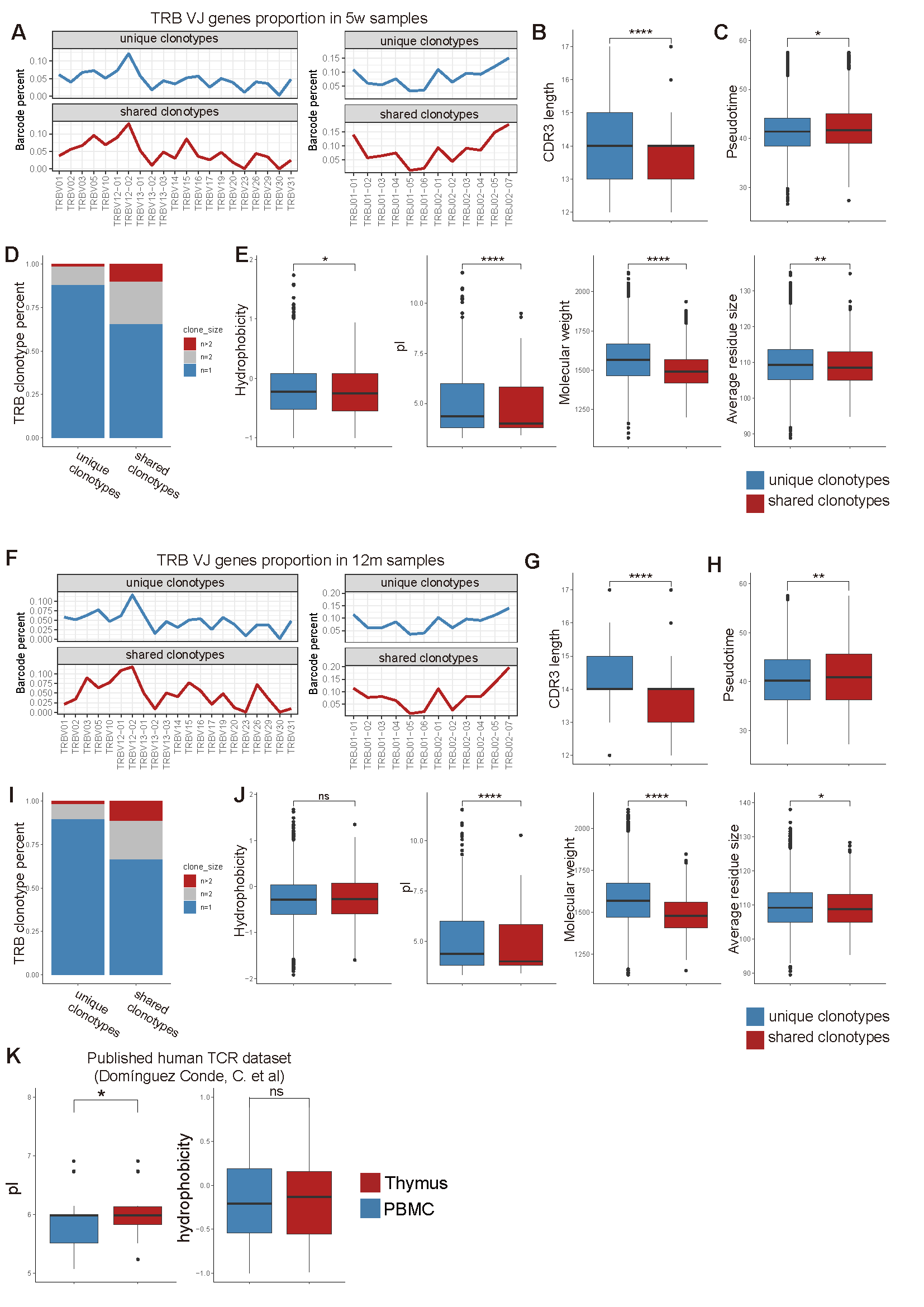


## Figure S24. Analyses of the immune repertoire of the thymic TCR maturation process in the scTCR-seq data

1. Line plots indicating the TRB VJ gene usage between shared clonotypes that were detected both in the 5w thymus and PBMC and unique clonotypes that were detected only in the 5w thymus in the scTCR-seq data.
2. The box plot indicating the TRB CDR3aa length between unique clonotypes and shared clonotypes of the 5w samples in the scTCR-seq data. Significances were calculated with the t-test method (ns: p>0.05; *: p<0.05; **: p<0.01; ***: p<0.001; ****: p<0.0001).
3. The box plot indicating the pseudo-time trajectory of TRB CDR3aa between unique clonotypes and shared clonotypes of the 5w samples in the scTCR-seq data. Significances were calculated with the t-test method (ns: p>0.05; *: p<0.05; **: p<0.01; ***: p<0.001; ****: p<0.0001).
4. The bar plot indicating the clonal expansion of TRB CDR3aa between unique clonotypes and shared clonotypes of the 5w samples in the scTCR-seq data.
5. The box plot indicating physicochemical features of TRB CDR3aa between unique clonotypes and shared clonotypes of the 5w samples in the scTCR-seq data. Significances were calculated with the t-test method (ns: p>0.05; *: p<0.05; **: p<0.01; ***: p<0.001; ****: p<0.0001).
6. Line plots indicating the TRB VJ gene usage between shared clonotypes that were detected both in the 12m thymus and PBMC and unique clonotypes that were detected only in the 12m thymus in the scTCR-seq data.
7. The box plot indicating the TRB CDR3aa length between unique clonotypes and shared clonotypes of the 12m samples in the scTCR-seq data. Significances were calculated with the t-test method (ns: p>0.05; *: p<0.05; **: p<0.01; ***: p<0.001; ****: p<0.0001).
8. The box plot indicating the pseudo-time trajectory of TRB CDR3aa between unique clonotypes and shared clonotypes of the 12m samples in the scTCR-seq data. Significances were calculated with the t-test method (ns: p>0.05; *: p<0.05; **: p<0.01; ***: p<0.001; ****: p<0.0001).
9. The bar plot indicating the clonal expansion of TRB CDR3aa between unique clonotypes and shared clonotypes of the 12m samples in the scTCR-seq data.
10. The box plot indicating physicochemical features of TRB CDR3aa between unique clonotypes and shared clonotypes of the 12m samples in the scTCR-seq data. Significances were calculated with the t-test method (ns: p>0.05; *: p<0.05; **: p<0.01; ***: p<0.001; ****: p<0.0001).
11. Box plots of the TRB CDR3aa frequency of distinct physicochemical properties mentioned above derived from published human-derived scTCR-seq dataset data by thymus and PBMC clonotypes. A T-test was utilized to calculate the statistical significance (ns: p>0.05; *: p<0.05; **: p<0.01; ***: p<0.001; ****: p<0.0001).

## Figure S25


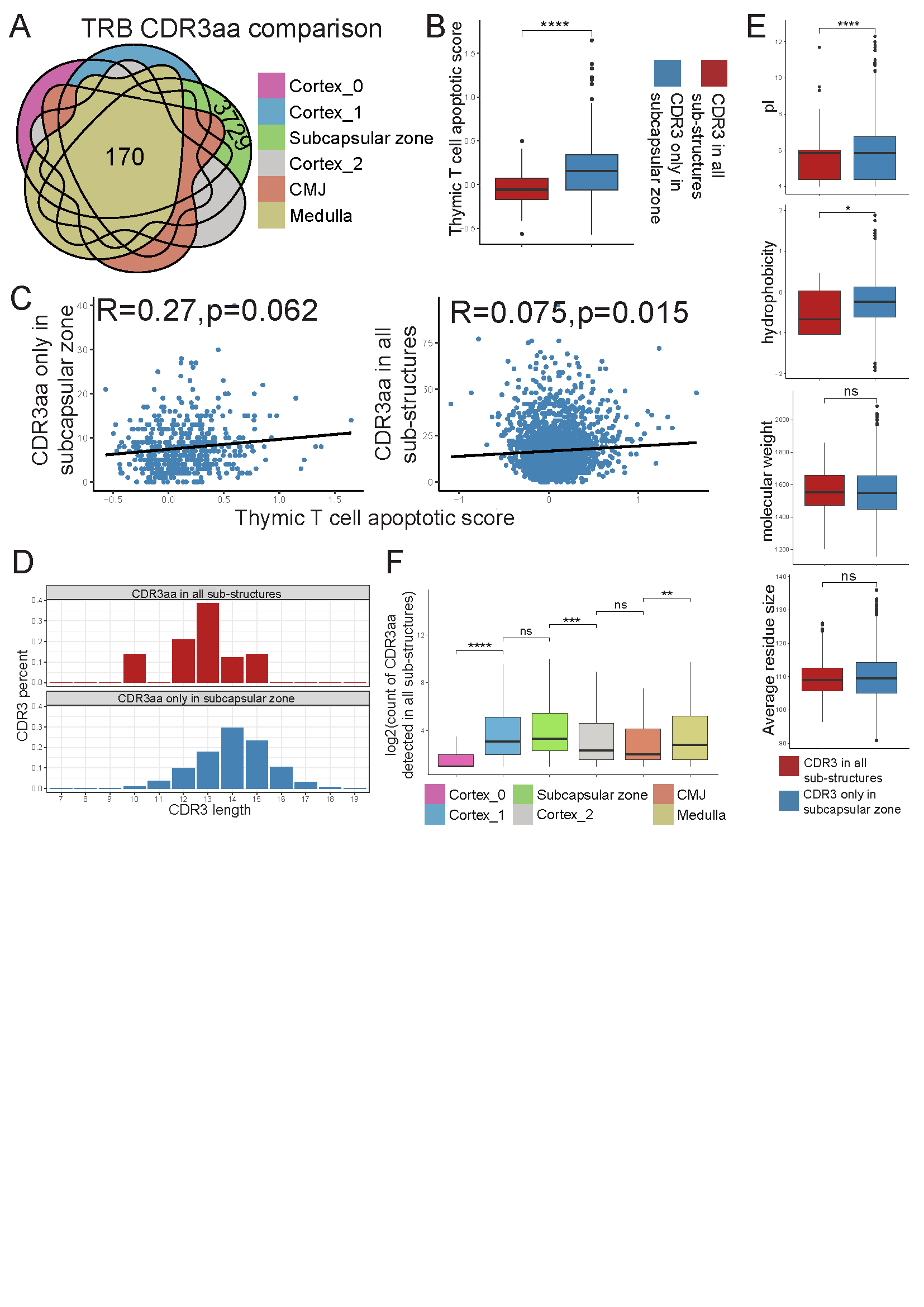


## Figure S25. Differences of mature clonotypes and apoptotic clonotypes revealed by the comparison of the immune repertoire among thymus sub-structures in the ST-seq dataset.

1. The venn diagram indicating the difference of the TRB CDR3aa among thymus sub-structures in the spatial-TCR-seq data. Numbers indicating the number of unique TRB CDR3aa count.
2. The box plot indicating the expression of the T cell apoptotic process signature between clonotypes detected in all sub-structures and clonotypes only detected in the subcapsular zone in the spatial-TCR-seq data. Significances were calculated with the t-test method (ns: p>0.05; *: p<0.05; **: p<0.01; ***: p<0.001; ****: p<0.0001).
3. The scatter plot indicating the correlation between the count of distinct clonotype types and the expression of the T cell apoptotic process signature in the spatial-TCR-seq data. The pearson was used to calculate the correlation. The t.test was used to calculate the significance.
4. The bar plot indicating the TRB CDR3aa length between distinct clonotypes in the spatial-TCR-seq data.
5. The box plot indicating the physicochemical features of TRB CDR3aa between distinct clonotypes in the spatial-TCR-seq data. Significances were calculated with the t-test method (ns: p>0.05; *: p<0.05; **: p<0.01; ***: p<0.001; ****: p<0.0001).
6. The box plot indicating the dynamic of the count of clonotypes detected in all thymus sub-structures in the spatial-TCR-seq data. Significances were calculated with the t-test method (ns: p>0.05; *: p<0.05; **: p<0.01; ***: p<0.001; ****: p<0.0001).

## Figure S26


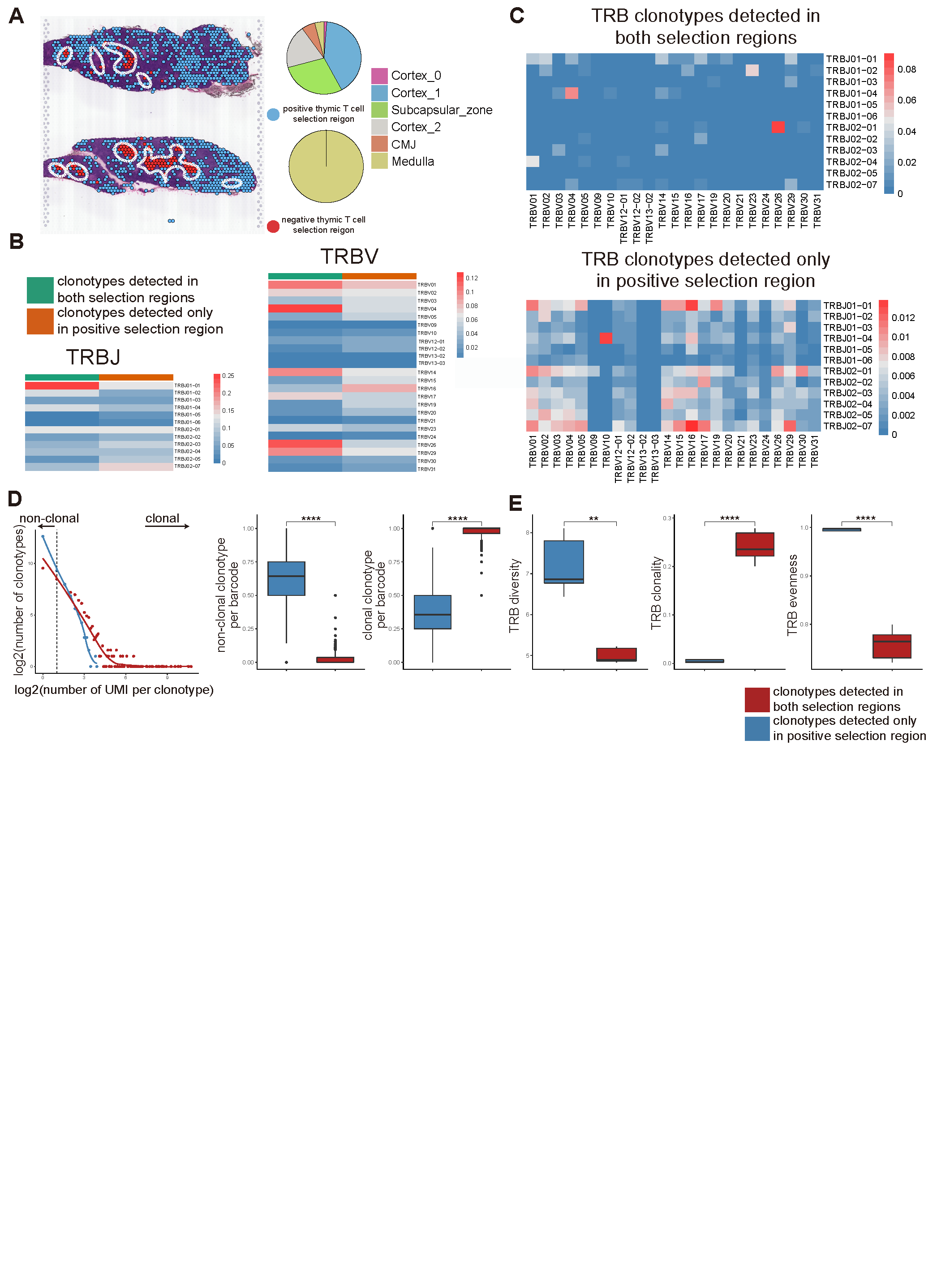


## Figure S26. The overall features of the dynamic of TRB VJ gene usage upon the thymic positive selection in the spatial-TCR-seq data

1. The spatial distribution of the negative thymic T cell selection region and the positive thymic T cell selection region is calculated by the K-means algorithm.
2. Heatmap of TCR VJ genes usage of the TCRβ locus between both kinds of clonotypes that were detected in both selection regions or only in the positive selection region.
3. Heatmap of TCR pairing pattern frequency between kinds of clonotypes of the TCRβ locus.
4. Left, the scatter plot of the proportion of TRB clonotypes of distinct clone sizes by passed clonotypes and unpassed clonotypes. Right, boxplots of the percent of clonal clonotypes and nonclonal clonotypes per barcode between passed and unpassed clonotypes. Significances were calculated with the t-test method (ns: p>0.05; *: p<0.05; **: p<0.01; ***: p<0.001; ****: p<0.0001).
5. Boxplots of diversity, clonality, and evenness of both passed clonotypes and unpassed clonotypes of TCRβ locus under the thymic positive selection process. Significances were calculated with the t-test method (ns: p>0.05; *: p<0.05; **: p<0.01; ***: p<0.001; ****: p<0.0001).

## Figure S27


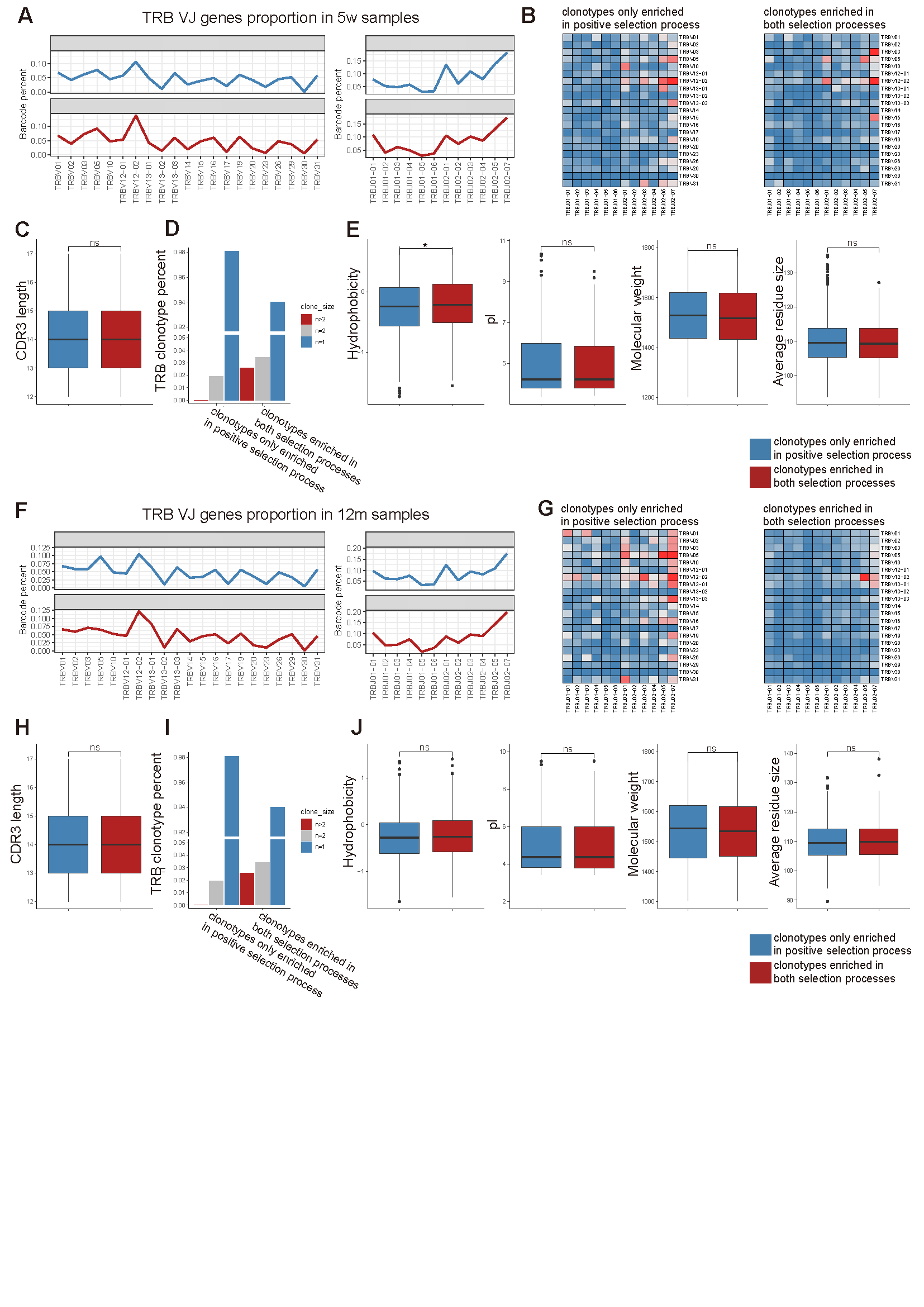


## Figure S27. The overall features of the dynamic of the immune repertoire upon the thymic positive selection in the scTCR-seq data

1. Line plots indicating the TRB VJ gene usage between shared clonotypes that were enriched both in the positive selection and the negative selection and unique clonotypes that were enriched only in the positive selection of the 5w samples in the scTCR-seq data.
2. Heat maps indicating the difference in TRB VJ gene pairing pattern between distinct kinds of clonotypes of the 5w samples in the scTCR-seq data.
3. The box plot indicating the TRB CDR3aa length between distinct kinds of clonotypes of the 5w samples in the scTCR-seq data. Significances were calculated with the t-test method (ns: p>0.05; *: p<0.05; **: p<0.01; ***: p<0.001; ****: p<0.0001).
4. The bar plot indicating the clonal expansion of TRB CDR3aa between distinct kinds of clonotypes of the 5w samples in the scTCR-seq data.
5. The box plot indicating physicochemical features of TRB CDR3aa between distinct kinds of clonotypes of the 5w samples in the scTCR-seq data. Significances were calculated with the t-test method (ns: p>0.05; *: p<0.05; **: p<0.01; ***: p<0.001; ****: p<0.0001).
6. Line plots indicating the TRB VJ gene usage between shared clonotypes that were enriched both in the positive selection and the negative selection and unique clonotypes that were enriched only in the positive selection of the 12m samples in the scTCR-seq data.
7. Heat maps indicating the difference in TRB VJ gene pairing pattern between distinct kinds of clonotypes of the 12m samples in the scTCR-seq data.
8. The box plot indicating the TRB CDR3aa length between distinct kinds of clonotypes of the 12m samples in the scTCR-seq data. Significances were calculated with the t-test method (ns: p>0.05; *: p<0.05; **: p<0.01; ***: p<0.001; ****: p<0.0001).
9. The bar plot indicating the clonal expansion of TRB CDR3aa between distinct kinds of clonotypes of the 12m samples in the scTCR-seq data.
10. The box plot indicating physicochemical features of TRB CDR3aa between distinct kinds of clonotypes of the 12m samples in the scTCR-seq data. Significances were calculated with the t-test method (ns: p>0.05; *: p<0.05; **: p<0.01; ***: p<0.001; ****: p<0.0001).

## Figure S28


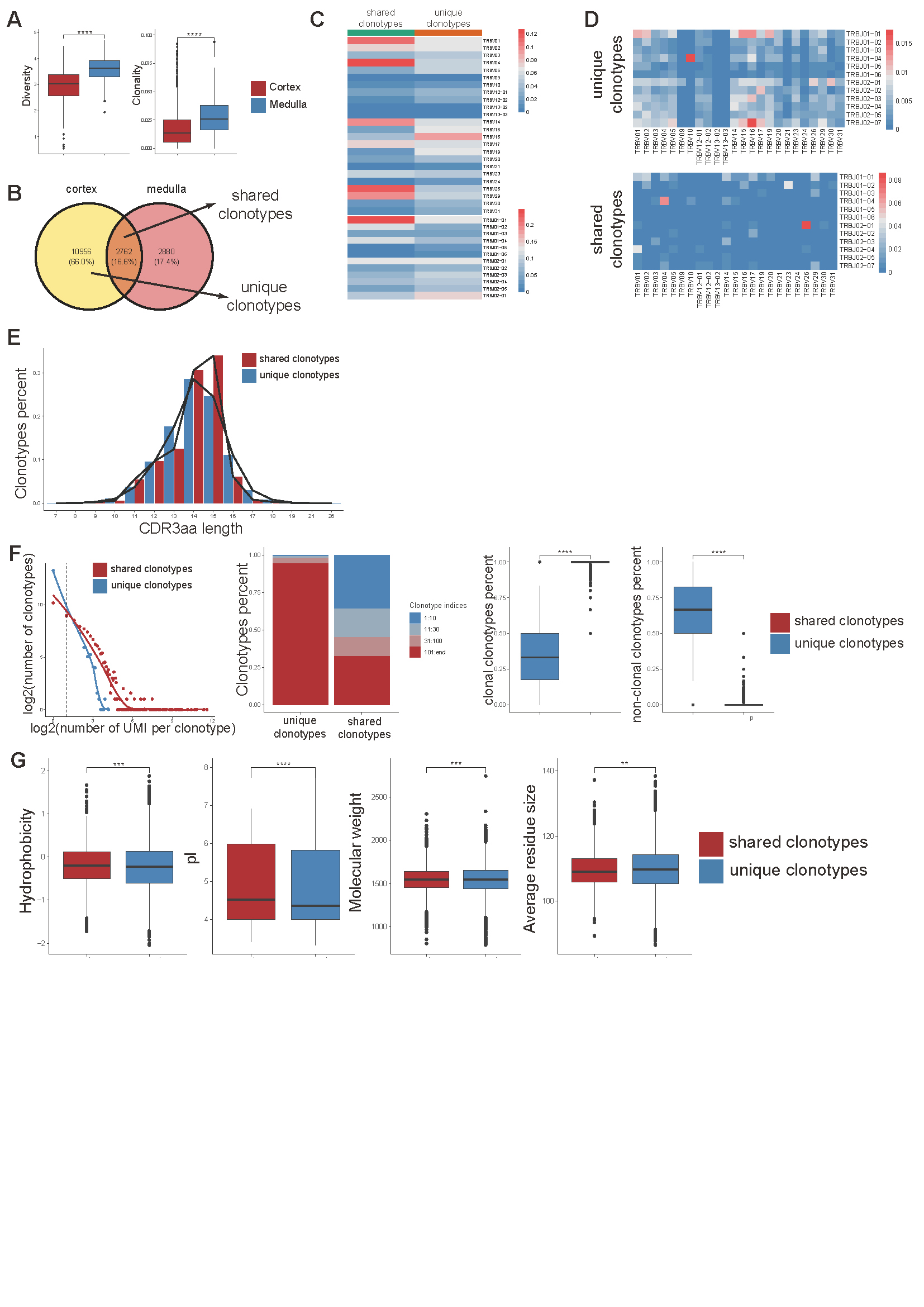


## Figure S28. The difference in TCR immune repertoire between the cortex and the medulla in the spatial-TCR-seq data

1. Boxplots of diversity and clonality of TRB clonotypes between the cortex and the medulla. Significances were calculated with the t-test method (ns: p>0.05; *: p<0.05; **: p<0.01; ***: p<0.001; ****: p<0.0001).
2. The schematic diagram of the definition of clonotypes of TRB clonotypes that were unique in the cortex or shared between the cortex and the medulla.
3. VJ gene usage between shared clonotypes that were detected both in the cortex and the medulla and unique clonotypes that were detected only in the thymus.
4. The frequency of VJ pairing patterns of both kinds of clonotypes.
5. The bar plot indicating the distribution of TRB CDR3aa length in both kinds of clonotypes.
6. The line plot, the bar plot, and the box plot indicating the difference in clonal expansion of TRB clonotypes in both kinds of clonotypes. Significances were calculated with the t-test method (ns: p>0.05; *: p<0.05; **: p<0.01; ***: p<0.001; ****: p<0.0001).
7. Box plots indicating overall physicochemical features between both kinds of clonotypes. Significances were calculated with the t-test method (ns: p>0.05; *: p<0.05; **: p<0.01; ***: p<0.001; ****: p<0.0001).

## Figure S29


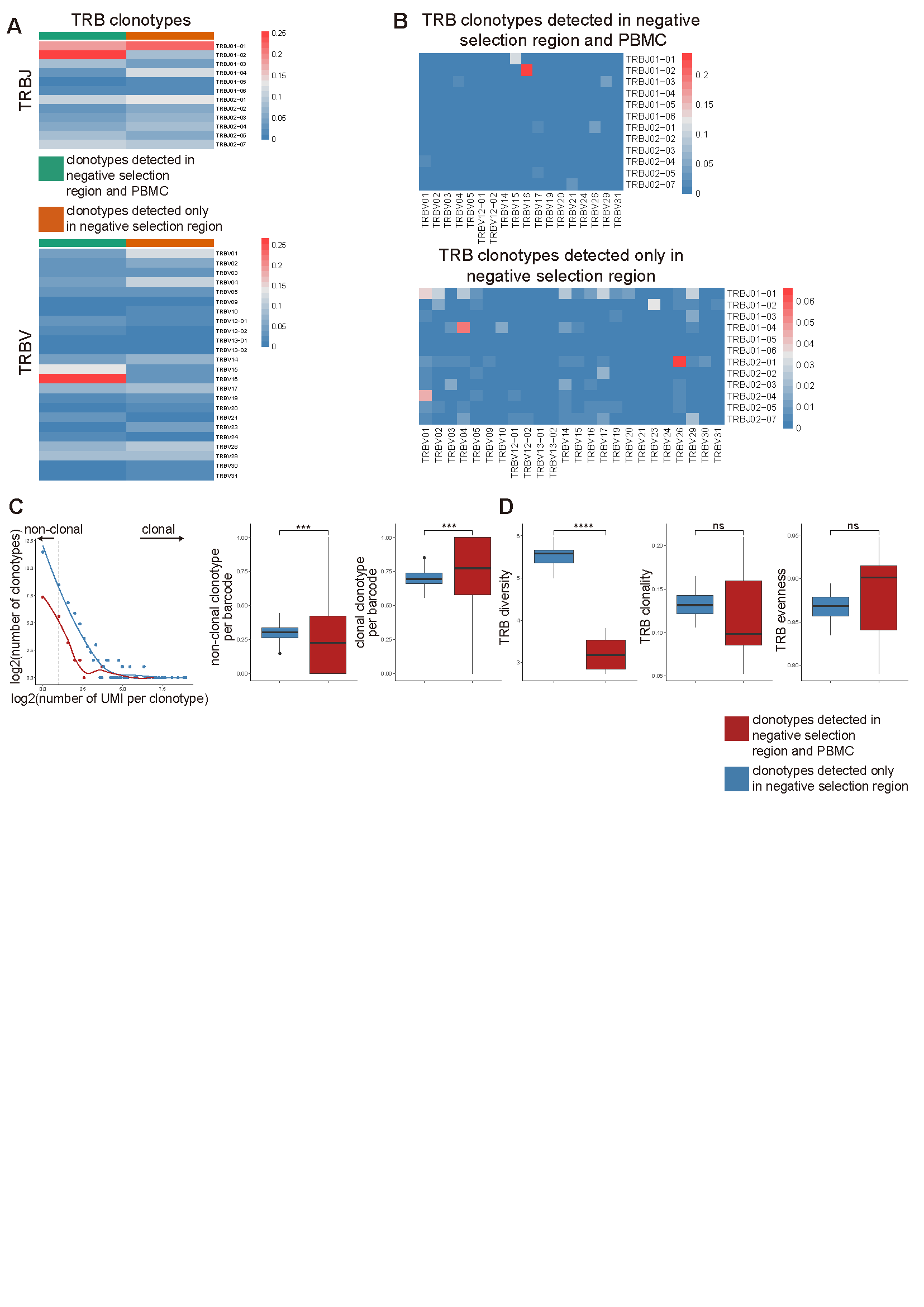


## Figure S29. The overall features of the dynamic of TRB VJ gene usage upon the thymic negative selection in the spatial-TCR-seq data

1. Heatmap of TCR VJ genes usage of the TCRβ locus between both kinds of clonotypes that were detected in both the negative selection region and PBMC or only in the negative selection region.
2. Heatmap of TCR pairing pattern frequency between kinds of clonotypes of the TCRβ locus.
3. Left, the scatter plot of the proportion of TRB clonotypes of distinct clone sizes by passed clonotypes and unpassed clonotypes. Right, boxplots of the percent of clonal clonotypes and nonclonal clonotypes per barcode between passed and unpassed clonotypes. Significances were calculated with the t-test method (ns: p>0.05; *: p<0.05; **: p<0.01; ***: p<0.001; ****: p<0.0001).
4. Boxplots of diversity, clonality, and evenness of both passed clonotypes and unpassed clonotypes of TCRβ locus under the thymic negative selection process. Significances were calculated with the t-test method (ns: p>0.05; *: p<0.05; **: p<0.01; ***: p<0.001; ****: p<0.0001).

## Figure S30


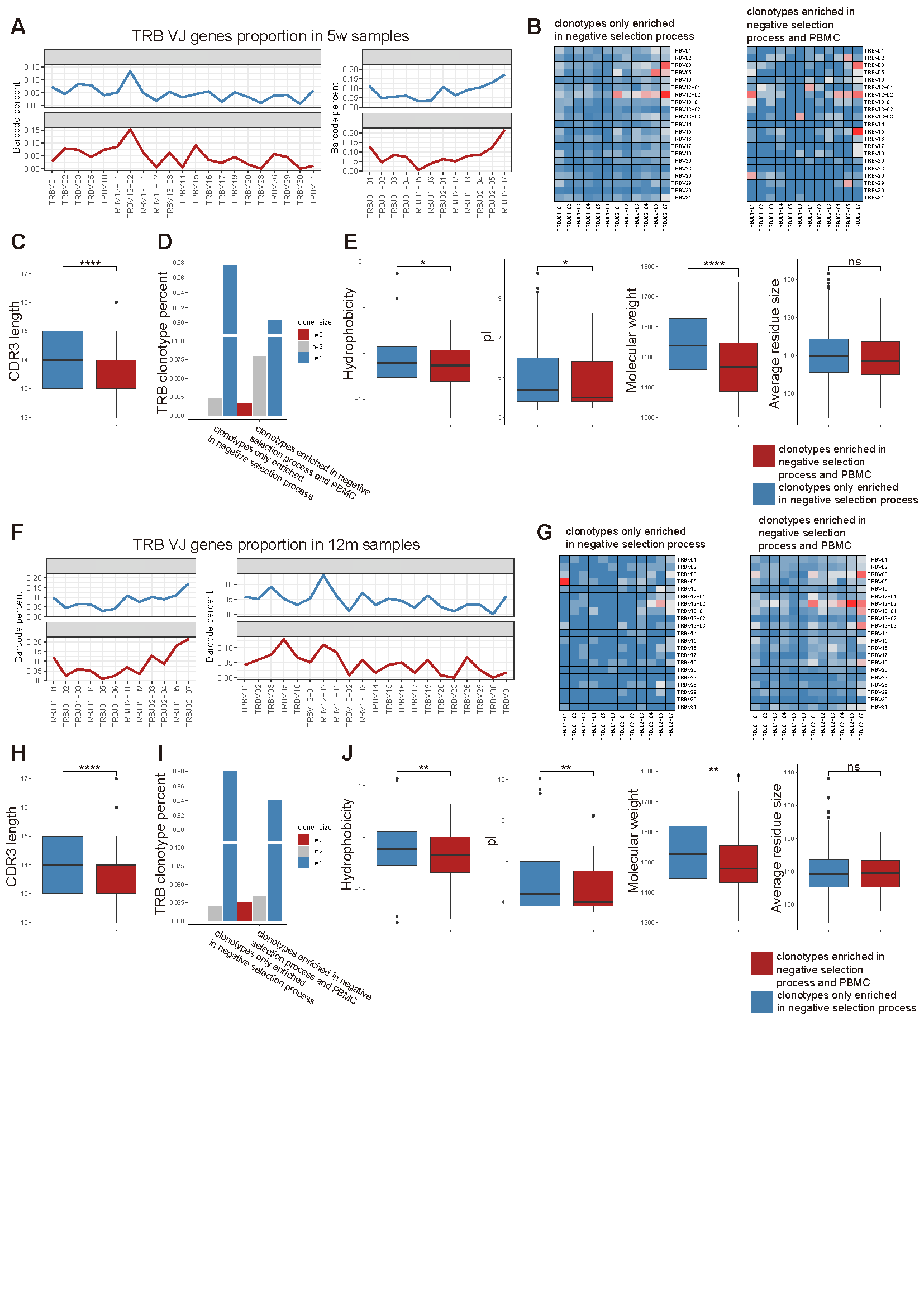


## Figure S30. The overall features of the dynamic of the immune repertoire upon the thymic negative selection in the scTCR-seq data

1. Line plots indicating the TRB VJ gene usage between shared clonotypes that were enriched both in the negative selection and the PBMC and unique clonotypes that were enriched only in the negative selection of the 5w samples in the scTCR-seq data.
2. Heat maps indicating the difference in TRB VJ gene pairing pattern between distinct kinds of clonotypes of the 5w samples in the scTCR-seq data.
3. The box plot indicating the TRB CDR3aa length between distinct kinds of clonotypes of the 5w samples in the scTCR-seq data. Significances were calculated with the t-test method (ns: p>0.05; *: p<0.05; **: p<0.01; ***: p<0.001; ****: p<0.0001).
4. The bar plot indicating the clonal expansion of TRB CDR3aa between distinct kinds of clonotypes of the 5w samples in the scTCR-seq data.
5. The box plot indicating physicochemical features of TRB CDR3aa between distinct kinds of clonotypes of the 5w samples in the scTCR-seq data. Significances were calculated with the t-test method (ns: p>0.05; *: p<0.05; **: p<0.01; ***: p<0.001; ****: p<0.0001).
6. Line plots indicating the TRB VJ gene usage between shared clonotypes that were enriched both in the positive selection and the negative selection and unique clonotypes that were enriched only in the positive selection of the 12m samples in the scTCR-seq data.
7. Heat maps indicating the difference in TRB VJ gene pairing pattern between distinct kinds of clonotypes of the 12m samples in the scTCR-seq data.
8. The box plot indicating the TRB CDR3aa length between distinct kinds of clonotypes of the 12m samples in the scTCR-seq data. Significances were calculated with the t-test method (ns: p>0.05; *: p<0.05; **: p<0.01; ***: p<0.001; ****: p<0.0001).
9. The bar plot indicating the clonal expansion of TRB CDR3aa between distinct kinds of clonotypes of the 12m samples in the scTCR-seq data.
10. The box plot indicating physicochemical features of TRB CDR3aa between distinct kinds of clonotypes of the 12m samples in the scTCR-seq data. Significances were calculated with the t-test method (ns: p>0.05; *: p<0.05; **: p<0.01; ***: p<0.001; ****: p<0.0001).

## Figure S31


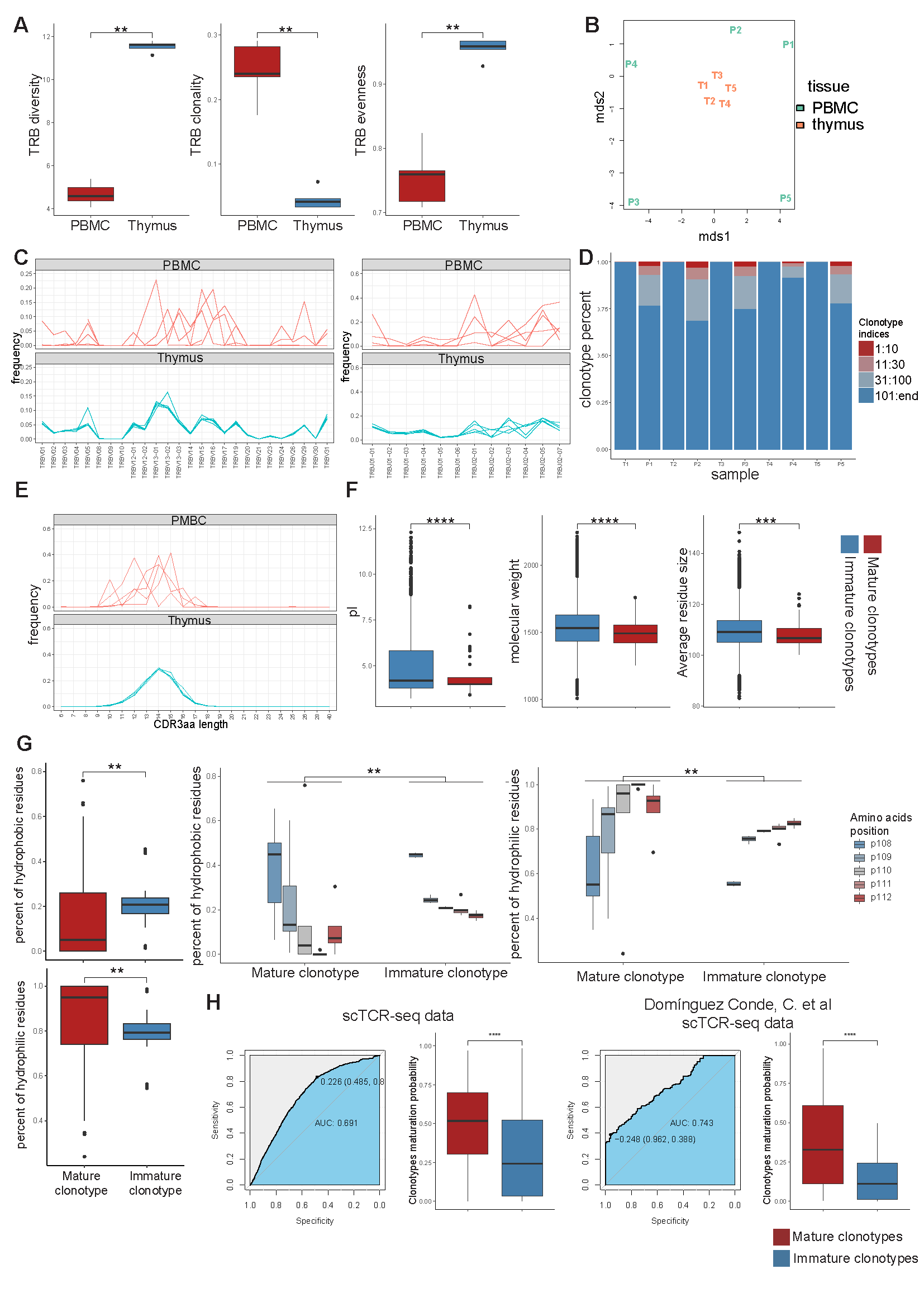


## Figure S31. The comparative analyses of the thymus and the PBMC of the TCR-seq data

1. Box plots of diversity, clonality, and evenness of individual spots derived from the TCR-seq dataset by the thymus samples and the PBMC samples. Significances were calculated with the t-test method (ns: p>0.05; *: p<0.05; **: p<0.01; ***: p<0.001; ****: p<0.0001).
2. The multi-dimensional scaling (MDS) clustering plot of distinct samples.
3. Line charts of TCR VJ genes usage of the TCRβ locus between both kinds of samples.
4. The bar plot of the proportion of clonotypes of distinct clone sizes of distinct samples.
5. The line chart of the proportion of CDR3aa of distinct lengths grouped by both kinds of samples.
6. Boxplots of physicochemical features including pI, molecular weight, and average residue size between mature clonotypes and immature clonotypes. Significances were calculated with the t-test method (ns: p>0.05; *: p<0.05; **: p<0.01; ***: p<0.001; ****: p<0.0001).
7. Left, boxplots of the frequency of residues of distinct physicochemical features between mature clonotypes and immature clonotypes in the TCR-seq dataset. Right, boxplots of the frequency of residues of distinct physicochemical features per position between mature clonotypes and immature clonotypes in the TCR-seq dataset. Significances were calculated with the t-test method (ns: p>0.05; *: p<0.05; **: p<0.01; ***: p<0.001; ****: p<0.0001).
8. The ROC curve of the scTCR-seq datasets estimates the specificity and sensitivity of the logistic regression model. The box plot indicating the maturation probability between mature clonotypes and immature clonotypes predicated by the logistic regression model.

## Figure S32


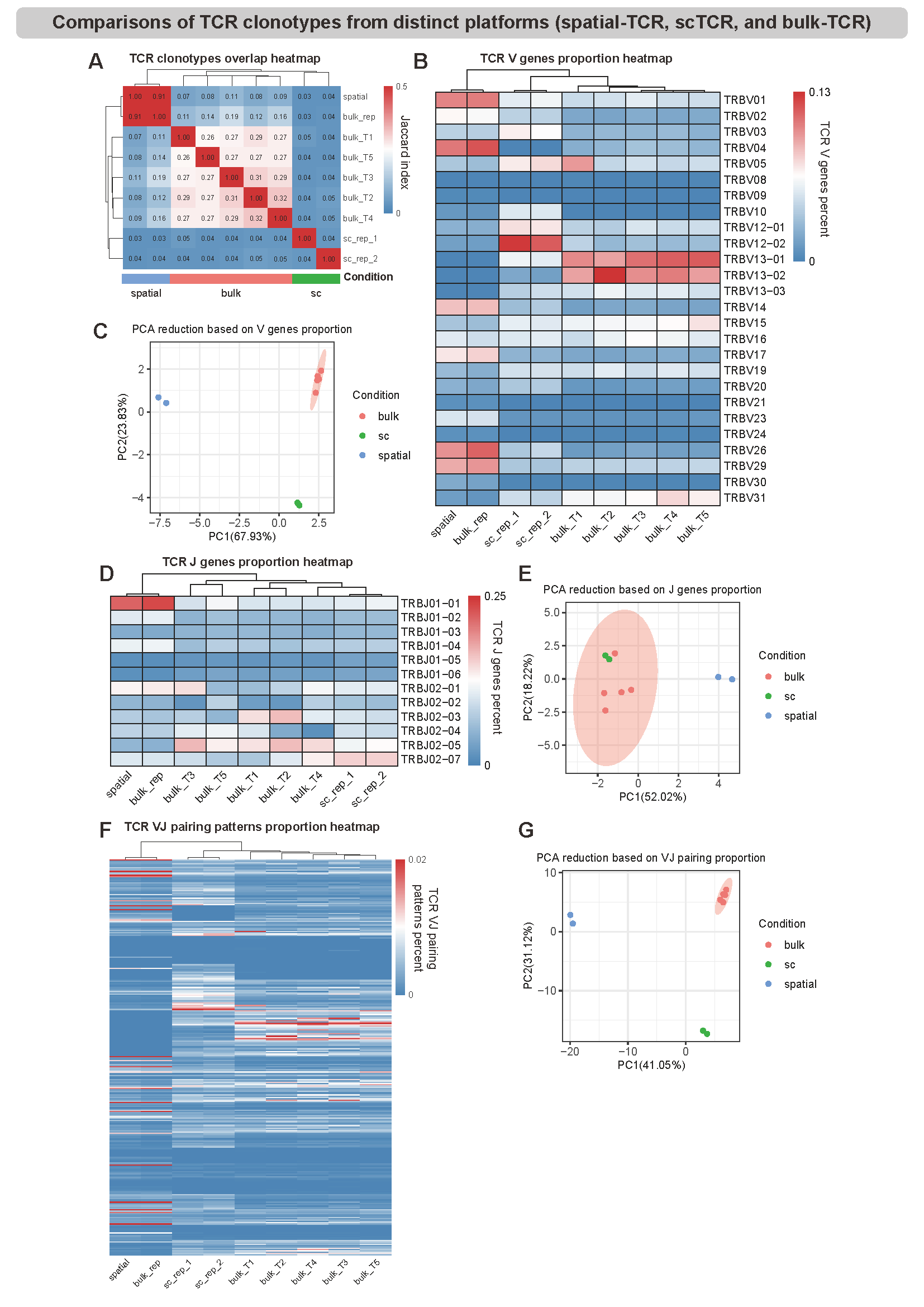


## Figure S32. The comparative analyses of TCR clonotypes among distinct samples and distinct sequencing platforms

1. Heatmaps showing the proportion of overlapped TCR clonotypes among samples of distinct TCR sequencing platforms. The Jaccard index was used to indicate the similarity. The Jaccard Index measures the similarity between two sets as the size of their intersection divided by the size of their union.
2. The heatmap showing the proportion of TCR V genes among samples of distinct TCR sequencing platforms.
3. The PCA visualization of samples based on V genes proportion profiles. The first two principal components explain PC1 and PC2 of the total variance. Samples were colored by data modality. Confidence ellipses were shown for each modality.
4. The heatmap showing the proportion of TCR J genes among samples of distinct TCR sequencing platforms.
5. The PCA visualization of samples based on J genes proportion profiles. The first two principal components explain PC1 and PC2 of the total variance. Samples were colored by data modality. Confidence ellipses were shown for each modality.
6. The heatmap showing the proportion of TCR VJ genes pairing patterns among samples of distinct TCR sequencing platforms.
7. The PCA visualization of samples based on VJ genes pairing patterns proportion profiles. The first two principal components explain PC1 and PC2 of the total variance. Samples were colored by data modality. Confidence ellipses were shown for each modality.

## Figure S33


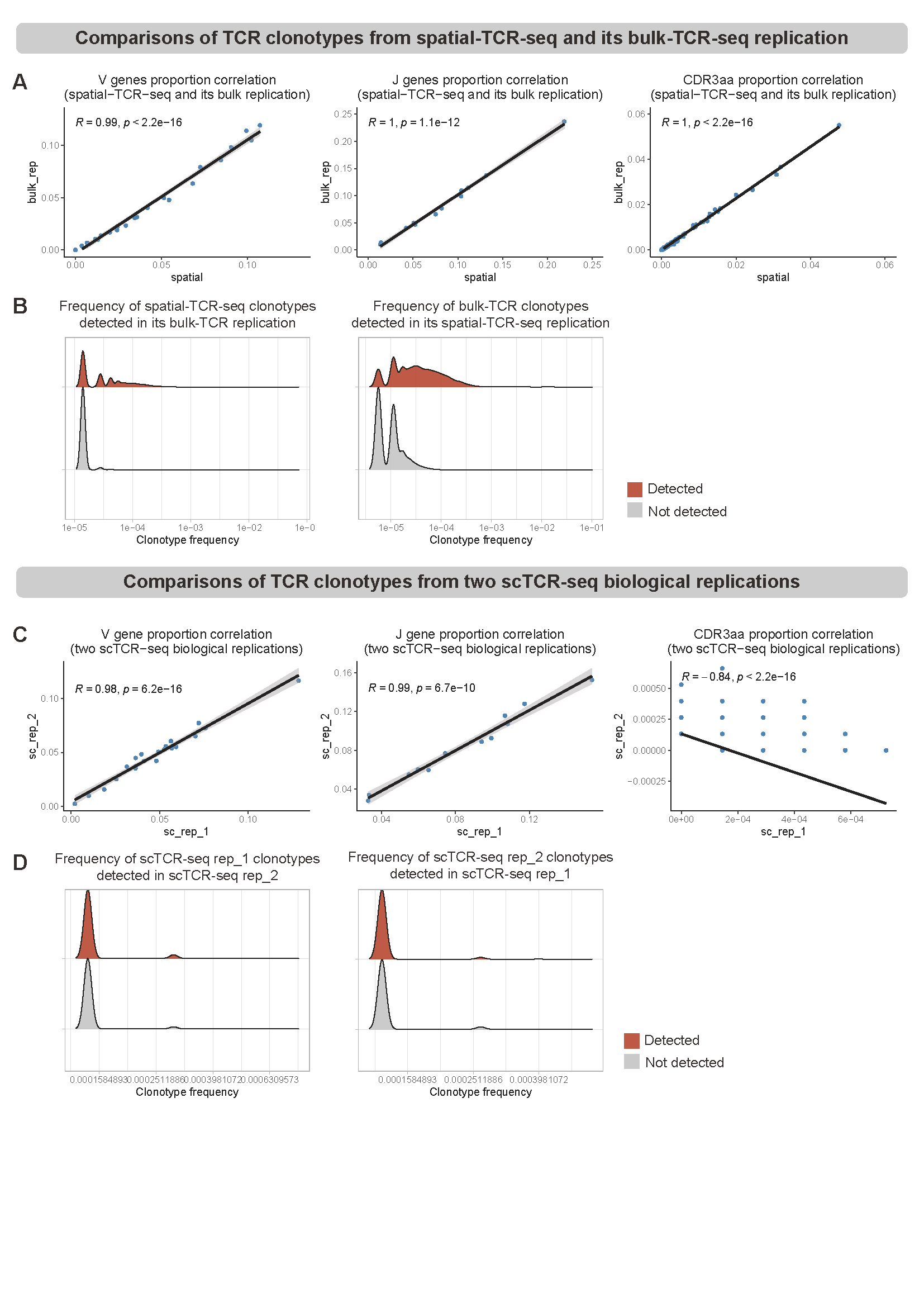


## Figure S33. Comparative analysis of TCR clonotypes among spatial-TCR-seq, matched bulk-TCR-seq replicate, and two scTCR-seq biological replicates

1. Scatter plots showing the correlation of the proportion of VJ genes and CDR3aa between spatial-TCR-eq and its matched bulk-TCR replicate. Significances were calculated with the t-test method and the correlation was calculated with the spearman method.
2. Ridge plots showing the frequency distribution of spatial-TCR-seq derived clonotypes that were detected or not detected in its matched bulk-TCR replicate.
3. Scatter plots showing the correlation of the proportion of VJ genes and CDR3aa between two independent scTCR-seq replicates. Significances were calculated with the t-test method and the correlation was calculated with the spearman method.
4. Ridge plots showing the frequency distribution of clonotypes that were detected or not detected between two independent scTCR-seq replicates.

## Figure S34


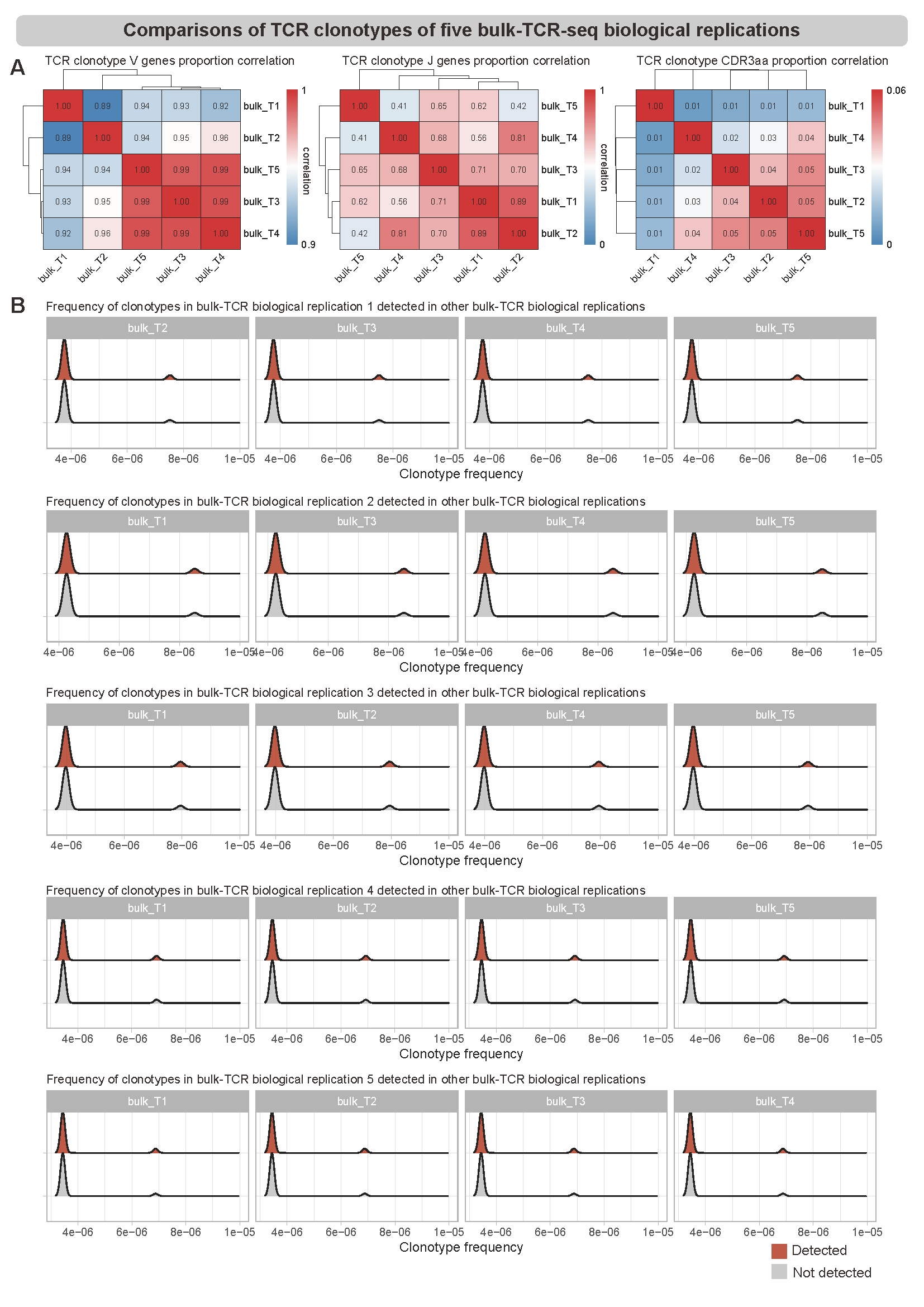


## Figure S34. Comparative analysis of TCR clonotypes among spatial-TCR-seq, matched bulk-TCR-seq replicate, and two scTCR-seq biological replicates

1. Heatmaps showing the correlation of the proportion of VJ genes and CDR3aa among distinct bulk-TCR-seq biological replications. The correlation was calculated with the spearman method.
2. Ridge plots showing the frequency distribution of clonotypes that were detected or not detected among five independent bulk-TCR-seq replicates.

Table S1. primers for TCR-specific amplification (step 1)

| TCR | sequence |
| --- | --- |
| TRAV7-1F1 | Acctcaatagagccagcctg |
| TRAV7-2F1 | Cctcaataaagccagcctgcat |
| TRAV7-3F1 | Ttcacaattcacctcaataaagc |
| TRAV7-4F1 | Cctcaataaggccagcctgcat |
| TRAV7-5F1 | gacaagaaggaaggcagattca |
| TRAV7-6F1 | tgtccatcttctctgatggtg |
| TRAV14-1F1 | Gattcacaatcttcttcaata |
| TRAV14-2F1 | caaccttcttcaataaaagggag |
| TRAV9-1F1 | acccagtggttcaaggagtg |
| TRAV6-1F1 | Cagaggttttgaagctaca |
| TRAV6-2F1 | cagagggtttgaagccacata |
| TRAV6-3F1 | aagggaatcagcagaggctttg |
| TRAV3-1F1 | Tactgaacaagaaagacaaac |
| TRAV21-1F1 | ttcctggctattgcctctgacag |
| TRAV15-1F1 | Tctgtagtcttccagaaat |
| TRAV12-1F1 | acgccactctccataagagcag |
| TRAV13-1F1 | gctctttgcacatttcctcc |
| TRAV13-2F1 | cagtcactaagggacgtcacag |
| TRAV13-3F1 | Gaggtggaagactgacatcctcc |
| TRAV4-1F1 | caggaacaaaggagaatggga |
| TRAV4-2F1 | Aggaacaaaggagaatggaagg |
| TRAV2-1F1 | Gcatctctgtttatctctgctgac |
| TRAV5-1F1 | Ggactcatcgttttactggataag |
| TRAV5-2F1 | agatttatagttttactgaata |
| TRAV5-3F1 | Ttttatttgataagaaaaccaaac |
| TRAV11-1F1 | Agatactcagcaactctggata |
| TRAV10-1F1 | Ttggataagaaagccaaacg |
| TRAV20-1F1 | Agattcgaggtgatattggatag |
| TRAV16-1F1 | Ctctgaactttcagaagcca |
| TRAV8-1F1 | Ctcagagccacccttgacac |
| TRAV8-2F1 | Ctaagagccacccttgacac |
| TRAV1-1F1 | Gtatttctttcttatgttgttttg |
| TRAV19-1F1 | Tcacagttttcttgaacaaaagc |
| TRAV17-1F1 | Aagggctttgaggccgagtttag |
| TRAV18-1F1 | Ctgctaagtttggtgaagcaag |
|  |  |
| TRBV5F1 | Agtcgttttatacctgaatgc |
| TRBV1F1 | cttcccggtgctgattacctgg |
| TRBV26F1 | Actgaaaaacgattctctgctg |
| TRBV2F1 | Gcctcaagtcgcttccaacctc |
| TRBV12F1 | Cccagcagattctcagtccaacag |
| TRBV19F1 | Tctgaaggctatgatgcgtc |
| TRBV29F1 | Cctaaaggatacagggtctc |
| TRBV13F1 | Catgggctgaggctgatccatta |
| TRBV17F1 | Agaagttccaatccagtcggc |
| TRBV4F1 | Ccaaggcgcttctcacctcag |
| TRBV16F1 | Cccaaggaacgattctcagctcag |
| TRBV15F1 | Aggatcgattcaaagctgag |
| TRBV14F1 | Cggatcgattttctgctgtg |
| TRBV31F1 | Caactgaacctctcagcttcc |
| TRBV20F1 | Aattttacccaggaaaaatttc |
| TRBV3F1 | Tttaaggatcagttttcagttg |
| TRBV24F1 | Gtcaagaagagattctcagc |
| TRBV30F1 | Ccaaggacaagtttccaatcag |
| TRBV21F1 | Gtaaaaacatttcagcaaaatg |
| TRBV23F1 | Aggagagattctcagctgtg |
| TRBVF1 | Aggggtacaatgtctccagatc |
| TRBV9F1 | Gtgaccatttctctgcagtgag |
| TRBV10F1 | Aagggtacagtgccttcagaaatg |
|  |  |
| Slide Primers-1 | ACACTCTTTCCCTACACGACGCTCTTCCGATCT |

Table S2. primers for nested amplification (step 2)

| TCR | sequence |
| --- | --- |
| TRAV7-1F2 | CAGACGTGTGCTCTTCCGATCTTagagccagcctgcatgttt |
| TRAV7-2F2 | CAGACGTGTGCTCTTCCGATCTActtccctgcacatcagagac |
| TRAV7-3F2 | CAGACGTGTGCTCTTCCGATCTtaaagccagtctgcatttctc |
| TRAV7-4F2 | CAGACGTGTGCTCTTCCGATCTcatgtttccctgcacatcagag |
| TRAV7-5F2 | CAGACGTGTGCTCTTCCGATCTcctcaataaggccagcctgtat |
| TRAV7-6F2 | CAGACGTGTGCTCTTCCGATCTtgcatgtttccctacacatcag |
| TRAV14-1F2 | CAGACGTGTGCTCTTCCGATCTcacaatcttcttcaataaaagggag |
| TRAV14-2F2 | CAGACGTGTGCTCTTCCGATCTggagaaaaagctctccttgc |
| TRAV9-1F2 | CAGACGTGTGCTCTTCCGATCTgaggctgagttcagcaagag |
| TRAV6-1F2 | CAGACGTGTGCTCTTCCGATCTAggttttgaagctacata |
| TRAV6-2F2 | CAGACGTGTGCTCTTCCGATCTgaagccacatacaataaagaa |
| TRAV6-3F2 | CAGACGTGTGCTCTTCCGATCTctttgaagctacatatgaca |
| TRAV3-1F2 | CAGACGTGTGCTCTTCCGATCTactctctctgaacctcacag |
| TRAV21-1F2 | CAGACGTGTGCTCTTCCGATCTCtctgacagaaagtcaagcacc |
| TRAV15-1F2 | CAGACGTGTGCTCTTCCGATCTtctgtagtcttccagaaatca |
| TRAV12-1F2 | CAGACGTGTGCTCTTCCGATCTCtccataagagcagcagctcct |
| TRAV13-1F2 | CAGACGTGTGCTCTTCCGATCTGcacatttcctcctcccag |
| TRAV13-2F2 | CAGACGTGTGCTCTTCCGATCTgactatatcctcctcccag |
| TRAV13-3F2 | CAGACGTGTGCTCTTCCGATCTcagccagtaacgaaagtcgcagc |
| TRAV4-1F2 | CAGACGTGTGCTCTTCCGATCTAggaacaaaggagaatgggagg |
| TRAV4-2F2 | CAGACGTGTGCTCTTCCGATCTaggagcgctacagcaccctgc |
| TRAV2-1F2 | CAGACGTGTGCTCTTCCGATCTcggaagctcagcactctgag |
| TRAV5-1F2 | CAGACGTGTGCTCTTCCGATCTagaaagccaaacgcttctccc |
| TRAV5-2F2 | CAGACGTGTGCTCTTCCGATCTataagaaatccaaacatttctccc |
| TRAV5-3F2 | CAGACGTGTGCTCTTCCGATCTataagaaaaccaaacacctttc |
| TRAV11-1F2 | CAGACGTGTGCTCTTCCGATCTagatgctaagcacagcacgctg |
| TRAV10-1F2 | CAGACGTGTGCTCTTCCGATCTaagccaaacgattctccctgca |
| TRAV20-1F2 | CAGACGTGTGCTCTTCCGATCTggatagaaatgttaaacgcgtc |
| TRAV16-1F2 | CAGACGTGTGCTCTTCCGATCTgccaaaaagttccatcggac |
| TRAV8-1F2 | CAGACGTGTGCTCTTCCGATCTtccagccagagcagctcc |
| TRAV8-2F2 | CAGACGTGTGCTCTTCCGATCTtgacacctccagccaaagtag |
| TRAV1-1F2 | CAGACGTGTGCTCTTCCGATCTttcctgagccgctcgaatgg |
| TRAV19-1F2 | CAGACGTGTGCTCTTCCGATCTacaaaagcggcaaacacttc |
| TRAV17-1F2 | CAGACGTGTGCTCTTCCGATCTcgagtttaggaagagtaactc |
| TRAV18-1F2 | CAGACGTGTGCTCTTCCGATCTgcaagaaagaacagctccctg |
|  |  |
| TRBV5F2 | CAGACGTGTGCTCTTCCGATCTatgcccagacagctccaagc |
| TRBV1F2 | CAGACGTGTGCTCTTCCGATCTCcacacgggtcactgatacg |
| TRBV26F2 | CAGACGTGTGCTCTTCCGATCTagtgtccttcaaactcacct |
| TRBV2F2 | CAGACGTGTGCTCTTCCGATCTccatttagaccttcagatcac |
| TRBV12F2 | CAGACGTGTGCTCTTCCGATCTtttgatgactatcactctga |
| TRBV19F2 | CAGACGTGTGCTCTTCCGATCTtcgagagaagaagtcatc |
| TRBV29F2 | CAGACGTGTGCTCTTCCGATCTacggaagaagcgggagcatt |
| TRBV13F2 | CAGACGTGTGCTCTTCCGATCTTccctgatggatacaaggcc |
| TRBV17F2 | CAGACGTGTGCTCTTCCGATCTctgctctctctacattggctc |
| TRBV4F2 | CAGACGTGTGCTCTTCCGATCTtcttcagataaagctcatttg |
| TRBV16F2 | CAGACGTGTGCTCTTCCGATCTatgcccaatcagtcgcactc |
| TRBV15F2 | CAGACGTGTGCTCTTCCGATCTatgctaaattcatccttctc |
| TRBV14F2 | CAGACGTGTGCTCTTCCGATCTaggcctaaaggaactaactcc |
| TRBV31F2 | CAGACGTGTGCTCTTCCGATCTaggccgaaggacgaccaattc |
| TRBV20F2 | CAGACGTGTGCTCTTCCGATCTCcatcagtcatcccaacttatc |
| TRBV3F2 | CAGACGTGTGCTCTTCCGATCTaaagaccagatggttcatat |
| TRBV24F2 | CAGACGTGTGCTCTTCCGATCTtaagtgttcctcgaactcac |
| TRBV30F2 | CAGACGTGTGCTCTTCCGATCTccggccaaacctaacattctc |
| TRBV21F2 | CAGACGTGTGCTCTTCCGATCTccctgctaagaaaccatgtacc |
| TRBV23F2 | CAGACGTGTGCTCTTCCGATCTtgcccctccagctcactctg |
| TRBV8F2 | CAGACGTGTGCTCTTCCGATCTatgcacagaggacttcaccttc |
| TRBV9F2 | CAGACGTGTGCTCTTCCGATCTgcccgagggctccttctccatg |
| TRBV10F2 | CAGACGTGTGCTCTTCCGATCTagatacagagctttcccctgac |
|  |  |
| P5 | ACACTCTTTCCCTACACGAC |
| P5-i1 TCR | AATGATACGGCGACCACCGAGATCTACACTATAGCCTACACTCTTTCCCTACACGAC |
| P7-i1 TCR | CAAGCAGAAGACGGCATACGAGATATCACGTTGTGACTGGAGTTCAGACGTGTGCTCTTCCGATC |

References and Notes

1 Ma, Y. & Zhou, X. Spatially informed cell-type deconvolution for spatial transcriptomics. *Nature biotechnology* **40**, 1349-1359, doi:10.1038/s41587-022-01273-7 (2022).

2 Satija, R., Farrell, J. A., Gennert, D., Schier, A. F. & Regev, A. Spatial reconstruction of single-cell gene expression data. *Nat Biotechnol* **33**, 495-502, doi:10.1038/nbt.3192 (2015).

3 Butler, A., Hoffman, P., Smibert, P., Papalexi, E. & Satija, R. Integrating single-cell transcriptomic data across different conditions, technologies, and species. *Nat Biotechnol* **36**, 411-420, doi:10.1038/nbt.4096 (2018).

4 Stuart, T. *et al.* Comprehensive Integration of Single-Cell Data. *Cell* **177**, 1888-1902.e1821, doi:10.1016/j.cell.2019.05.031 (2019).

5 Hao, Y. *et al.* Integrated analysis of multimodal single-cell data. *Cell* **184**, 3573-3587.e3529, doi:10.1016/j.cell.2021.04.048 (2021).

6 Ashburner, M. *et al.* Gene ontology: tool for the unification of biology. The Gene Ontology Consortium. *Nat Genet* **25**, 25-29, doi:10.1038/75556 (2000).

7 Xu, Z. *et al.* Integrative Analysis of Spatial Transcriptome with Single-cell Transcriptome and Single-cell Epigenome in Mouse Lungs after Immunization. *bioRxiv*, 2021.2009.2017.460865, doi:10.1101/2021.09.17.460865 (2021).

8 Jarriault, S. *et al.* Signalling downstream of activated mammalian Notch. *Nature* **377**, 355-358, doi:10.1038/377355a0 (1995).

9 Street, K. *et al.* Slingshot: cell lineage and pseudotime inference for single-cell transcriptomics. *BMC genomics* **19**, 477, doi:10.1186/s12864-018-4772-0 (2018).

10 Aibar, S. *et al.* SCENIC: single-cell regulatory network inference and clustering. *Nat Methods* **14**, 1083-1086, doi:10.1038/nmeth.4463 (2017).

11 Jin, S. *et al.* Inference and analysis of cell-cell communication using CellChat. *Nature communications* **12**, 1088, doi:10.1038/s41467-021-21246-9 (2021).

12 Shugay, M. *et al.* VDJtools: Unifying Post-analysis of T Cell Receptor Repertoires. *PLoS computational biology* **11**, e1004503, doi:10.1371/journal.pcbi.1004503 (2015).

13 Wilkins, M. R. *et al.* Protein identification and analysis tools in the ExPASy server. *Methods Mol Biol* **112**, 531-552, doi:10.1385/1-59259-584-7:531 (1999).

14 Lagattuta, K. A. *et al.* Repertoire analyses reveal T cell antigen receptor sequence features that influence T cell fate. *Nature immunology* **23**, 446-457, doi:10.1038/s41590-022-01129-x (2022).

15 Zhang, Z., Xiong, D., Wang, X., Liu, H. & Wang, T. Mapping the functional landscape of T cell receptor repertoires by single-T cell transcriptomics. *Nat Methods* **18**, 92-99, doi:10.1038/s41592-020-01020-3 (2021).

16 Atchley, W. R., Zhao, J., Fernandes, A. D. & Drüke, T. Solving the protein sequence metric problem. *Proceedings of the National Academy of Sciences of the United States of America* **102**, 6395-6400, doi:10.1073/pnas.0408677102 (2005).
